# European Stroke Organisation (ESO) guideline on visual impairment in stroke

**DOI:** 10.1177/23969873251314693

**Published:** 2025-05-22

**Authors:** Fiona J Rowe, Lauren R Hepworth, María Begoña Coco-Martin, Celine R Gillebert, Luis Leal-Vega, Anja Palmowski-Wolfe, Eleni Papageorgiou, Stephen James Ryan, Karolina Skorkovska, Anne Hege Aamodt

**Affiliations:** 1 Institute of Population Health, University of Liverpool, Liverpool, UK; 2 Department of Medicine, Dermatology and Toxicology, University of Valladolid, Valladolid, Spain; 3 Department Brain and Cognition, Leuven Brain Institute (LBI), KU Leuven, Leuven, Belgium; 4 University Eye Hospital Basel, University of Basel, Basel, Switzerland; 5 Department of Ophthalmology, University Hospital of Larissa, Larissa, Greece; 6 Department of Neurology, Oslo University Hospital, Oslo, Norway; 7 Department of Optometry and Orthoptics, Masaryk University, Brno, Czech Republic; 8 Department of Neuromedicine and Movement Science, the Norwegian University of Science and Technology, Trondheim, Norway

**Keywords:** Guideline, systematic review, stroke, vision, visual impairment, ocular stroke, eye movements, visual fields, visual perception, visual neglect, screening, treatment

## Abstract

Visual impairment due to stroke is common. However, controversy exists on how best to screen for visual impairment, the timing at which to screen, and on the optimal management of the varying types of visual impairment. This European Stroke Organisation (ESO) guideline provides evidence-based recommendations to assist clinicians in decision-making on screening methods, timing of screening and assessment and management options in adult stroke survivors. The target audience for this guideline is health care providers involved in stroke care from prehospital screening, in stroke units and rehabilitation centres, ophthalmological departments and community stroke care, and for stroke survivors and care givers. The guideline was developed according to the ESO standard operating procedure and the Grading of Recommendations, Assessment, Development and Evaluation (GRADE) methodology. The working group identified relevant clinical questions, performed systematic reviews and, where possible, meta-analyses of the literature, assessed the quality of the available evidence and made specific recommendations. Expert consensus statements were provided where insufficient evidence was available to provide recommendations based on the GRADE approach. We found evidence of acceptability and feasibility of early visual screening within 1 week of stroke onset. We describe the accuracy of various vision screening tools at pre-hospital and hyper/acute stages as well as specialist vision assessment. We suggest vision screening in all patients with stroke to improve detection of their visual problems We describe a range of treatment options for visual impairment post-stroke across the typical categories of impaired central vision, ocular stroke (central retinal artery occlusion), eye movements, visual fields, visual neglect and visual perception. This guideline highlights specific areas where robust evidence is lacking and where further definitive randomised controlled trials and diagnostic accuracy studies are required.

## Plain language summary

Up to three quarters of stroke survivors have visual problems with most (about 60%) being caused by the stroke. The other visual problems are usually related to already existent eye conditions. When a stroke happens, often people are not aware their vision can be affected and, frequently, visual impairment is not detected or suspected by clinical and medical teams. The types of visual impairment that can occur after stroke include eye movement problems (causing double or jumbled vision), a reduction in how well we see things clearly (our central vision), a loss in field of vision (our peripheral vision), visual neglect (lack of attention to part of the visual surroundings) and visual perceptual problems (how we process what we see, such as colour and recognition of people and objects). We don’t know how we should best screen for visual impairment or when to do this. Subsequently, how best should we manage these visual problems. In these guidelines we have carefully reviewed studies that give results on vision screening for stroke survivors and results on management of various types of visual impairment. We found a number of vision screening methods than can be used very early (within days) after stroke onset to improve the detection of visual impairment. We also found a variety of treatment options that can be recommended dependent on the different types of visual impairment that occur. In reviewing studies for this guideline, we have also found areas where evidence for vision care is poor. Therefore, we have given suggestions for future research studies that will improve the care we provide for stroke survivors with visual impairment.

For diagnosis, we recommend vision screening to improve detection of visual problems in stroke survivors. Vision screening should be undertaken using a validated vision screening tool or by specialist vision team assessment. Early vision screening should be undertaken within 3–4 days after onset of stroke.

For treatment, we recommend compensatory interventions of visual scanning/visual search to aid adaptation to visual field loss after stroke. We recommend thrombolysis within 4.5 h of stroke onset to aid recovery of visual function after eye stroke. We suggest early management options to improve visual acuity should be offered as soon as possible after stroke onset such as wearing glasses. We recommend referral to specialist eye services for the targeted management of eye movement disorders. We recommend individualised intervention targeted at the specific type of visual neglect or visual perception deficit that has arisen.

Overall, we recommend close collaboration between stroke teams (particularly occupational therapy), neuropsychology and eye care teams (orthoptics, ophthalmology, optometry) for targeted management of visual neglect, and clinicians should provide appropriate information, resource materials and vision aids.

## Graphical abstract

**Graphical abstract:**
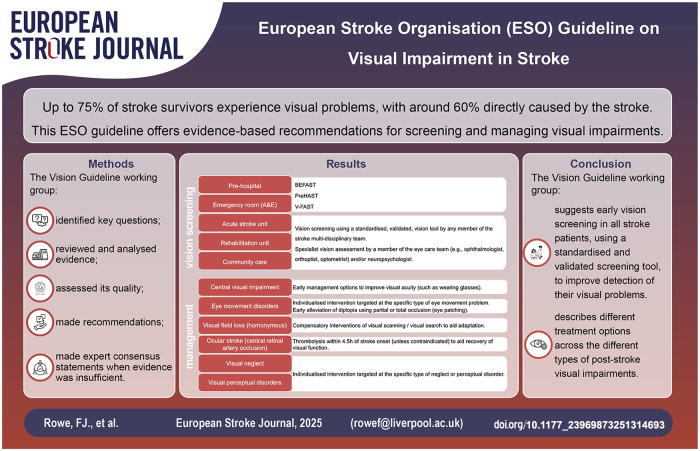

## Table of key recommendations/suggestions of the Vision Guideline

**Table:** 

**Diagnosis**
Undertake vision screening of all stroke survivors to improve detection of visual problems in stroke survivors.
Undertake vision screening using a standardised, validated vision screening tool or by specialist eye team assessment.
Undertake early vision screening within 3–4 days post onset of stroke.
**Treatment**
Treat stroke survivors with compensatory interventions of visual scanning/visual search to aid adaptation to visual field loss after stroke.
Treat ocular stroke (central retinal artery occlusion) with thrombolysis within 4.5 h of stroke onset (if there are no contraindications) to aid recovery of visual function.
Provide early management options to improve visual acuity.
Refer to specialist eye services for the targeted management of eye movement disorders.
Provide individualised intervention targeted at the specific type of visual neglect or visual perception deficit that has arisen.
Establish close collaboration between stroke teams (particularly occupational therapy), neuropsychology and eye care teams (orthoptics, ophthalmology, optometry) for targeted management of visual impairment.
Provide appropriate vision-related information, resource materials and vision aids to stroke survivors and their care givers

## Table of Contents

**Introduction---------------------------------------------------------------------------------------------------------------------4**

**Methods---------------------------------------------------------------------------------------------------------------------4**

Composition and approval of the Module Working Group--------------------------------------------------------------------------------------------4

Development and approval of clinical questions 4

**Literature search---------------------------------------------------------------------------------------------------------------------5**

Data analysis---------------------------------------------------------------------------------------------------------------------5

Evaluation of the quality of evidence and formulation of recommendations--------------------------------------------5

Drafting of the document, revision and approval--------------------------------------------------------------------------------6

**Results---------------------------------------------------------------------------------------------------------------------7**

*PICO 1* For adults with visual problems due to stroke, does routine use of vision screening, compared to no routine vision screening, improve detection rate? 7

*PICO 2* For adults with visual problems due to stroke, does early assessment within one week of stroke admission, compared to later assessment, improve activities and quality of daily life? 10

*PICO 3* For adults with visual field loss due to stroke, does identification of visual field loss by vision screening or specialist eye team, compared to routine stroke screen, improve detection rate and activities/quality of life? 12

*PICO 4* For adults with central vision impairment due to stroke, does identification of visual acuity loss by vision screening or specialist eye team, compared to routine stroke screen, improve detection rate and activities/quality of life?---------------------------------------------------------------------------------------------------------------------15

*PICO 5* For adults with eye movement disorders due to stroke, does identification of strabismus and/or ocular motility deficit loss by vision screening or specialist eye team, compared to routine stroke screen, improve detection rate and activities/quality of life?---------------------------------------------------------------------------------------------------------------------18

*PICO 6* For adults with visual perceptual disorders due to stroke, does identification of visual perceptual disorders by screening proforma/tool or specialist team, compared to routine stroke screen, improve detection rate and activities/quality of life?---------------------------------------------------------------------------------------------------------------------20

*PICO 7* For adults with visual neglect due to stroke, does identification of visual neglect by screening proforma/tool or specialist team, compared to routine stroke screen, improve detection rate and activities/quality of life?---------------------------------------------------------------------------------------------------------------------22

*PICO 8* For adults with homonymous visual field loss due to stroke, does compensatory, substitute or restitutive intervention, compared to no intervention, improve activities and quality of daily life?----------------------------------------------------------------25

*PICO 9* For adults with ocular stroke (central retinal artery occlusion), does compensatory, substitute or restitutive intervention, compared to no intervention, improve activities and quality of daily life?----------------------------------------------------------------------------------------------32

*PICO 10* For adults with central vision impairment due to stroke, does compensatory, substitute or restitutive intervention, compared to no intervention, improve activities and quality of daily life?------------------------------------------------------------------------------------------34

*PICO 11* For adults with eye movement disorders due to stroke, does compensatory, substitute or restitutive intervention, compared to no intervention, improve activities and quality of daily life?-------------------------------------------------------------------------------37

*PICO 12* For adults with visual neglect due to stroke, does compensatory, substitute or restitutive intervention, compared to no intervention, improve activities and quality of daily life?---------------------------------------------------------------------------------------------------39

*PICO 13* For adults with other visual perceptual disorders due to stroke, does compensatory, substitute or restitutive intervention, compared to no intervention, improve activities and quality of daily life?---------------------------------------------------------------------------------------------------59

**Discussion---------------------------------------------------------------------------------------------------------------------61**

**References---------------------------------------------------------------------------------------------------------------------68**

## Introduction

Visual impairment is common post-stroke and includes loss or impairment of central and peripheral vision, eye movement disorders, visual neglect and visual perception deficits.^1^ Reported prevalence is about 75% and incidence about 60% of stroke survivors.^2^ Despite the importance of vision in daily life, visual impairment post-stroke is under-recognised and under detected/diagnosed. Provision of care for visual impairment post-stroke is ad hoc and lacking standardisation with considerable variation in diagnosis and management globally.^3,4^ Visual impairment post-stroke is absent from many international guidelines for stroke care. In recent years, more research in the field of visual impairment post-stroke has reported on aspects of screening and detection, and there is growth in intervention studies and trials. However, there is no up-to-date overview of evidence of visual impairment post-stroke to provide guidance on this important function. As clinicians may benefit from a synthesis of the available research that allows evidence-based, or expert informed, guidance on post-stroke visual impairment, the European Stroke Organisation (ESO) commissioned this guideline. The intention of this guideline is to provide a useful resource for health professionals and researchers from multiple disciplines across stroke, neurology and ophthalmology, as well as policy makers, stroke survivors and care givers. Recognising that the potential scope of this guideline was broad, we chose to focus on two specific areas of clinical importance: diagnosis and management.

The guideline followed best practice and adhered to the Standard Operating Procedure (SOP) of the ESO Guideline Group.^5,6^ The methods used to formulate the recommendations and consensus statements are described later in the text. However, there are certain aspects of the approach that are worthy of mention early in the guideline and will be discussed here. In planning the work, we were keen that we represent many of the clinical disciplines involved in managing people living with stroke and subsequent post-stroke visual impairment. In this guideline we took an inclusive approach. We defined the concept of post-stroke visual impairment as all problems in visual function that occur following a stroke, irrespective of whether ischaemic or haemorrhagic.

As we focussed on both diagnosis and management of visual impairment following stroke, we did not restrict the scope to those areas where we knew we would find high-quality trials. We formulated the questions as Population, Intervention, Comparator and Outcomes (PICOs). We planned that where an evidence-based recommendation was not possible, we would provide an expert opinion taking in consideration all the available information and drawing on the experience and knowledge of the multidisciplinary writing group.

For all PICO questions, we pre-specified strict inclusion criteria around study method (randomised controlled trials (RCTs) and observation cohorts), population size, duration of follow-up and study design. Anticipating that some areas may have few included studies, as a final part of the guideline writing process, we used the available evidence to select key research questions that should be a priority for future studies.

## Methods

### Composition and approval of the Module Working Group

These guidelines were initiated by the ESO. One chairperson (Fiona Rowe) was selected to assemble and coordinate the Guideline Module Working Group (MWG). The final group contained ten experts and two chairpersons (Fiona Rowe and Anne Hege Aamodt). The composition of the MWG was designed to include those disciplines involved in the care of people living with post-stroke visual impairment and comprised multidisciplinary expertise from stroke medicine, neurology, neuropsychology, ophthalmology and orthoptics.

Attention was given to achieving diversity in terms of sex and geography. The ESO Guideline Board and Executive Committee reviewed the intellectual and financial disclosures of all MWG members and approved the composition of the group. The full details of all MWG members and their disclosures is included in Supplemental Table 1.

### Development and approval of clinical questions

This guideline was prepared according to the ESO SOPs,^5^ which are based on the Grading of Recommendations, Assessment, Development and Evaluations (GRADE) framework.^6^ A list of abbreviations for the guideline can be found in Supplemental Table 2. The MWG developed a list of topics and corresponding questions of greatest clinical interest. Questions were formatted using the PICO approach and reviewed by two external reviewers as well as members of the ESO Guideline board and Executive Committee (five reviewers in total). The MWG developed a list of corresponding outcomes of clinical interest. These were rated by members of the MWG as critical, important or of limited importance according to GRADE criteria. Final decision on outcomes used a Delphi approach in which the MWG voted in a closed survey to identify which outcomes were of highest priority on a 9-point scale from 1–3 ‘not important’ to 7–9 ‘critical’. Outcomes rated as ‘critical’ were chosen for each PICO. These were subsequently approved by the ESO Guidelines Board and Executive Committee. Results of the outcomes rating for each PICO question are included in the Supplemental Table 3.

## Literature search

For each PICO question, search terms were developed by the MWG and guideline methodologist. Where a validated search strategy was available, this was used or adapted. Where there was a relevant systematic review on the question of interest, the corresponding search strategy and results were used and updated as necessary. We found relevant systematic reviews for all PICOs and the searches for this guideline dated from their search dates. Search strategies and details of previous systematic reviews^7–23^ are described in Supplemental Table 4.

The search was performed by the ESO Guideline methodologist. The following databases were searched: MEDLINE, Scopus, CINAHL and AMED from dates of prior systematic reviews (earliest January 2011) to March 2023. Reference lists of review articles, the authors’ personal reference libraries and previous guidelines were also searched for additional relevant records. Further, we noted potentially relevant ongoing studies for future reference by searching relevant trials registries such as ClinicalTrials.gov.

Search results were uploaded into the web-based Covidence platform (Health Innovation, Melbourne, Australia) for assessment by the MWG. Screening was conducted in a two-step process. For each PICO two or more MWG members were assigned to independently screen initially the titles and abstracts of publications registered in Covidence and then in the second step to assess the full text of studies determined to be potentially relevant. All disagreements were resolved by a third MWG member.

We prioritised RCTs but where data were limited, or RCT study design not relevant, we also considered health registry data analyses and large observational studies. We prespecified that studies would have to include information on a minimum of 20 adult (>18 years) stroke patients in order to allow some assumption on a reliable effect. The MWG decided that smaller studies should be considered proof of concept (unless sample size was formally powered by sample size calculation) and are more prone to publication bias. We considered only studies in humans. We included studies comprising non-stroke aetiologies but >50% were of stroke cause. We excluded publications with only conference abstracts available and non-English publications where translation was not possible by the MWG.

The recommendations provided herein address the diagnosis and management of visual impairment across all stages of stroke presentation and follow-up (hyperacute, acute, subacute and chronic), acknowledging the evolving needs of stroke survivors throughout their care journey. Across the PICOs, we refer to vision screening and specialist visual assessment. Vision screening involves screening for visual impairment using vision checklists or more detailed vision screening proformas/tools and undertaken by any member of the stroke multi-disciplinary team. We refer to proformas (i.e. standardised forms) and tools interchangeably in PICOs 1–7. Specialist vision assessment indicates visual assessment by a member of the eye care team (e.g. ophthalmologist, orthoptist, optometrist) and/or neuropsychologist – the latter particularly for persistent visual neglect and visual perceptual disorders.

### Data analysis

Data extraction and analysis was performed by the MWG. In the case that relevant data were not reported in an eligible study, the corresponding author was contacted. If no answer was received, data were considered as missing.

Results were presented as estimates of effect with associated 95% confidence intervals (95%CIs).

Calculation of combined means was by:

$$x_{c}=\;\frac{m*x_{a}+\;n*x_{b}}{m\;+\;n}$$

where:

x_a_ = the mean of the first group,

m = the number of items in the first group,

x_b_ = the mean of the second group,

n = the number of items in the second group,

x_c_ the combined mean.

Calculation of combined standard deviations with unequal sample sizes was by:

$$\mathrm{Average}\;S.D.=\;\surd \frac{\left(\begin{array}{l} \left(n1-1\right)s12\;+\;\left(\mathrm{n2}-1\right)\mathrm{s22}\;+\;\ldots \;\\ +\;\left(\mathrm{nk}-1\right)\mathrm{sk2} \end{array}\right)}{\left(n1+n2\;+\;\ldots \;+\;\mathrm{nk}\;-\;k\right)}\;$$

where:

n_k_ = Sample size for k^th^ group

s_k_ = Standard deviation for k^th^ group

k = Total number of groups

### Evaluation of the quality of evidence and formulation of recommendations

For each PICO question, and each outcome, the following were considered: risk of bias based on the type of available evidence (randomised or observational studies); considerations on inconsistency of results; indirectness of evidence, imprecision of results and other possible bias. For RCTs, the assessment used the standard Cochrane tool.^24,25^ In the evidence synthesis, we did not use an overall quality ‘score’ as such an approach is now discouraged. The classification of low or high risk of bias was performed by the assessors at individual study level. For each PICO question, the quality of evidence was rated using the GRADEpro Guideline Development Tool (McMaster University, 2015; developed by Evidence Prime, Inc.) using guidelines for non-pooled data as necessary.^6,26,27^ Final quality ratings were categorised as high, moderate, low or very low.

The methods underpinning the test accuracy synthesis differ in some regards from the standard synthesis of trials. In particular, the application of GRADE to diagnostic test accuracy is not as well developed as it is for synthesis of intervention studies. In this quality assessment, we therefore considered risk of bias and applicability using the QUADAS-2 (Quality Assessment of Diagnostic Accuracy Studies) tool, we considered internal consistency through visual inspection of forest plots and considered the precision of the summary estimate.^28^ More detailed descriptions of test accuracy synthesis and reporting are available from the Cochrane Library and others.^24^

GRADE and QUADAS assessments were performed within writing subgroups and then shared with the complete MWG for discussion and consensus. Text was discussed in open forum through monthly team calls and using Microsoft Teams shared files, and members of the complete MWG then voted on the text using a Delphi approach. Complete consensus was required for the recommendation statements, and text was revised until consensus was reached. The direction, strength and formulation of the recommendations were determined according to the GRADE evidence profiles and the ESO SOPs.^5,6^

### Basis for recommendations:

**Table:** 

Strength of recommendation	Balance of desirable and undesirable consequences	Recommendation formatting
Strong recommendation for intervention	The desirable consequences clearly outweigh the undesirable consequences in most settings	‘We recommend’
Strong recommendation against intervention	The undesirable consequences clearly outweigh the desirable consequences in most settings	‘We recommend . . . not’
Weak recommendation for intervention	The desirable consequences probably outweigh the undesirable consequences in most settings	‘We suggest’
Weak recommendation against intervention	The undesirable consequences probably outweigh the desirable consequences in most settings or when the balance between desirable and undesirable consequences is closely balanced or uncertain	‘We suggest . . . not’
Ungraded consensus-based statement	The desirable consequences probably outweigh the undesirable consequences in most settings, but there is little evidence	‘We suggest’

Finally, expert consensus statements were added whenever the MWG considered that there was insufficient evidence available to provide evidence-based recommendations and where practical guidance is needed for routine clinical practice. The expert consensus statements were based on voting by all expert MWG members using a Delphi approach to reach consensus. Importantly, these expert consensus statements should not be regarded as evidence-based recommendations, since they only reflect the opinion of the MWG.

### Drafting of the document, revision and approval

Each PICO question was addressed in distinct sections, in line with the updated ESO SOP.^5^

First, ‘Analysis of current evidence’ summarised current pathophysiological considerations followed by a summary and discussion of the results of the identified RCTs and other studies.

Second, ‘Additional information’ was added when more details on the studies referred to in the first section were needed to provide information on key subgroup analyses of the included studies, on ongoing or future RCTs, and on other studies which can provide important clinical guidance on the topic.

Third, a recommendation or expert consensus statement was added dependent on the level of evidence available.

The completed guideline document was proofed several times by all MWG members and modified until agreement was reached on the full guideline content. The final submitted document was peer-reviewed by two external reviewers, two members of the ESO Guideline Board and one member of the Executive Committee.

## Results

### DIAGNOSIS

***PICO 1:* For adults with visual problems due to stroke, does routine use of vision screening, compared to no routine vision screening, improve detection rate?**

### Analysis of current evidence

In this PICO we considered the outcome of vision assessment and, in particular, vision screening options to determine if their use improves detection of visual problems due to stroke. As upwards of 40% of stroke survivors with confirmed visual impairment do not, or cannot, report visual symptoms, it is important that detection of presence/absence of visual impairment for adults with stroke does not rely solely on patient-reported visual symptoms.^29^ For the purposes of this PICO, we considered any point in the stroke pathway. However, we were interested, particularly, in the hyperacute and acute settings as early vision screening is recommended in international stroke best practice statements and clinical guidelines (e.g. ICSWP 2023, NICE 2023).^30,31^

We found eight studies that compared vision screening tools/tests to no routine vision screening or alternative stroke screening options (Supplemental Table 5.1).^32–39^ These studies had differing populations, screening tools and outcomes, and were therefore grouped by stage of stroke screening: pre-hospital and acute care. Study design was diagnostic accuracy test, cohort and cross-sectional with a median sample size of 100 (range 43–736; mean 204.1, SD 236.4). None were randomised controlled trials. Only two studies compared vision screening to no routine screening. For pre-hospital screening, two studies were identified for screening of visual impairment in stroke events in the prehospital setting.^32,33^ These reported the use of BEFAST (Balance, Eyes, Face, Arm, Speech, Time) versus FAST (Face, Arm, Speech, Time) test, and V-FAST (Vision-FAST) versus National Institute of Health Stroke Scale (NIHSS) checklist.^33^

**Figure 1.1.**
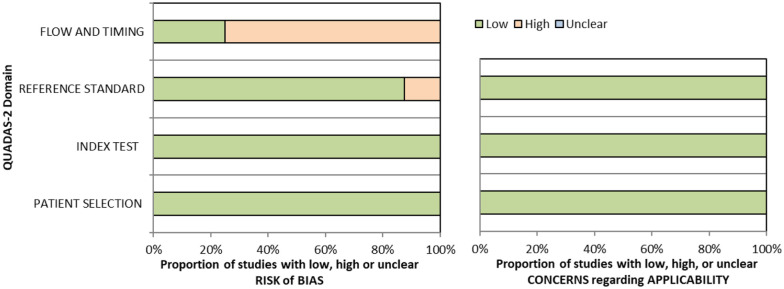
QUADAS domain for PICO 1.

**Figure 1.2.**
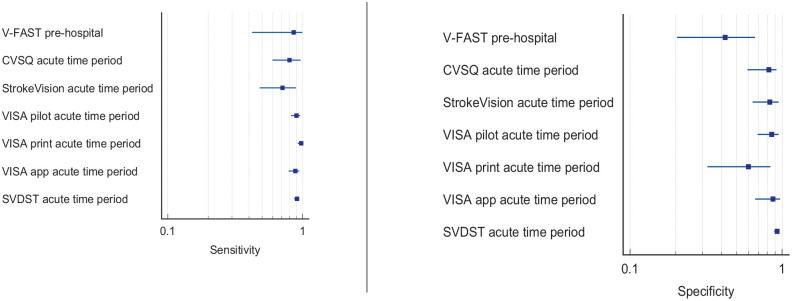
Sensitivity and specificity forest plots for PICO 1.^33,34,36,38,39^

Six studies were identified for screening of visual impairment compared to alternative vision screening in hospitalised stroke survivors.^34–39^ Vision screening was undertaken using a questionnaire; Cerebral Vision Screening Questionnaire (CVSQ),^34^ iPad applications (Visual Impairment Screening Assessment (VISA),^35^ StrokeVision,^36^ Melbourne Rapid Field-Neural (MRFn))^37^ and paper-based screening tools (VISA,^35,38^ Stroke and Vision Defect Screening Tool (SVDST)).^39^ Overall, sensitivity and specificity results were available for seven of the above studies. The majority of studies had a high risk of bias due primarily to being non-RCT design but low risk of bias on QUADAS assessment. Limitations included study heterogeneity, unblinded interpretation of test results and limited information on complete or missing data. Table 1.1 and Figure 1.1 show the QUADAS assessment of diagnostic accuracy of vision screening tools. Figure 1.2 shows forest plots of diagnostic accuracy. Sensitivity and specificity for VFAST were 85 and 42% respectively.^33^ For hospital vision screening tools, averaged sensitivity and specificity were 87.3% and 81.8% respectively.^34–39^

**Table 1.1.** Summary of findings for PICO 1. For adults with visual problems due to stroke, does routine use of vision screening, compared to no routine vision screening, improve detection rate? Assessment of the diagnostic accuracy of vision screening for diagnosis of post-stroke visual impairment. Participants: Stroke survivors. Settings: Variety (pre-hospital, acute and out-patient). Intervention: Vision screening or specialist visual assessment. Reference standard: No routine screen or alterative vision screening.

Test	Summary sensitivity Summary specificity	*N* participants/*N* with visual impairment	QUADAS-2
Versus no routine screen			
V-FAST pre-hospital^33^	0.857 (95%CI: 0.421–0.996) 0.421 (95%CI: 0.203–0.665)	One study 43/22	Medium^a^
Versus alternative vision screening			
CVSQ acute time period^34^	0.798 (95%CI: 0.598–0.965) 0.817 (95%CI: 0.593–0.917)	One study 461/444	Medium^a^
MRFn acute time period^37^	0.93 0.83	One study 60/41	Medium^a^
SVDST acute time period^39^	0.911 (95%CI: 0.864–0.945%) 0.9257 (95%CI: 0.888–0.954%)	One study 99/19	Medium^a^
StrokeVision acute time period^36^	0.71 (95%CI: 0.48–0.89) 0.83 (95%CI: 0.64–0.95)	One study 48/19	Medium^b^
VISA acute time period^35,38^	VISA (pilot), VISA (print and app)VISA pilot 0.9024 (95%CI: 0.8168–0.9569) 0.8529 (95%CI: 0.6894–0.9505)	Two studies (three groups) 317/245 (116/82)	Low
	VISA print 0.9767 (95%CI: 0.9185–0.9972) 0.60 (95%CI: 0.3229–0.8366)	(101/86)	
	VISA app 0.8831 (95%CI: 0.7897–0.9451) 0.8696 (95%CI: 0.6641–0.9722)	(100/77)	

CVSQ: Cerebral Vision Screening Questionnaire; MRFn: Melbourne Rapid Field-neural; SVDST: Stroke Vision Defects Screening Tool; V-FAST: Vision, Face, Arms, Speech, Time; VISA: Vision Impairment Screening Assessment.

a Downgraded due to potential risk of bias on flow of timing.

b Downgraded due to potential risk of bias on flow of timing and reference standard.

Detection rate data were available in 14 studies (Supplemental Table 5.2),^1,35,40–51^ with a median sample size of 73.5 (range 23–88,664; mean 6596.5, SD 23,623.6). Overall, detection rate of visual impairment in stroke survivors, across variable time periods of pre-hospital to chronic stroke stages was a mean of 64.6% (SD 28.8; median 70.5%, range 11.7–96.5%). Variable detection rates were due to heterogeneous study designs, populations (e.g. formal stroke screening programmes versus referrals based on clinician suspicion; general stroke cohorts versus specific stroke types or area of brain), and visual impairment differences (e.g. inclusion of any visual impairment versus specific types such as neglect or hemianopia).

### Additional information

For this PICO, we included outcomes that were rated as critical by the writing group, including sensitivity, specificity and detection rate. We did not include outcomes of false positives, false negatives, positive and negative predictive values and units of assessment. However, these are important considerations for vision screening and information on these outcomes are reported for some diagnostic accuracy studies. When considering patient preferences and values, stroke survivors are quite likely to be willing to have early vision testing, whether screening or specialist assessment, as it is not time consuming and there is no risk involved. The use of vision screening is better to detect the presence of vision problems than without such screening. Furthermore, there is a higher likelihood of undesirable effects without early vision screening, for example, delayed diagnosis of visual impairment or misdiagnosis. This has implications for rehabilitation but in some instances also for treatment and survival. For example, where visual impairment is the only sequelae of stroke, accurate diagnosis of this, and the association of cause being shown to be stroke, is imperative to manage the underlying condition to prevent further and potentially catastrophic strokes.

In reference to vision screening where a positive result may trigger a more detailed assessment (or referral for such) it is important to detect as many cases as possible with potential visual impairment in order to optimise stroke rehabilitation. This applies even if it risks unnecessary added vision testing for some. Here, sensitivity may be preferred over specificity.

Screening with a formal vision screening tool/test/checklist (currently available: pre-hospital = BEFAST, V-FAST^32,33^; in-patient/community = CVSQ, MRFn, SVDST, VISA^34,35,37–39^), particularly in in-hospital settings, consistently detects more visual problems than no visual screening with high sensitivity and specificity across the range. There is a time trade-off versus precision for some. For example, checklists that are used as an adjunct to FAST (e.g. V-FAST, BEFAST) are quick to complete and, hence, are appropriate for pre-hospital and emergency room settings. However, they are targeted at assisting decision-making on stroke detection (stroke or other diagnosis) with emphasis on posterior circulation stroke. There is added importance in identifying visual impairment caused by posterior circulation stroke (because of the potential absence of other neurological sequelae) or detection of ocular stroke (central retinal artery occlusion) within 4 h of stroke onset, to facilitate access to timely thrombolysis. As a rapid checklist, they may miss a visual impairment and thus, lack precision/accuracy. Vision screening tools are distinct from rapid detection checklists so take longer to administer but provide more testing methods and greater accuracy of detection of visual impairment. BEFAST, CVSQ, SVDST, VISA and V-FAST are available free of charge for all clinical use and publicly funded research (accessible from: www.befast.org; www.uni-saarland.de/fileadmin/upload/lehrstuhl/kerkhoff/Materialien_für_Diagnostik_Therapie/CVSQ.pdf; www.aci.health.nsw.gov.au/networks/ophthalmology/vision-defect-in-stroke; www.vision-research.co.uk). The addition of vision components to pre-hospital stroke screening may improve the detection rate for FAST-negative strokes.^32,33^ Further, higher detection rates can be achieved on acute stroke and rehabilitation units with more robust measures such as specialist eye examination by orthoptists/ophthalmologists. The vision screening tools were demonstrated to be feasible at various stages from prehospital to stroke unit acute care. Acceptability by staff and patients was observed with screening possible in acute settings and often within 3 days of stroke onset.^2^ At least 40% of stroke survivors cannot or do not report visual symptoms despite presence of a visual impairment and hence, the clinician cannot rely on patient symptom-reporting as an indicator of presence or absence of visual impairment.^29^ Therefore, formal vision screening of stroke survivors is needed to improve detection rate.


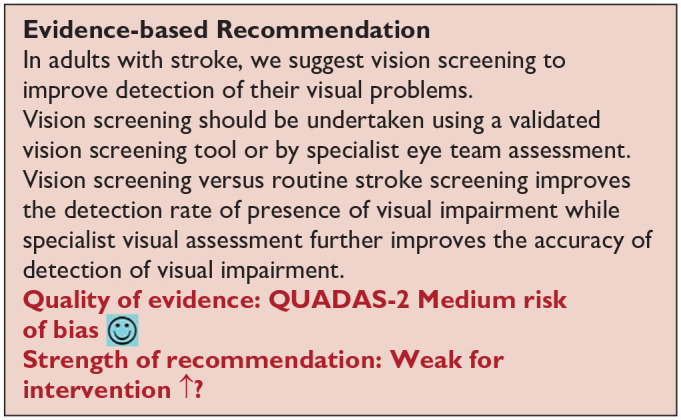


***PICO 2:* For adults with visual problems due to stroke, does early assessment within one week of stroke admission, compared to later assessment, improve activities and quality of daily life?**

### Analysis of current evidence

In this PICO, we consider the timing of vision screening (vision screening rather than routine stroke screening) and, in particular, the impact of early (within 1 week of stroke onset) versus later vision assessment, on activities of daily life and quality of life parameters. We were interested in the acute setting as early vision screening is recommended in international stroke best practice statements and clinical guidelines (e.g. ICSWP 2023, NICE 2023).^30,31^ We found no studies that directly compared early to later vision screening/assessment.

### Additional information

We found four studies (two cohort, one cross sectional and one online questionnaire) that were relevant to the PICO topic but not completely aligned with the original question (Supplemental Table 6.1).^2,52–54^ Of these, three were patient population studies with median sample size of 349 (range 245–1295; mean 629.7, SD 578.5).^2,52,53^ Median number of stroke survivors completing visual screening was 245 (range 22–1033; mean 433.3, SD 531.2).

In assessing the evidence for this PICO, there are some considerations to review. For this PICO, we included those outcomes rated as critical by the writing group. We prioritised length of stay in the hospital and time to visual screening/assessment.

Two studies reported length of stay in stroke survivors with visual impairment.^2,52^ Averaged mean of length of stay for both studies was 49.69 days (SD 67.84). One study reported mean length of stay for stroke survivors with normal visual function of 13.5 days (SD 45.9).^2^

Overall, length of stay was significantly longer for stroke survivors who had visual impairment. However, this is impacted by other factors as length of stay is also significantly associated with greater stroke severity as indicated in these studies. Thus, a causal association cannot be implied. As stroke severity and visual problems are correlated, it cannot be followed that early assessment of vision will impact discharge. However, it may help predict earlier discharge. Further research is needed to that regard.

All studies reported results relevant to time of visual screening. An epidemiology study with an aim of exploring feasibility of early visual assessment reported visual assessment within 4 days for over 70% of stroke survivors.^2^ The median for completing an initial visual screen was 3 days (IQR 2) and median for completing a full specialist visual assessment was 4 days (IQR 7). Norup et al. reported 81.8% were referred to the visual team for additional rehabilitation on average 8 days (SD 8.30) after admission.^52^ The importance of early visual assessment was confirmed in an international survey of current practice among orthoptists with typical overall follow-up of vision care being less than 3 months with 35.5% of orthoptists seeing patients within 2 weeks of stroke onset and 55.5% by 1 month post stroke.^54^ Räty et al. specifically studied occipital lobe stroke survivors with isolated visual symptoms.^53^ Only 20.8% arrived at the hospital within the 4.5 h therapeutic time window of thrombolysis. Delays were often caused by either not identifying the problem correctly or spending too long on preceding specialist examinations. This resulted in missed therapeutic opportunities to treat these stroke survivors who typically present with visual field defects. This indicates the importance of immediate recognition of visual symptoms associated with stroke and speedy referral to a stroke unit without the delay of visiting other specialists first.

Overall, in most patients, early examination for visual disturbances within 1 week is possible and acceptable as examinations do not take long and have no side effects, with feasibility and acceptability of vision screening being clearly indicated.^2^ The median for early vision screening was at 3 days post stroke admission.^2^ This is also important, as visual impairments are frequent following stroke. Further, earlier recognition can expedite treatment (patching, prisms) and rehabilitation (scanning training) efforts, influence other therapies (e.g. physiotherapy, speech and language therapy) chosen and thus benefit activities and quality of life.^1^ In addition, stroke survivors may not be aware of their visual disturbances such as in neglect or unable to report symptoms due to communication or cognitive problems.^12^ As there are no predictors of who will recover, the small percentage of patients with early recovery of their visual problems (within 3–4 weeks of onset) should not result in making all wait for a later assessment and treatment, potentially limiting adaptation, engagement in rehabilitation and activities of daily life for the majority with persistent visual impairment.^2^

When considering patient preferences and values, stroke survivors are quite likely to be willing to have early vision testing, whether screening or specialist assessment as it is not time consuming and there is no risk involved. Screening increases the likelihood to reveal vision problems, reduces the risk of misdiagnosis and delayed diagnosis compared to no screening. This has implications for rehabilitation but in some instances also for treatment and survival.

**Figure 2.**
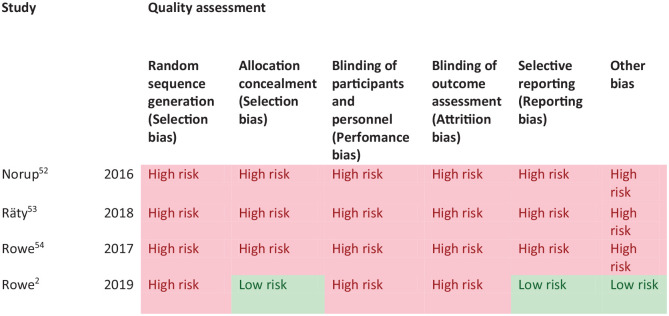
PICO 2 – Risk of bias assessment.

Most studies had a high risk of bias due primarily to being non-RCT design. Limitations included study heterogeneity, unblinded interpretation of test results and limited information on complete or missing data. Table 2.1 and Figure 2 show the GRADE assessment of vision screening tools. Meta analysis was not possible due to considerable heterogeneity across included studies trials with different interventions, outcome measures and timing of treatment post stroke.

**Table 2.1.** PICO 2 – For adults with visual problems due to stroke, does early assessment within 1 week of stroke admission, compared to later assessment, improve activities and quality of daily life? Summary of findings for PICO 2. Assessment of the time to visual assessment and length of stay. Participants: Stroke survivors. Settings: Variety (acute and out-patient). Intervention: Early vision screening or specialist visual assessment.

Test	Time to assessment	Length of stay		*N* participants/*N* with visual impairment	Risk of bias	GRADE
Vision screening^53^ Specialist visual assessment^2,52^ Survey^54^	Referral to eye teams: 81.8% referred at mean 8 days (SD 8.3) Time to vision screen: Mean 6.5 days (SD 24) Median 3 days (IQR 2) Time to full visual assessment: Mean of 13.4 days (SD 33.8) Median 4 days (IQR 7): 70% of stroke population Assessed: Within 4.5 h 20.8% Within 2 weeks of onset 35.5% Within 1 month of onset 55.5%			Four studies: One survey of clinicians Three studies of stroke populations 1889 (1300 vision screened)/1019	High	Low^a^ +ooo
Specialist visual assessment^2,52^		With visual impairment 49.9 (SD 68.3) 37.4 (SD 27.2) Pooled analysis: 49.69 days (SD 67.84)	Without visual impairment 13.5 (SD 45.9)	Two studies 1644 (1055 vision screened)/774	High	Moderate^b^ +++o

a Downgraded for risk of bias, indirectness and imprecision.

b Upgrade due to large effect size.

Stroke survivors with visual impairment had worse outcomes for activities of daily living and quality of life, indicated by significant reduction in Barthel Index and health-related questionnaire (EQ-5D-5L) scores (specifically issues with mobility and usual activities). However, no study evaluated change or improvement to activities and quality of life so there is no available evidence that early assessment within 1 week of stroke admission, compared to later assessment, improves activities and quality of daily life.


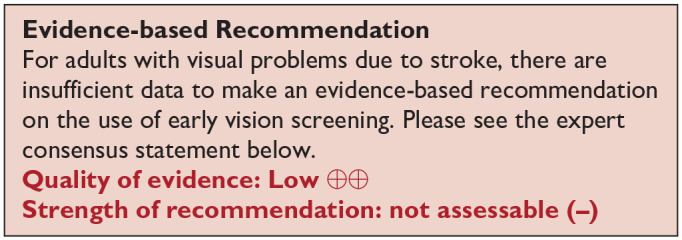



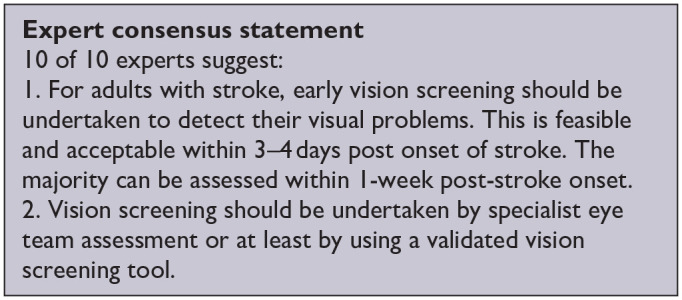


***PICO 3:* For adults with visual field loss due to stroke, does identification of visual field loss by vision screening or specialist eye team, compared to routine stroke screen, improve detection rate and activities/quality of life?**

### Analysis of current evidence

In this PICO, we consider whether in adults with visual field loss due to stroke, identification of visual field loss by vision screening or specialist eye team, compared to routine stroke screen, improves detection rate, activities of daily living and quality of life. As with the other PICOs, we considered any point in the stroke pathway. However, we were interested, particularly, in the acute setting as early vision screening is recommended in many international stroke best practice statements and clinical guidelines (e.g. ICSWP 2023, NICE 2023).^30,31^

We found no studies that directly compared visual field outcomes from vision screening or specialist eye team assessment compared to routine stroke screening

### Additional information

Overall, we found 19 studies (Supplemental Tables 7.1 and 7.2) that were relevant to the PICO topic but not completely aligned with the original question in that these studies reported visual symptoms related to visual field loss (but not objective measurements of visual field) or reported vision screening outcomes versus specialist eye team assessment (but not compared to routine stroke screening).^2,33,35,36,38,41,42,44,46,48,49,52,55–61^

**Figure 3.1.**
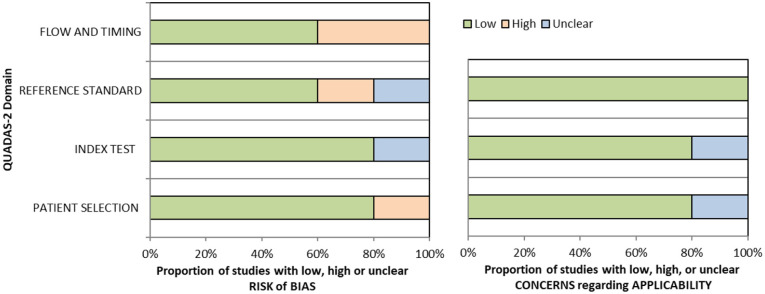
QUADAS domain for PICO 3.

**Figure 3.2.**
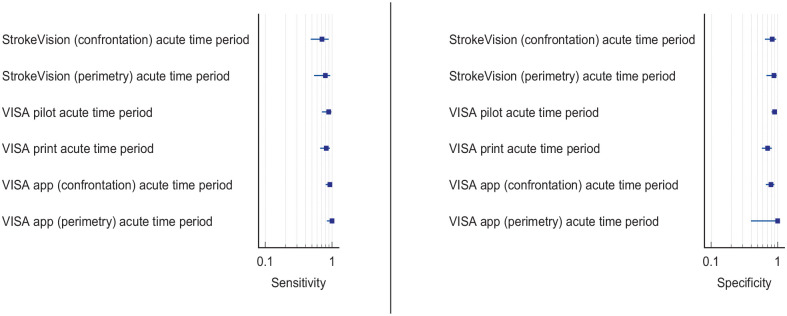
Sensitivity and specificity forest plots for PICO 3.^35,36,38^

For this PICO, we included those outcomes rated as critical by the writing group. We prioritised sensitivity, specificity and detection rates. We found four comparative studies evaluating visual assessment tools which aim to improve detection of visual field loss in stroke survivors with a median sample size of 101 (range 48–883; mean 249.6, SD 355.0).^35,36,38,55^ Median number of stroke survivors completing visual screening was 101 (range 45–883; mean 246.8, SD 356.5). The visual assessment tools included app-based vision screening tools: StrokeVision App,^36^ Vision Impairment Screening Assessment (VISA) tool (in print or as an app),^35,38^ and the Prehospital Ambulance Stroke Test (PreHAST).^55^ Across the four included studies, sensitivity ranged from 5.3 to 92.9%, with the PreHAST test showing a low sensitivity of 5.3%.^35,36,38,55^ This was distinct from the remaining post-admission vision screening tools which showed consistently high sensitivity for identification of visual field loss versus standard confrontation methods, ranging from 71.0% to 92.9% (average 82.8%), and high specificity, ranging from 70.9% to 89.7% (average 82.2%).^35,36,38^ The majority of studies had a low risk of bias, on QUADAS assessment. Table 3.1 and Figure 3.1 show the QUADAS assessment of diagnostic accuracy of visual field screening tools. Figure 3.2 shows forest plots of diagnostic accuracy.

**Table 3.1.** PICO 3 – For adults with visual field loss due to stroke, does identification of visual field loss by vision screening or specialist eye team, compared to routine stroke screen, improve detection rate and activities/quality of life? Summary of findings for PICO 3. Assessment of the diagnostic accuracy of vision screening for diagnosis of post-stroke visual field loss. Participants: Stroke survivors. Settings: Variety (pre-hospital, acute and out-patient). Intervention: Vision screening for visual field loss. Reference: Specialist visual assessment.

Test	Summary sensitivity Summary specificity	*N* participants/*N* with visual field loss	QUADAS-2
PreHAST hyper-acute time period^55^	0.053 0.981	One study 883/33	High^a^
StrokeVision acute time period^36^	Versus confrontation 0.71 (95%CI: 0.48–0.89) 0.83 (95%CI: 0.64–0.95)	One study 48/19 (45/19)	Medium^b^
	Versus perimetry 0.79 (95%CI: 0.54–0.94) 0.88 (95%CI: 0.68–0.97)	(43/19)	
VISA acute time period^35,38^	VISA pilot 0.8889 (95%CI: 0.7084–0.9765) 0.8974 (95%CI: 0.8079–0.9547)	Two studies (pilot/validation) and three groups (pilot/validation [print/app]) 317 (306 vision screened)/108 (105/27)	Medium^c^
	VISA print 0.8205 (95%CI: 0.6647–0.9246) 0.7097 (95%CI: 0.5805–0.818)	(101/39)	Low
	VISA app versus confrontation 0.9286 (95%CI: 0.8052–0.985) 0.7931 (95%CI: 0.6665–0.8883)	(100/42)	Low
	VISA app versus perimetry 1.0 (95%CI: 0.8389–1.0) 1.0 (95%CI: 0.3976–1.0)	(25/21 – included within app versus confrontation)	Low

PreHAST: Prehospital Ambulance Stroke Test; VISA: Visual Impairment Screening Assessment.

a Downgraded due to potential risk of bias on patient selection, index test, reference standard and flow of timing.

b Downgraded due to potential risk of bias on flow of timing and reference standard.

c Downgraded due to potential risk of bias on flow of timing.

The high sensitivity of these visual field screening tools suggests that they truly reflect a patient’s visual field status. Their administration is in general easy for patients, due to their simplicity and short duration. To improve the diagnostic accuracy of identifying visual impairment in hyperacute strokes, the VISA and StrokeVision screens are supported by an education package with detailed instructions and a video guide, which provide background information about stroke mimics and an understanding of the visual system.^35,36^ PreHAST and VISA are available free of charge for all clinical use and publicly funded research (https://sjtrem.biomedcentral.com/articles/10.1186/s13049-017-0377-x; www.vision-research.co.uk).

Regarding detection rates of visual field defects, we found 15 studies (two cross-sectional, 13 cohort) with a median sample size of 170 (range 22–1204; mean 302.8, SD 357.5 – see Supplemental Table 7.2).^2,33,41,42,44,46,48,49,52,56-61^ Detection rate of post-stroke visual field defects ranged from 18.1% to 79.0% (mean 40.4, SD 21.1, median 27.9), with variances mainly due to variation in the visual field indices, method of visual field testing used and population (any site of stroke vs occipital lobe only). In ten studies, the visual field was examined during vision specialist assessment,^2,41,44,46,48,52,58–61^ and in five studies detection of abnormal visual fields was based on vision screening.^33,42,49,56,57^ During vision specialist assessment, visual fields were usually examined by formal perimetry (Humphrey systems, Dublin, CA, USA) for automated static perimetry or Goldmann/Octopus, Haag Streit AG, Switzerland for kinetic perimetry), but confrontation assessment, tangent screen and Amsler grid were also used.^2,41,44,46,48,52,58–61^ During vision screening, visual fields were also assessed by formal or confrontation perimetry and iPad applications.^36,37,44,49^ Homonymous hemianopia was the most common visual field defect across studies.

Despite the clinical heterogeneity, studies included large numbers of patients and gave consistent findings across several settings. Although most of the above studies did not specifically aim to evaluate acceptability and feasibility issues, Quinn et al., Rowe et al. and Wijesundera et al. reported high acceptability of app-based visual field tests.^35–37^ Rowe et al. found that 79.8% of stroke admissions were able to undergo visual assessment within 1 week after stroke onset.^2^ Pooled analysis of the above studies showed that in 90% of cases visual assessment had been performed within the first month after the acute episode, with a median of 3 days. Test duration was reasonable and there were no associated risks with either vision screening or specialist assessment. Hence early visual field testing is recommended in stroke patients, as it is fast and acceptable by both patients and clinicians and has high detection accuracy.

The importance of prompt diagnosis of visual field abnormalities is that they may be the only presenting sign of posterior cerebral artery stroke. The primary striate cortex (area V1) in the occipital lobe processes only visual information.^62^ It is estimated that 90% of occipital lobe infarcts have only visual sequelae and 46% of stroke survivors with visual field loss report no visual symptoms.^1^ Consideration must also be given to whether visual field loss is monocular or binocular. Where suspicion is that of ocular stroke (central retinal artery occlusion), rapid referral for ophthalmic opinion is crucial. While fundus photography may show classic features of cherry red spot, very early fundus examination may not yet show signs of ischaemia. Here, optical coherence tomography is a vital screening assessment to detect inner retinal nerve layer hyper-reflectivity. Further, telemedicine opportunities can be explored to expedite ophthalmic consultation to confirm ocular stroke.

Delayed stroke diagnosis may have serious implications not only on visual rehabilitation and quality of life, but in certain cases also on an individual’s survival should the underlying diagnosis of stroke be missed. When considering patient preferences and values, it is likely that stroke survivors are willing to have visual field testing, particularly during screening, as this is not time consuming and aids identification of visual field loss (a desirable outcome) versus potential for missed diagnosis without screening (undesirable effect).

Vision screening versus routine stroke screening improves the detection rate of presence of visual field loss while specialist visual assessment further improves the accuracy of detection of visual impairment.

Based on the available evidence, the consensus expert opinion is, for adults with stroke, early vision screening should be undertaken to detect visual field loss. This is feasible and acceptable within 3–4 days post onset of stroke. The majority can be assessed within 1 week post onset. Visual field loss screening should be undertaken by specialist eye team assessment or at least by using a validated vision screening tool.

There is no evidence so far that identification of visual field loss by vision screening or specialist eye team, compared to routine stroke screen, improves activities/quality of life.


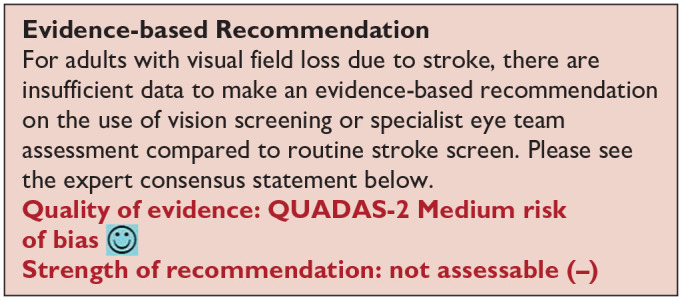



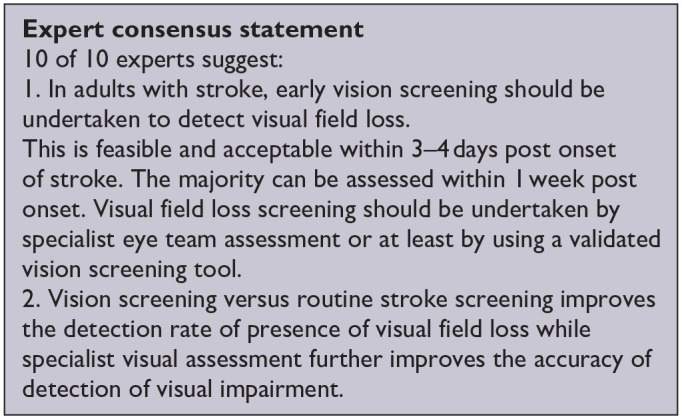


***PICO 4:* For adults with central vision impairment due to stroke, does identification of visual acuity loss by vision screening or specialist eye team, compared to routine stroke screen, improve detection rate and activities/quality of life?**

### Analysis of the current evidence

In this PICO, we consider the identification of loss of visual acuity, in particular, using vision screening tools or specialist eye team assessment to determine whether this improves detection rate of visual acuity loss, with impact on activities of daily living and quality of life for stroke survivors in comparison to identification of visual acuity loss by routine stroke screening. For the purposes of this PICO, we considered any point in the stroke pathway. However, we were interested, particularly, in the acute setting as early vision screening is recommended in many international stroke best practice statements and clinical guidelines (e.g. ICSWP 2023, NICE 2023).^30,31^

We found no studies that directly compared visual acuity outcomes from vision screening or specialist eye team assessment compared to routine stroke screening.

### Additional information

We found eight studies (Supplemental Tables 8.1 and 8.2) that were relevant to the PICO topic but not completely aligned with the original question in that these studies reported visual symptoms related to visual acuity (but not objective measurements of visual acuity) or reported vision screening outcomes versus specialist eye team assessment (but not compared to routine stroke screening which typically does not include an objective assessment of visual acuity.^1,33–35,38,43,44,51^

For this PICO, we included those outcomes rated as critical by the writing group. We prioritised sensitivity, specificity and detection rates. We found three comparative studies evaluating visual assessment tools which aimed to improve detection of visual field loss in stroke survivors with a median sample size of 108.5 (range 100–461; mean194.5, SD 177.8).^34,35,38^ Median number of stroke survivors completing visual screening was 100.5 (range 89–461; mean 187.7, SD 182.2). The visual assessment tools included the Vision Impairment Screening Assessment (VISA) tool (in print or as an app)^35,38^ and the Cerebral Vision Screening Questionnaire (CVSQ).^34^ CVSQ and VISA are available free of charge for all clinical use and publicly funded research (accessible from: www.uni-saarland.de/fileadmin/upload/lehrstuhl/kerkhoff/Materialien_für_Diagnostik_Therapie/CVSQ.pdf; www.vision-research.co.uk).

**Figure 4.1:**
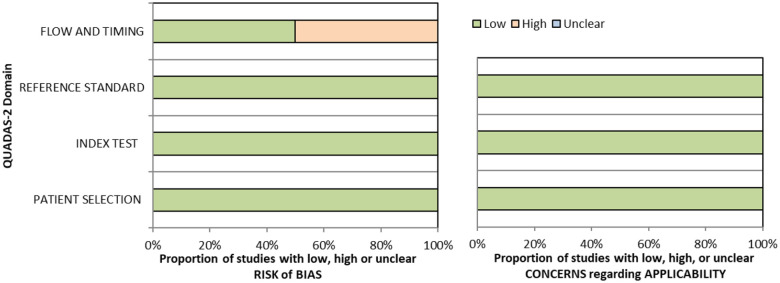
QUADAS domain for PICO 4.

**Figure 4.2.**
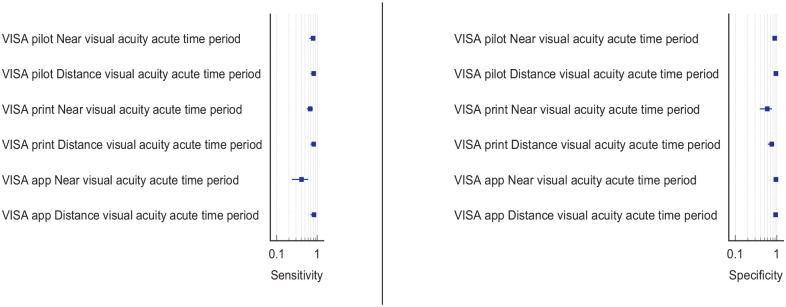
Sensitivity and specificity forest plots for PICO 4.^35,38^

Overall, sensitivity and specificity results were available for all of the above studies.^34,35,38^ Table 4.1 and Figure 4.1 show the QUADAS assessment of diagnostic accuracy of visual acuity testing. Figure 4.2 shows forest plots of diagnostic accuracy. CVSQ is a symptoms-based questionnaire.^34^ Sensitivity and specificity were 83.9% and 79.1% for reading problems, and 74.7% and 86.7% for blurred vision, respectively.^34^ VISA provides an objective measurement of visual acuity and averaged sensitivity and specificity for the different types of VISA were 62.3% and 81.0% for near visual acuity, and 82.7% and 87.9% for distance visual acuity.^35,38^

**Table 4.1.** PICO 4 – For adults with central vision impairment due to stroke, does identification of visual acuity loss by vision screening or specialist eye team, compared to routine stroke screen, improve detection rate and activities/quality of life? Summary of findings for PICO 4. Assessment of the diagnostic accuracy of vision screening for diagnosis of post-stroke visual acuity loss. Participants: Stroke survivors. Settings: Variety (pre-hospital, acute and out-patient). Intervention: Vision screening for visual acuity loss. Reference: Specialist visual assessment.

Test	Summary sensitivity Summary specificity	*N* participants/*N* with central visual impairment	QUADAS-2
CVSQ acute time period^34^	Reading problems 0.839 and blurred vision 0.791	One study Reading problems 461/217 Blurred vision 461/110	Medium^a^
	Reading problems 0.747 and blurred vision 0.867		
VISA acute time period^35,38^	VISA pilot – near visual acuity 0.7872 (95%CI: 0.6434–0.8930) 0.8889 (95%CI: 0.7844–0.9541)	Two studies (three groups: pilot/print/app) Near visual acuity 317 (309 vision screened)/150 Distance visual acuity 317 (316 vision screened)/132 (110/47)	Medium^b^
	VISA pilot – distance visual acuity 0.8261 (95%CI: 0.6858–0.9218) 0.9492 (95%CI: 0.8585–0.9894)	(115/46)	
	VISA print – near visual acuity 0.6761 (95%CI: 0.5545–0.7824) 0.5862 (95%CI: 0.3894–0.7648)	(100/71)	Low
	VISA print – distance visual acuity 0.8163 (95%CI: 0.6798–0.9124) 0.75 (95%CI: 0.6105–0.8597)	(101/49)	
	VISA app – near visual acuity 0.4062 (95%CI: 0.237–0.5936) 0.9552 (95%CI: 0.8747–0.9907)	(99/32)	Low
	VISA app – distance visual acuity 0.8378 (95%CI: 0.6799–0.9381) 0.9365 (95%CI: 0.8453–0.9824)	(100/37)	

CVSQ: Cerebral Vision Screening Questionnaire; VISA: Visual Impairment Screening Assessment.

a Downgraded due to potential risk of bias on flow of timing and reference standard.

b Downgraded due to potential risk of bias on flow of timing.

Six studies reported detection rates of visual acuity loss (two cross-sectional and four cohort: Supplemental Table 7.2) with a median sample size of 273 (range 23–1204; mean 455.2, SD 503.2).^1,33,34,43,44,51^ Overall, detection rate of visual acuity loss was a mean of 36.4% (SD 12.8; median 37.7%, range 20.9–54.0%). Variable detection rates were due to heterogeneous study designs, populations (e.g. formal stroke screening programmes vs referrals based on clinician suspicion; and general stroke cohorts vs specific stroke types or area of brain). Most studies had a low risk of bias, on QUADAS assessment. Objective measurements of visual acuity under good lighting conditions were important for obtaining consistency of testing and, therefore, more reliable measures.

In assessing the evidence for this PICO, there are some considerations to review. Impaired central vision primarily relates to a reduction in visual acuity which can be objectively measured by a range of acuity charts but can also be measured as a function of reading with text at specified font sizes. Impaired central vision may also be due to contrast sensitivity and/or colour vision impairment in a minority of cases. We included visual acuity loss as indicated by symptoms of blurred vision or objective assessment with acuity charts.

Here, we are particularly interested in detection of impaired visual acuity at an early stage post-stroke onset, whether by vision screening or specialist eye team assessment, to facilitate timely referral (and early management where indicated) in order to maximise improvement of activities of daily living and quality of life. Note, none of the studies reported specifically on impact of impaired/loss visual acuity to activities of daily living and/or quality of life. Of importance and relevance is the report of 58.5% of stroke survivors with impaired central vision being visually asymptomatic, that is, not reporting or unable to report visual symptoms.^1^

We did not include outcomes of false positives, false negatives, positive and negative predictive values and units of assessment. However, these are important considerations for vision screening and information on these outcomes are reported for some diagnostic accuracy studies. The acuity testing options were demonstrated to be feasible at various stages of stroke care. Acceptability by staff and patients was observed with screening possible in acute settings and often within 3 days of stroke onset.^2^

It is important to note for central visual impairment, that reduction or loss of visual acuity can be due to the stroke event, existence of prior ocular pathology/refractive error, or a combination. Co-existent ocular pathology is reported for about 30% with childhood strabismus/amblyopia accounting for a further 5.4%.^1^ Regardless of new onset or prior deficit, it is important to ascertain level of visual acuity in order to promote better visual function for safety of mobilisation, to be able to read, and to facilitate greater engagement with general rehabilitation.

When considering patient preferences and values, it is likely that stroke survivors are willing to have visual acuity testing, whether screening or specialist assessment, as this is not time consuming and aids identification of impaired visual acuity (a desirable outcome) versus potential for missed diagnosis without screening (undesirable effect). So far, no studies have been done to provide evidence that identification of visual acuity loss by vision screening or specialist eye team, in adults with central vision impairment due to stroke, improve detection rate and activities/quality of life compared to routine stroke screen.


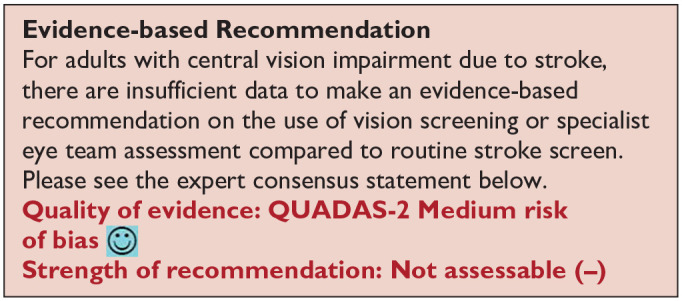



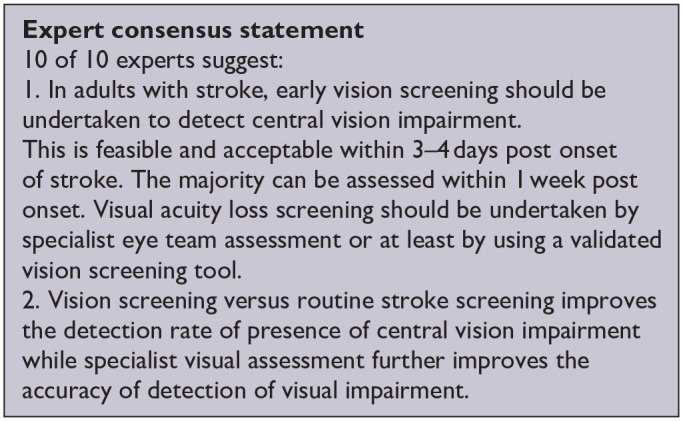


***PICO 5:* For adults with eye movement disorders due to stroke, does identification of strabismus and/or ocular motility deficit loss by vision screening or specialist eye team, compared to routine stroke screen, improve detection rate and activities/quality of life?**

### Analysis of current evidence

In this PICO, we consider the assessment of eye movement disorders either performed as part of a screen or specialist eye assessment, to determine if their use improves detection of visual problems due to stroke. For the purposes of this PICO, we considered any point in the stroke pathway. However, we were interested, particularly, in the acute setting as early vision screening is recommended in many international stroke best practice statements and clinical guidelines (e.g. ICSWP 2023, NICE 2023).^30,31^

We found no studies that directly compared eye movement disorder outcomes from vision screening or specialist eye team assessment compared to routine stroke screening.

### Additional information

We found ten studies (Supplemental Tables 9.1 and 9.2) that were relevant to the PICO topic but not completely aligned with the original question in that these studies reported vision screening outcomes and/or specialist eye team assessment (but not compared to routine stroke screening which typically would not include a full assessment of eye movements in all directions of gaze).^1,33,35,38,44,46,52,63–65^

For this PICO, we included those outcomes rated as critical by the writing group. We prioritised sensitivity, specificity and detection rates. We found three diagnostic accuracy studies that reported the sensitivity and specificity of the Visual Impairment Screening Assessment (VISA) tool^35,38^ and V-FAST screening tool,^33^ with a median sample size of 101 (range 43–116, mean 86.7, SD 38.5). Median number of stroke survivors completing visual screening was 89 (range 43–89; mean 77.7, SD 30.6).^33,35,38^ VISA and V-FAST are available free of charge for all clinical use and publicly funded research (www.vision-research.co.uk).

**Figure 5.1.**
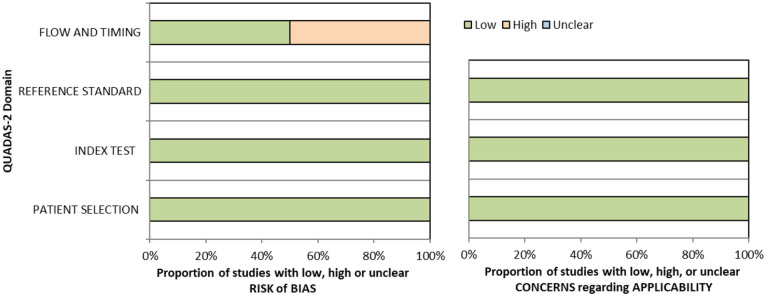
QUADAS domain for PICO 5.

**Figure 5.2.**
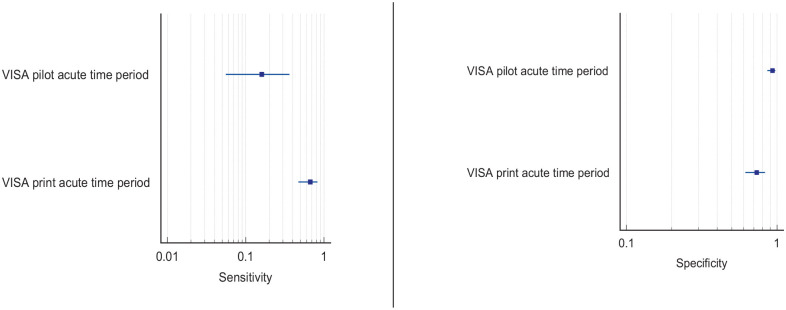
Sensitivity and specificity forest plots for PICO 5.^35,38^

Overall, sensitivity and specificity results were available for two of the above studies.^35,38^ Meta-analysis was not appropriate to give summary estimates of the sensitivity and specificity because of inclusion of just two (related) studies. Table 5.1 and Figure 5.1 show the QUADAS assessment of diagnostic accuracy of vision screening tools. Figure 5.2 shows forest plots of diagnostic accuracy. The lowest sensitivity was obtained during the pilot study of the initial VISA version (16%) but improved to 66.7% after refinement, during the validation study.^35,38^ Specificity for the pilot versus validated VISA tool was 93.4% and 73.2% respectively.^35,38^

**Table 5.1.** PICO 5 – For adults with eye movement disorders due to stroke, does identification of strabismus and/or ocular motility deficit loss by vision screening or specialist eye team, compared to routine stroke screen, improve detection rate and activities/quality of life? Summary of findings for PICO 5. Assessment of the diagnostic accuracy of vision screening for diagnosis of post-stroke eye movement disorders. Participants: Stroke survivors. Settings: Variety (pre-hospital, acute and out-patient). Intervention: Vision screening for eye movement disorders. Reference: Specialist visual assessment.

Test	Summary sensitivity Summary specificity	*N* participants/*N* with eye movement disorders	QUADAS-2
V-FAST pre-hospital^33^	V-FAST, 27.9% detection versus NIHSS item 3, 15.4% detection	One study 43/17	Medium^a^
VISA acute time period^35,38^	VISA pilot 0.16 (95%CI: 0.0554–0.3608) 0.9341 (95%CI: 0.862–0.9754)	Two studies (print versions only) 217 (190 vision screened)/55 (89/25)	Medium^a^
	VISA print 0.6667 (95%CI: 0.4719–0.8271) 0.7324 (95%CI: 0.6141–0.8306)	(101/30)	Low

V-FAST: Vision, Face, Arms, Speech, Time; VISA: Vision Impairment Screening Assessment.

a Downgraded due to potential risk of bias on flow of timing.

A total of eight papers reported detection rates of eye movement disorders (Supplemental Table 9.2), with a median sample size of 46.5 (range 22–1204; mean 292.0, SD 480.1).^1,33,44,46,52,63–65^ Three of these studies reported this across general stroke populations, with sample sizes ranging from 43 to 1204.^1,35,46^ Two studies reported findings of eye movement disorders as a result of a vision assessment following an initial suspicion of a visual impairment^44,52^ or within a specific stroke area.^64^ Two studies specifically recruited participants reporting dizziness, completing an assessment of eye movements.^63,65^ Overall, detection rate of eye movement disorders in stroke survivors, across variable time periods of pre-hospital to chronic stroke stages was a mean of 51.3% (SD 20.3; median 53.1%, range 27.2–78.0%).^1,33,44,46,52,63–65^ Variable detection rates were due to heterogeneous study designs, populations (e.g. formal stroke screening programmes versus referrals based on clinician suspicion, and general stroke cohorts versus specific stroke types or area of brain).

Screening for eye movement disorders was demonstrated to be feasible at various stages from prehospital to stroke unit acute care. Acceptability by staff and patients was observed with screening possible in acute settings and often within 3 days of stroke onset.^2^ However, best accuracy for detection of eye movement disorders was by specialist orthoptic assessment. Of importance and relevance is the report of 51.4% of stroke survivors with eye movement disorders being visually asymptomatic, that is, not reporting or unable to report visual symptoms.^1^ Hence, objective assessment is necessary for the detection of eye movement disorders.

When considering patient preferences and values, it is likely that stroke survivors are willing to have eye movement testing, whether screening or specialist assessment, as this is not time consuming and aids identification of eye movement disorders that often cause diplopia, blurred vision and oscillopsia (a desirable outcome) versus potential for missed diagnosis without screening (undesirable effect). Further, based on the wide range of eye movement defects identified, the identification of these would require the assessment of the different eye movement systems, i.e. smooth pursuits, saccades, optokinetic nystagmus, vestibulo-ocular reflex and vergence during assessment.


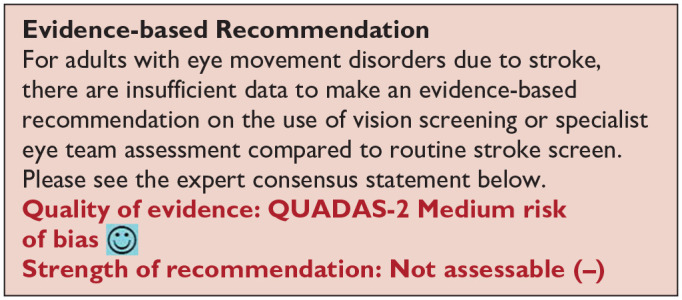



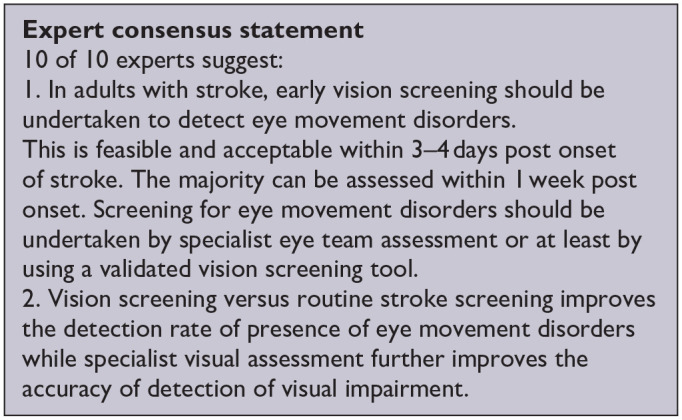


***PICO 6:* For adults with visual perceptual disorders due to stroke, does identification of visual perceptual disorders by screening proforma/tool or specialist team, compared to routine stroke screen, improve detection rate and activities/quality of life?**

### Analysis of current evidence

In this PICO, we consider the identification of visual perceptual disorders, distinct from visual neglect/inattention, in particular, using vision screening proformas/tools (e.g. checklists, questionnaires, toolkit of tests) or specialist eye team assessment and whether this improves detection rate of visual perceptual disorders, activities of daily living and quality of life for stroke survivors in comparison to identification of visual perceptual disorders by routine stroke screening. We defined visual perceptual disorders as higher order impairment of visual processing such that the individual could not recognise, or would have difficulty with recognition, by vision/sight. For the purposes of this PICO, we considered any point in the stroke pathway. However, we were interested, particularly, in the acute setting as early vision screening is recommended in many international stroke best practice statements and clinical guidelines (e.g. ICSWP 2023, NICE 2023).^30,31^

We found no studies that directly compared visual perception outcomes from vision screening or specialist eye team assessment compared to routine stroke screening.

### Additional information

A survey conducted of occupational therapist and orthoptists in 2019 across the United Kingdom and the Republic of Ireland revealed that assessment of visual perceptual disorders commonly used observations in function (93%) or asking about symptoms (94%).^66^ Only 18% reported using a specific test for screening of visual perceptual disorders other than visual inattention. Separate to this survey, we found seven studies (Supplemental Tables 10.1 and 10.2) that were relevant to the PICO topic but not completely aligned with the original question in that these studies detailed self-reported visual symptoms or vision screening outcomes from specialist eye team assessment (but not compared to routine stroke screening which typically would not include an objective evaluation of visual perception distinct from visual neglect).^1,34,44,46,67–69^

**Figure 6.1.**
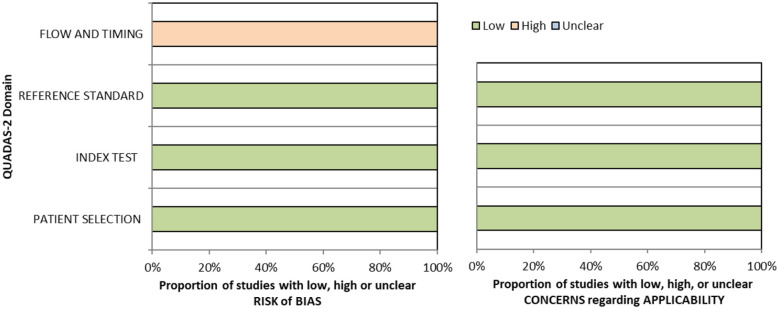
QUADAS domain for PICO 6.

For this PICO, we included outcomes that were rated as critical by the writing group, including sensitivity, specificity and detection rate. Only one study was found which reported the sensitivity and specificity of an assessment of visual perception; the Cerebral Vision Screening Questionnaire (CVSQ).^34^ All seven studies reported detection rate for visual perceptual disorders following stroke with a median sample size of 220 (range 50–1500; mean 503.3, SD 524.2).^1,34,44,46,67–69^ Median number of stroke survivors completing visual screening was 220 (range 50 to 1,204; mean 461, SD 434.7). Table 6.1 and Figure 6.1 show the QUADAS assessment. Meta analysis was not possible due to considerable heterogeneity across included studies with different interventions, outcome measures and timing of treatment post stroke.

**Table 6.1.** PICO 6 – For adults with visual perceptual disorders due to stroke, does identification of visual perceptual disorders by screening proforma/tool or specialist team, compared to routine stroke screen, improve detection rate and activities/quality of life? Summary of findings for PICO 6. Assessment of the diagnostic accuracy of vision screening for diagnosis of post-stroke visual perceptual disorders. Participants: Stroke survivors. Settings: Variety (pre-hospital, acute and out-patient). Intervention: Vision screening for visual perceptual disorders. Reference: Specialist visual assessment.

Test	Summary sensitivity Summary specificity	*N* participants/*N* with visual perceptual disorders	QUADAS-2
CVSQ acute time period^34^	Depth/reaching 0.864 and Dark vision 0.598 Depth/reaching 0.86 and Dark vision 0.909	One study Depth/reaching 461/217 Dark vision 461/110	Medium^a^

CVSQ: Cerebral Vision Screening Questionnaire.

a Downgraded due to potential risk of bias on flow of timing.

Five of these studies reported detection rates from evaluation of general stroke populations with an average detection rate of 11.2% for visual perceptual disorders.^1,34,44,46,69^ Two studies reported detection rates from specific sub populations of stroke survivors with an average detection rate of 56.9% for visual perceptual disorders.^67,68^

Considering only studies reporting the detection rate of overall visual perceptual disorders after stroke by visual specialist assessment (e.g. orthoptic or ophthalmological assessment),^1,2,29,44,46^ the pooled prevalence of visual perceptual disorders after stroke was 5.5% (95%CI: 4.8–6.2; Supplemental Table 10.2). Studies assessing the detection rate of visual perceptual disorders after stroke in general by other methods, such as the Motor-free Visual Perception Test 3rd edition (MVPT-3) or a novel experimental set-up of stimuli presentation, report higher detection rates: 50.8% at 3 weeks after returning home (35.9% at 6 months)^67^ and 63%,^68^ respectively. Other studies provide detection rates for specific types of perceptual disorders (such as motion, colour, shape, contrast, texture, location, orientation, etc.), but not for overall visual perceptual disorders. Since none of the studies reporting the detection rate of visual perceptual disorders after stroke do so by routine stroke screening (Supplemental Table 9.2), it is not known exactly how much vision specialist assessment, or the use of a specific screening proforma/tool, increases the detection rate of visual perceptual disorders after stroke.

Screening for visual perceptual disorders was demonstrated to be feasible at various stages from prehospital to stroke unit acute care. Acceptability by staff and patients was observed with screening possible in acute settings and often within 3 days of stroke onset.^2^ However, the best accuracy for detection of visual perceptual disorders was by specialist assessment using specific visual perception tests rather than questionnaires with symptom checklists. While it is important to ask about the potential presence/experience of visual perceptual disorders, stroke survivors may still not readily describe these, resulting in under-reporting of such issues and missed detection as a result. Here, use of tests specific to detection of visual perceptual disorders is likely to increase detection rate, which is important as about one-fifth of stroke survivors with visual perceptual disorders do not report visual symptoms.^1^

When considering patient preferences and values, it is likely that stroke survivors are willing to have vision perception screening as this is not time consuming and aids identification of disorders (a desirable outcome) versus potential for missed diagnosis without screening (undesirable effect). Early identification is important as visual perceptual disorders can be frightening (e.g. formed visual hallucinations) and can cause disturbing and disabling visual symptoms such as not being able to recognise faces of family and friends, or familiar objects. Reassurance can be critical to stroke survivors and their carers. There were no studies that provided any data whether screening by proforma/tool or specialist team, compared to routine stroke screen, improves activities or quality of life in adults with visual perceptual disorders due to stroke.


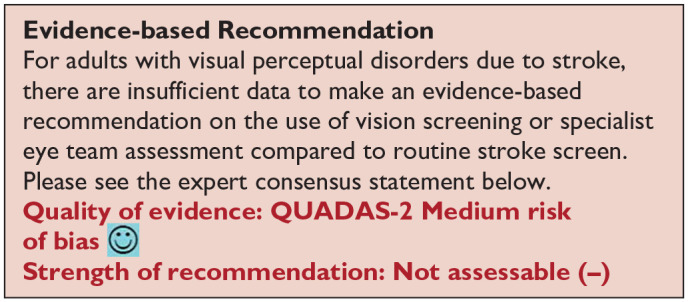



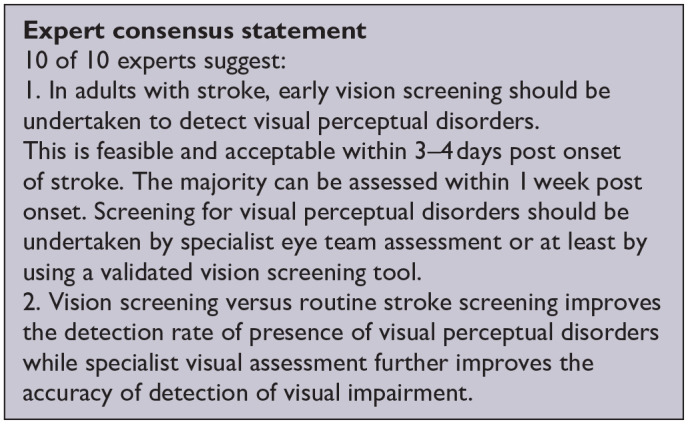


***PICO 7:* For adults with visual neglect due to stroke, does identification of visual neglect by screening proforma/tool or specialist team, compared to routine stroke screen, improve detection rate and activities/quality of life?**

### Analysis of current evidence

In this PICO, we consider the identification of visual neglect/inattention, in particular, using vision screening tools or specialist eye team assessment and whether this improves detection rate of visual neglect, and impact to activities of daily living and quality of life for stroke survivors in comparison to identification of visual neglect by routine stroke screening. We acknowledge the heterogeneity of neglect itself (rather than just the outcome measures), such as egocentric versus allocentric, personal, peri-personal versus extra-personal and so on. We sought to identify visual neglect specifically, regardless of its sub-type.

For the purposes of this PICO, we considered any point in the stroke pathway. However, we were interested, particularly, in the acute setting as early vision screening is recommended in many international stroke best practice statements and clinical guidelines (ICSWP 2023, NICE 2023).^30,31^

**Figure 7.1.**
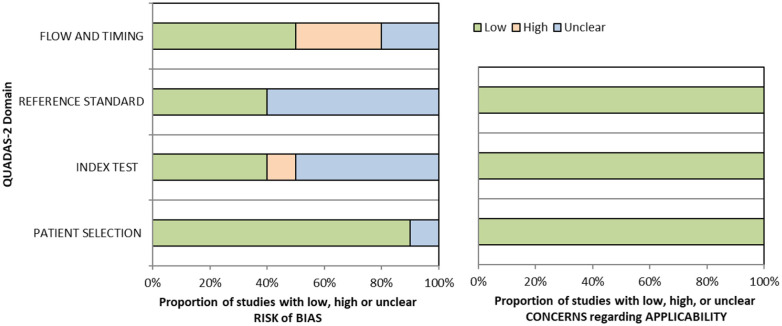
QUADAS domain for PICO 7.

**Figure 7.2.**
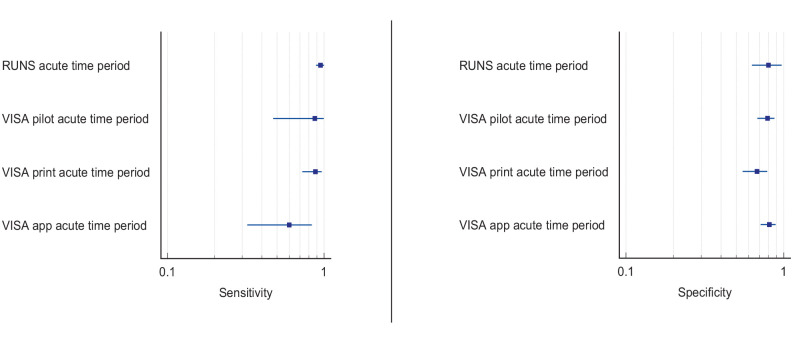
Sensitivity and specificity forest plots for PICO 7.^35,38,76^

For this PICO, we included those outcomes rated as critical by the working group: sensitivity, specificity and detection rate. We found four studies (Supplemental Table 11.1) reporting sensitivity and specificity of visual neglect assessment versus routine stroke screening, with a median sample size of 125.5 (range 67 to 428; mean 186.5, SD 163.6).^70–73^ Table 7.1 and Figure 7.1 show the QUADAS assessment of diagnostic accuracy of vision screening tools. Figure 7.2 shows forest plots of diagnostic accuracy.

**Table 7.1.** PICO 7 – For adults with visual neglect due to stroke, does identification of visual neglect by screening proforma/tool or specialist team, compared to routine stroke screen, improve detection rate and activities/quality of life? Summary of findings for PICO 7. Assessment of the diagnostic accuracy of vision screening for diagnosis of visual neglect. A Participants: Stroke survivors. Settings: Variety (pre-hospital, acute and out-patient). Intervention: Vision screening or specialist visual assessment. Reference standard: Routine stroke screen for visual neglect.

Test	Summary sensitivity Summary specificity	*N* participants/*N* with visual neglect	QUADAS-2
Mobility assessment course acute time period^70,71^	Grech: 0.742 0.694	Two studies 180/68 (67/31)	Medium^a^
	TenBrink: 0.828 0.905	(113/37)	Medium^b^
Driving performance acute time period^73^	0.52 0.943	One study 100/47	High^c^

OCS: Oxford Cognitive Screen.

a Downgraded due to potential risk of bias on index test and reference standard.

b Downgraded due to potential risk of bias on flow of timing and index test.

c Downgraded due to potential risk of bias on flow of timing, index test and reference standard.

**Table:** B Participants: Stroke survivors. Settings: Variety (pre-hospital, acute and out-patient). Intervention: Vision screening. Reference standard: Specialist visual or stroke assessment for visual neglect.

Test	Summary sensitivity Summary specificity	*N* participants/*N* with visual impairment	QUADAS-2
NIHSS acute time period versus OCS^72^	0.912 0.316	One study 428/199	Medium^a^
Video-oculography versus Catherine Bergego scale subacute time period^74^	Mean gaze position: 0.85 0.944	One study 78/60	High^b^
	Early orientation: 0.833 0.611		
Stimulus-driven attention test versus Catherine Bergego scale subacute time period^75^	0.6513 0.9475	One study 44/31	High^c^
RUNS test versus BEN test acute time period^76^	0.95 (95%CI: 0.89–1.0) 0.80 (95%CI: 0.63–0.97)	One study 75/51	Medium^d^
VISA versus specialist visual assessment acute time period^35,38^	VISA pilot 0.875 (95%CI: 0.4735–0.9968) 0.7895 (95%CI: 0.6808–0.8746)	Two studies (three groups) 317 (283 vision screened)/57 (84/8)	Medium^e^
	VISA print 0.8824 (95%CI: 0.7255–0.967) 0.6769 (95%CI: 0.5495–0.7877)	(99/34)	Low
	VISA app 0.60 (95%CI: 0.3229–0.8366) 0.8118 (95%CI: 0.7124–0.8884)	(100/15)	Low

BEN: Batterie d’Évaluation de la Négligence spatiale unilatérale; RUNS: Rapid Unilateral Neglect screening; VISA: Vision Impairment Screening Assessment.

a Downgraded due to potential risk of bias on index test and reference standard.

b Downgraded due to potential risk of bias on patient selection, index test, reference standard and flow of timing.

c Downgraded due to potential risk of bias on index test, reference standard and flow of timing.

d Downgraded due to potential risk of bias on reference standard and flow of timing.

e Downgraded due to potential risk of bias on flow of timing.

Overall, for these four studies, sensitivity values were consistently high (83–91%) for the largest sample studies but with trade-off for specificity (32–94%).^70–73^ Some studies had very strict inclusion and/or exclusion criteria, such as only right hemispheric strokes, and different options for assessment of visual neglect (e.g. Oxford Cognitive Screen (OCS) with lowest sensitivity of 52% and driving performance with lowest specificity of 32%).^70–73^ Accordingly, high sensitivity may only apply to that very defined population, not globally.

### Additional information

We found a further five studies reporting sensitivity and specificity of visual neglect assessment but against alternative, non-routine and/or specialist vision assessment.^35,38,74–76^ Median sample size for these studies was 89 (range 44–116; mean 85.6, SD 25.6). Mean number of stroke survivors completing visual screening was 81.2, SD 21.2; median 83.5, range 44–101. Again, across these studies, sensitivity and specificity values were consistently moderate to high (sensitivity 60–95%; specificity 61–94%) despite a range of different outcome measures.^35,38,74–76^

With regard to detection rates for visual neglect, we found 13 studies with a median sample size of stroke survivors completing visual assessments of 107 (range 22–1204; mean 303.9, SD 376.8; Supplemental Table 11.2).^1,35,44,45,52,71,72,74,76–80^ Overall, for the above studies, mean detection rate was 40%, SD 23.5 (median 33.2%, range 11.7–86.2%). Variances were due to differences in testing, stroke population recruited (e.g. right vs left hemisphere stroke) and acute versus long-term assessment.

Routine stroke screen typically comprised checklists and stroke scale scores such as NIHSS. Vision screening often employed mainly pen and paper tasks and/or Catherine Bergego scale in comparison to specific visual neglect screening or specific assessments such as the mobility assessment course (MAC), OCS, video-oculography, rapid unilateral neglect screening and VISA (Vision Impairment Screening Assessment). It was concluded that, while the MAC and video-oculography are valid alternatives for assessing neglect, regarding diagnostic accuracy, there is currently not enough evidence to suggest that these are a big step forward or practical in comparison to the accuracy of conventional pen and paper tests in acute diagnostic settings.

When considering patient preferences and values, it is likely that stroke survivors are willing to have visual neglect screening as this is not time consuming and aids identification of this condition (a desirable outcome) versus potential for missed diagnosis without screening (undesirable effect). Significant numbers of stroke survivors with visual neglect do not report symptoms; 58.4% reported no visual symptoms specifically.^1^ Much of this lack of reporting may be due to anosognosia but may also relate to communication difficulties or confusion of visual symptoms. Thus, early identification is important as visual neglect poses considerable issues for functional independence.^81^

Based on the available evidence, we recommend early vision screening for visual neglect using a battery of tests.^82^ Single tests or checklists such as the NIHSS observational measure lack sensitivity in identifying post-stroke unilateral neglect.^8,72^ Conversely, screening tool and specialist assessment consistently detect more visual neglect than routine screening. Sensitivity and specificity values improve with more detailed screening proformas or specialist assessment (using a combination of pen and paper tools). Those affected might demonstrate neglect behaviour in everyday settings despite showing no signs of neglect during common neglect tasks. Increasing task demands under more ecologically valid scenarios has become an important method of increasing test sensitivity. Acceptability by staff and patients has been observed with screening possible acutely and often within 3 days of stroke onset. We found no studies investigating if identification of visual neglect by screening proforma/tool in adults with visual neglect due to stroke improves activities/quality of life compared to routine stroke screen.


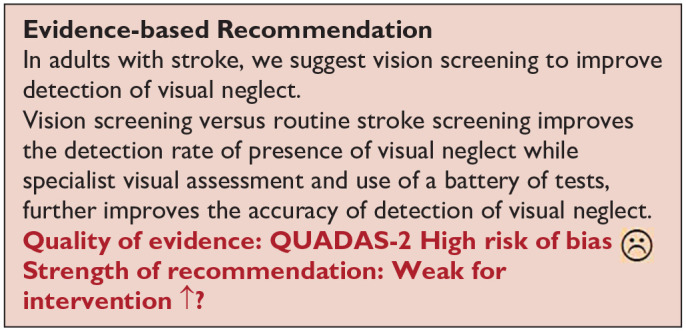


### TREATMENT

***PICO 8:* For adults with homonymous visual field loss due to stroke, does compensatory, substitute or restitutive intervention, compared to no intervention, improve activities and quality of daily life?**

### Analysis of the current evidence

In this PICO, we consider whether compensatory, substitute or restitutive interventions can improve activities and quality of daily life in stroke patients with homonymous visual field defects. For the purpose of the present guidelines, we define compensatory, substitutive and restitutive interventions as treatment options to improve adaptation to the impairment (compensatory, e.g. visual scanning training), to improve the visual impairment using a device or optical aid (substitutive, e.g. occlusion) and to restore visual field (restitution, e.g. visual perceptual training). We considered any point in the stroke pathway.

**Figure 8.**
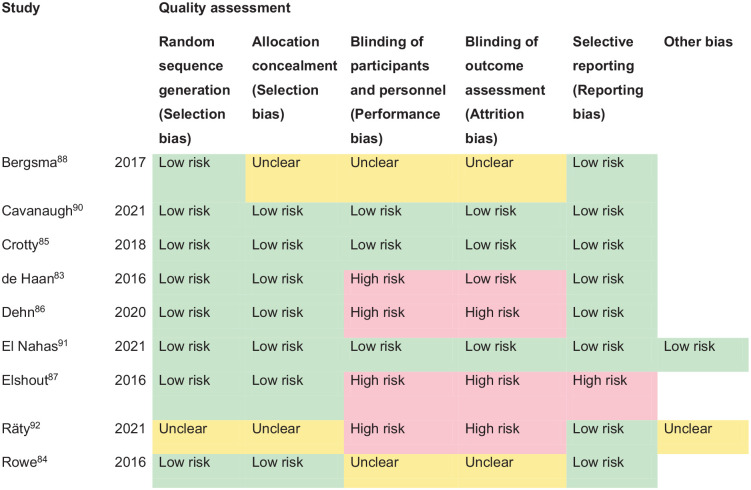
PICO 8 – Risk of bias assessment.

For this PICO, we included outcomes that were rated as critical by the writing group. These included change in visual field, activities of daily living (ADL), quality of life (QoL), driving, reading speed and accuracy and falls. We found nine randomised controlled trials (RCTs) (Supplemental Table 12) that compared interventions for visual field loss post-stroke, with a mean sample size of 44.6 (SD 19.5; median 45, range 24–87).^83–92^ Four trials evaluated compensatory interventions of visual scanning/search training.^83–86^ Measurement of ADL was not consistent across these studies. Change in ADL (measured by the Cerebral Visual Disorders questionnaire) was significant for use of the intervention in one trial but not another (measured by functional mobility and extended ADL). Change in QoL when measured by the Visual Function Questionnaire (VFQ-25) was significant for three trials but non-significant when measured by a health-related QoL (EQ-5D) questionnaire or using the Beck depression inventory measure. Rowe et al. also reported a substitutive intervention (monocular prism segments) as one of the three arms of their RCT.^84^ Use of prisms in this trial showed non-significant changes for ADL, QoL and reading accuracy/speed, and a 69% adverse event rate. Five trials evaluated restitutive interventions of visual perception/discrimination training,^87,88,90^ repetitive transcranial magnetic stimulation^91^ or transcranial alternating current and direct-current stimulation.^92^ Outcome measures were variable across all five trials with two reporting significant change in mean deviation of visual fields. However, these changes were of limited clinical significance with changes reported up to 3 dB. The VFQ-25 QoL results were significant for one trial but not another, with the same found for reading performance. Table 8.1 and Figure 8 show the GRADE assessment of interventions for homonymous visual field loss. Meta analysis was not possible due to considerable heterogeneity across included trials with different interventions, outcome measures and timing of treatment post stroke. The majority of studies had a high risk of bias. Limitations included study heterogeneity, unblinded interpretation of test results and limited information on complete or missing data.

**Table 8.1.** PICO 8 – For adults with homonymous visual field loss due to stroke, does compensatory, substitute or restitutive intervention, compared to no intervention, improve activities and quality of daily life? Summary of findings for PICO 8. Assessment of the interventions for stroke-related visual field loss. A. Participants: Stroke survivors with visual field loss. Settings: Acute to chronic. Intervention: Compensatory visual scanning/search training. Reference standard: Control, sham or standard care.

Outcome	*N* participants	Effect sham/standard care	Effect intervention	Significance between groups	Quality of evidence (GRADE)
**Change in visual field** Visual field border^83^	De Haan: 26 intervention 23 waiting list control 25 healthy controls	Change from baseline to 3 months Nil for 15 of 46 eyes	Change from baseline to 3 months Nil for 27 of 52 eyes	NS: *p* = 0.207	+ooo Very low^a^
Visual field area^84^	Rowe: 25 intervention (compensatory) 24 intervention (substitutive) 22 standard care	Change from baseline to 6 months 0.035 (SD 0.15), NS	Change from baseline to 6 months Compensatory 0.08 (SD 0.15), NS	NS: *p* = 0.555	
**Change in activities of daily living** Cerebral Visual Disorders questionnaire^83^	De Haan: 26 intervention 23 waiting list control 25 healthy controls	Change from baseline to 3 months 0.07, NS	Change from baseline to 3 months 0.55, *p* < 0.05	S: *p* < 0.05	++oo Low^b^
Functional mobility Extended ADL^84^	Rowe: 25 intervention (compensatory) 24 intervention (substitutive) 22 standard care	Change from baseline to 6 months Functional mobility Extended ADL	Change from baseline to 6 months Functional mobility Extended ADL	NS: *p* = 0.36 NS: *p* = 0.93	
**Change in quality of life** NEI VFQ-25^85^	Crotty: 10 intervention 10 standard care	Change from baseline to 3 months Mean −11.0 (SD 8.3)	Change from baseline to 3 months Mean −2.6 (SD 17.3)	S: *p* = 0.028	+++o Moderate^c^
NEI VFQ-25^83^	De Haan: 26 intervention 23 waiting list control 25 healthy controls	Change from baseline to 3 months 0.17, NS	Change from baseline to 3 months 0.65, *p* < 0.05	S: *p* < 0.001	
Health-related (EQ5D VAS)^84^ Vision-related (NEI VFQ-25)^84^	Rowe: 25 intervention (compensatory) 24 intervention (substitutive) 22 standard care	Change from baseline to 6 months Health-related Visual-related: parameter estimate 0.0	Change from baseline to 6 months Health-related VFQ: 10.417 (SD 4.37), *p* = 0.02	NS: *p* = 0.60	
Beck Depression Inventory-II^86^	Dehn: 20 intervention 20 healthy controls	Change from baseline to 2 weeks Mean 1.0, *p* = 0.021	Change from baseline to 2 weeks Mean 2.3, *p* = 0.002	NS: *p* = 0.182	
**Change in reading speed** Visual Skills for Reading^85^	Crotty: 10 intervention 10 standard care	Change from baseline to 3 months Mean −4.3 (SD 22.2), NS	Change from baseline to 3 months Mean 1.7 (SD 24.9), NS	NS: *p* = 0.448	+ooo Very low^d^
Radner (average effect size)^83^	De Haan: 26 intervention 23 waiting list control	Change from baseline to 3 months 0.03, NS	Change from baseline to 3 months 0.23, NS	NS: *p* = 0.36	
Radner^84^	Rowe: 25 intervention (compensatory) 24 intervention (substitutive) 22 standard care	Change from baseline to 6 months	Change from baseline to 6 months	NS: *p* > 0.1	
**Change in reading accuracy** Radner^83^	De Haan: 26 intervention 23 waiting list control 25 healthy controls	Change from baseline to 3 months 0.44, *p* < 0.05	Change from baseline to 3 months 0.06, NS	NS: *p* > 0.1	+ooo Very low^b^
Radner^84^	Rowe: 25 intervention (compensatory) 24 intervention (substitutive) 22 standard	Change from baseline to 6 months	Change from baseline to 6 months	NS: *p* > 0.05	
**Adverse events** ^83−85^	Crotty: 10 intervention 10 standard care		No serious adverse events noted		+++o Moderate^e^
	De Haan: 26 intervention 23 waiting list control		No serious adverse events noted		
	Rowe: 25 intervention (compensatory) 24 intervention (substitutive) 22 standard care		Compensatory: 6.7% reports of headaches and fatigue using scanning training		
**Change in driving**	Not reported				
**Change in falls**	Not reported				

ADL: Activities of Daily Living; EQ5D VAS: EQ5D Visual Analogue Scale; NEI VFQ-25: National Eye Institute Visual Function Questionnaire.

a Downgraded due to risk of bias, limited precision, inconsistency and potential publication bias.

b Downgraded due to risk of bias, limited precision and potential publication bias.

c Downgraded due to potential publication bias.

d Downgraded due to high risk of bias, indirectness, limited prevision and potential for publication bias.

e Downgraded due to high risk of bias.

**Table:** B Participants: Stroke survivors with visual field loss. Settings: Acute to chronic. Intervention: Substitutive (Fresnel prisms). Reference standard: Control, sham or standard care.

Outcome	N participants	Effect sham/standard care	Effect intervention	Significance between groups	Quality of evidence (GRADE)
**Change in visual field** Visual field area^84^	Rowe: 25 intervention (compensatory) 24 intervention (substitutive) 22 standard care	Change from baseline to 6 months 0.035 (SD 0.15), NS	Change from baseline to 6 months Prisms 0.052 (SD 0.139), NS	NS: *p* = 0.555	++oo Low^a^
**Change in activities of daily living** ^84^		Change from baseline to 6 months Functional mobility Extended ADL	Change from baseline to 6 months Functional mobility Extended ADL	NS: *p* = 0.36 NS: *p* = 0.93	++oo Low^a^
**Change in quality of life** Health-related (EQ5D VAS) Vision-related (NEI VFQ-25)^84^		Change from baseline to 6 months Health-related Visual-related: parameter estimate *p* = 0.05	Change from baseline to 6 months Health-related Prisms: 2.867 (SD 4.49), *p* = 0.5256	NS: *p* = 0.60	++oo Low^a^
**Change in reading speed** Radner^84^		Change from baseline to 6 months	Change from baseline to 6 months	NS: *p* > 0.05	++oo Low^a^
**Change in reading accuracy** Radner^84^		Change from baseline to 6 months	Change from baseline to 6 months	NS: *p* > 0.05	++oo Low^a^
**Adverse events** ^84^			Prisms: 69.2% reports of headaches, diplopia, navigation difficulties, dizziness, optical glare and visual confusion using monocular prism		++oo Low^a^
**Change in driving**	Not reported				
**Change in falls**	Not reported				

ADL: Activities of Daily Living; EQ5D VAS: EQ5D Visual Analogue Scale; NEI VFQ-25: National Eye Institute Visual Function Questionnaire.

a Downgraded due to potential risk of bias and limited precision.

**Table:** C Participants: Stroke survivors with visual field loss. Settings: Acute to chronic. Intervention: Restitutive training. Reference standard: Control, sham or standard care.

Outcome	*N* participants	Effect sham/standard care	Effect intervention	Significance between groups	Quality of evidence (GRADE)
**Change in visual field** Increase in cortical surface gain (mm)^88^	Bergsma: 17 intervention 7 controls Cross-over trial	Change from baseline to 16 weeks 3.26 ± 1.63 mm *p* = 0.023	Change from baseline to 16 weeks 4.76 ± 2.35 mm *p* = 0.003	S: *p* = 0.010	+ooo Very low^a^
Mean deviation^90^	Cavanagh: 23 intervention 23 sham	Change from baseline to 6 months Right eye: Mean 0.12 dB 95%CI: −0.38, 0.62 Left eye: Mean 0.10dB 95%CI: −0.52, 0.12	Change from baseline to 6 months Right eye: Mean 0.58 dB 95%CI: 0.07, 1.08 Left eye: Mean 0.84dB 95%CI: 0.22, 1.47	NS: *p* = 0.10	
Visual field index^91^	El Nahas: 21 intervention 11 sham	Change from baseline to 1 month Right eye: Mean 0.07 dB (SD 1.2) Left eye: Mean 0.071 dB (SD 0.9) Right eye: Mean −1.55 (SD 3.2) Left eye: Mean −2.4 (SD 4.4)	Change from baseline to 1 month Right eye: Mean 3.37 dB (SD 5) Left eye: Mean 2.51 dB (SD 3) Right eye: Mean 14.82 (SD 16) Left eye: Mean 8.41 (SD 13.9)	S: *p* = 0.04 S: *p* = 0.003 S: *p* = 0.001 S: *p* = 0.008	
Increase in cortical surface gain (mm)^87^ Mean sensitivity^87^	Elshout: 27 intervention Cross-over trial	Change from baseline to 8 weeks Mean 0.03 (SEM 0.67) Mean 0.26 (SEM 0.17), *p* = 0.129	Change from baseline to 8 weeks Mean 4.34 (SEM 1.03) Mean 0.68 (SEM 0.19), *p* = 0.001	S: *p* = 0.002	
Mean sensitivity^92^	Raty: Experiment 1: 8 intervention, 8 intervention, 8 sham Experiment 2: 9 intervention, 9 sham Experiment 3: 7 intervention, 7 sham	Change from baseline to 8 weeks Experiment 1: ipsilesion Median 0.3 (IQR −0.05, −0.7), *p* = 0.039 contralesion Median 0.3 (IQR −0.3, −0.6), *p* = 0.021 Experiment 2: ipsilesion Median 0.3 (IQR −0.6, −1.2), *p* = 0.175 contralesion Median 0.4 (IQR −0.7, −0.7), *p* = 0.368 Experiment 3: ipsilesion Median 0.5 (IQR −1.1, −0.9), *p* = 0.368 contralesion Median −0.5 (IQR −1.7–0.3), *p* = 0.317	Change from baseline to 8 weeks Experiment 1: ipsilesion I × 1 Median 0.07 (IQR −0.6–0.2), *p* = 0.607 I × 2 Median 0.5 (IQR −0.5–1.2), *p* = 0.417 contralesion I × 1 Median −0.3 (IQR −1.2–0.7), *p* = 0.417 I × 2 Median 0.7 (IQR −0.3–1.11), *p* = 0.135 Experiment 2: ipsilesion Median 0.3 (IQR −0.5–0.8), *p* = 0.641 contralesion Median 0.5 (IQR − 1.2–1.0), *p* =641 Experiment 3: ipsilesion Median 0.1 (IQR −0.7–2.0), *p* = 0.459 contralesion Median 1.3 (IQR 0.5–3.3), *p* = 0.163	NS: *p* = 0.454 NS: *p* = 0.213 NS: *p* = 0.845 NS: *p* = 0.949 NS: *p* = 0.710 NS: *p* = 0.017	
**Change in activities of daily living** Goal Attainment Scale^88^	Bergsma: 17 intervention 7 controls Cross-over trial	Change from baseline to 16 weeks Not reported	Change from baseline to 16 weeks 8.3 ± 1.3	S: *p* < 0.005	++oo Low^b^
**Change in quality of life** NEI VFQ-25^90^	Cavanagh:23 intervention 23 sham	Change from baseline to 6 months Mean 5.5 95%CI: 1.4, 9.5	Change from baseline to 6 months Mean 0.2 95%CI: −4.2, 4.7	NS: *p* > 0.5	+++o Moderate^c^
NEI VFQ-25^91^	El Nahas: 21 intervention 11 sham	Change from baseline to 6 months Mean −7.9 (SD 18.5)	Change from baseline to 6 months Mean 70.6 (SD 126)	S: *p* = 0.04	
**Change in reading speed** Arial 15-point text^88^	Bergsma: 17 intervention 7 controls Cross-over trial	Change from baseline to 16 weeks 0.034 + 0.001	Change from baseline to 16 weeks 2 + 0.022	S: *p* < 0.05	+ooo Very low^d^
Arial 15-point text^87^	Elshout: 27 intervention Cross-over trial	Change from baseline to 8 weeks Mean 7.70% (SEM 2.75), *p* = 0.011	Change from baseline to 8 weeks Mean 11.26% (SEM 3.34), *p* = 0.002	NS: *p* = 0.551	
iResT^92^	Raty: Experiment 1: 8 intervention, 8 intervention, 8 sham Experiment 2: 9 intervention, 9 sham Experiment 3: 7 intervention, 7 sham	Change from baseline to 8 weeks Experiment 1: Mean 6.5 (−4.5–16.0), *p* = 0.284 Experiment 2: Mean 2.2 (−7.0–11.6), *p* = 0.641 Experiment 3: Mean 0.5 (−10.0–28.5), *p* = 0.459	Change from baseline to 8 weeks Experiment 1: I × 1: Mean 6.0 (−5.3–22.3), *p* = 0.792 I × 2: Mean 14.5 (5.3–21.0), *p* = 0.005 Experiment 2: Mean 4.6 (0.5–14.8), *p* = 0.097 Experiment 3: Mean 5.5 (−6.0–7.0), *p* = 0.513	NS: *p* = 0.382 NS: *p* = 0.328 NS: *p* = 1.0	
**Adverse events** ^92^	Raty Experiment 1: 8 intervention, 8 intervention, 8 sham Experiment 2: 9 intervention, 9 sham Experiment 3: 7 intervention, 7 sham		No serious adverse events noted. Mild adverse events included mild skin irritation, metallic taste, phosphene-like visual phenomenon and fatigue		+++o Moderate^e^
**Change in driving**	Not reported				
**Change in falls**	Not reported				

ADL: Activities of Daily Living; iResT: International Reading Speed Texts; NEI VFQ-25; National Eye Institute Visual Function Questionnaire.

a Downgraded due to risk of bias, limited precision, inconsistency and potential publication bias.

b Downgraded due to risk of bias, limited precision and potential publication bias.

c Downgraded due to potential publication bias.

d Downgraded due to high risk of bias, indirectness, limited prevision and potential for publication bias.

e Downgraded due to high risk of bias.

### Additional information

We found a further three studies (cohort/cross-sectional) reporting interventions for homonymous visual field loss due to stroke (Supplemental Table 12) with a median sample size of 294 (range 22–426; mean 247.7, SD 205.5).^89,93,94^

Overall, a range of interventions have been evaluated in relation to compensatory, substitutive and/or restitutive treatment options. Compensatory approaches to rehabilitation of vision loss as a result of stroke are aimed at improving the efficacy of eye movements to scan and search more effectively into the affected/blind hemifield to better detect objects and explore that spatial environment. Substitutive approaches use, for example, prisms to enable patients to become aware of otherwise unseen stimuli through prismatic image displacement and overlap into their sighted field. Restitutive theories presume an enhanced plasticity potential of the visual pathway with potential for improvement in visual field area and/or sensitivity.

The current evidence suggests that compensatory interventions (specifically visual scanning/search training) have a positive and significant effect on the activities of daily living in patients with visual field loss after stroke, in line with previous systematic reviews on this subject.^18,19^ Scanning/search training can start from as early as day 1 post stroke onset but is generally commenced at a time point when the stroke survivor has capacity and capability to do the training. There are a variety of free and paid-access training options available internationally.^95^ Visual scanning/search training adds significantly to the compensatory mechanisms that underpin adaptation to visual field loss, both during training in the early and the chronic stroke phase, with significant improvement often reported for visual scanning and search performance which, in turn, may underpin the significant improvements in daily activities and QoL despite no objective improvement (albeit not expected) in measurements of visual field parameters. The results further suggest that different types of compensatory scanning strategies are appropriate for different types of activities; for example, task specific to visual exploration of the environment versus specific to reading performance. There is not enough evidence that visual field substitutive/restitutive training can substantially adjust or improve the area of visual field loss and potential risk of adverse events. However, further research is required to evaluate how such interventions could improve sensitivity and discrimination awareness within the affected area of visual field loss, even though the static visual field loss itself persists. No information was reported about effect on falls rate or driving performance. There remains a need for further studies to comprehensively evaluate the effectiveness of suggested interventions for hemianopia, particularly for visual scanning/search training. To that end, we anticipate the outcomes of current trials of interventions for hemianopia (Eye movement training in visual field defect patients by using a 3D game; Reading training for people with hemianopia; Visual scanning training for loss of vision in hemianopia – SEARCH trial) (ISRCTN trial registry).^96^


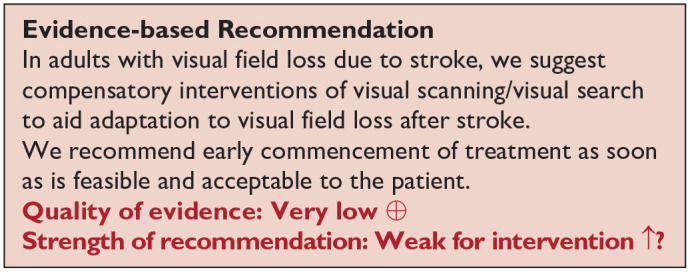


***PICO 9:* For adults with ocular stroke (central retinal artery occlusion), does compensatory, substitute or restitutive intervention, compared to no intervention, improve activities and quality of daily life?**

### Analysis of current evidence

In this PICO, we consider whether compensatory, substitute or restitutive interventions can improve activities and quality of daily life in stroke patients with ocular stroke (also interchangeably termed central retinal artery occlusion (CRAO), eye stroke or retinal stroke). For the purpose of the present guidelines, we define compensatory, substitutive and restitutive interventions as treatment options to improve adaptation to the impairment (compensatory, e.g. eccentric viewing), to improve the visual impairment using a device or optical aid (substitutive, e.g. spectacles, magnifier) and to restore visual function (restitution, e.g. thrombolysis). We considered any point in the stroke pathway. However, we were interested, particularly, in the hyperacute setting regarding timely intervention aligned with thrombolysis.

For this PICO, we included outcomes that were rated as critical by the writing group. These included change in visual acuity, quality of life and activities of daily living.

We identified five relevant studies (see Supplemental Table 13), comparing various interventions for ocular stroke.^97–101^ The mean sample size was 73.6 (SD 47.5; median 60; range 25–134). None of the studies were RCTs, all were case comparison or cohort studies that compared interventions to standard care.

**Figure 9.**
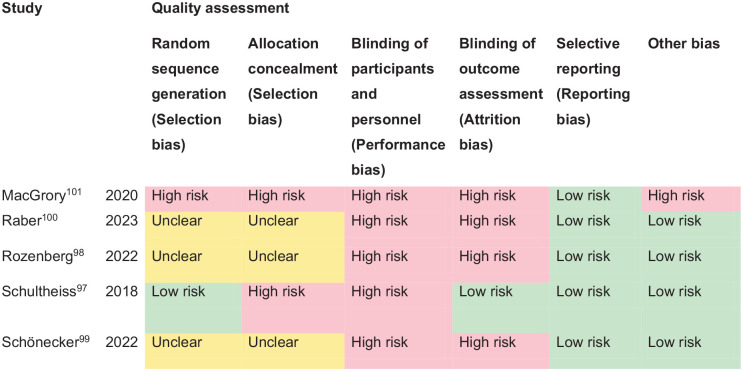
PICO 9 – Risk of bias assessment.

Four were intravenous thrombolysis interventions: recombinant tissue plasminogen activator (rtPA/alteplase).^97,99,100,101^ Another study focussed on hyperbaric oxygen therapy.^98^ This evidence, while limited, suggests that thrombolysis and hyperbaric oxygen therapy may offer beneficial outcomes for some ocular stroke survivors. However, no RCTs have been published in the period of 2011-2023. Four studies reported change in visual acuity and two reported change in functional activities.^97–101^ The latter are important as they underscore the potential of thrombolysis to significantly enhance independence in daily activities for patients experiencing ocular stroke, in addition to an improvement in vision. There were no reports of quality of life as outcomes. We also documented adverse events given the importance of thrombolysis treatment specific to CRAO. Table 9.1 and Figure 9 show the GRADE assessment of interventions for ocular stroke and visual acuity change outcomes reported in each study. These studies had a high risk of bias. Limitations included study heterogeneity, blinding of participants/investigators, unblinded interpretation of test results and limited information on complete or missing data. Hence, meta-analysis of data was not possible.

**Table 9.1.** PICO 9 – For adults with ocular stroke, does compensatory, substitute or restitutive intervention, compared to no intervention, improve activities and quality of daily life? Summary of findings for PICO 9. Assessment of the interventions for ocular stroke. Participants: Ocular stroke – retinal artery occlusion Settings: Acute. Intervention: Restitutive. Reference standard: Control, standard or conservative care.

Outcome	*N* participants	Effect sham/standard care	Effect intervention	Significance between groups	Quality of evidence (GRADE)
**Change in visual acuity** logMAR^101^	MacGrory: 25 intervention 87 standard care	Change from baseline to 48 h Mean 0.3 ± 0.7 logMAR	Change from baseline to 48 h Mean 1.0 ± 1.11 logMAR 56.3% improvement of ⩽0.3 43.8% improvement of ⩽0.7 31.2% improvement of ⩽0.5	S: *p* = 0.001	+ooo Very low^a^
logMAR^97^	Schultheiss: 20 intervention 40 standard care	Change from baseline to 30 days Pre: Mean 2.09 ± 0.51 logMAR Post: Mean 1.63 ± 0.62 logMAR	Change from baseline to 30 days Pre: Mean 2.46 ± 0.33 logMAR Post: Mean 1.60 ± 1.08 logMAR	S: *p* = 0.004	
logMAR^100^	Raber: 16 intervention 21 conservative	Change from baseline to 24 h Pre: Median 2.30 logMAR (IQR 1.90–2.70) Post: Median 2.30 logMAR (IQR 1.60–2.30)	Change from baseline to 24 h Pre: Median 2.30 logMAR (IQR 2.10–2.30) Post: Median 2.10 logMAR (IQR 1.45–2.30)	S: *p* < 0.002	
logMAR^98^	Rozenberg: 121 intervention 23 standard care	Change from baseline to 12.9 ± 34 months Pre: Mean 3.04 ± 0.82 logMAR Post: Mean 2.80 ± 1.50 logMAR	Change from baseline to 51.5 ± 57 months Pre: Mean 2.89 ± 0.98 logMAR Post: Mean 2.15 ± 1.07 logMAR	S: *p* < 0.001	
**Change in activities of daily living** Modified Rankin scale^99^	Schonecker: 9 intervention 16 standard care	Change from baseline to 12 months Mean 0.1 ± 0.6	Change from baseline to 12 months Mean 0.9 ± 0.9	S: *p* = 0.006	+ooo Very low^a^
Functional reading ability^97^	Schultheiss 20 intervention 40 standard care	Change from baseline to 24 h 5% improvement	Change from baseline to 24 h 25% improvement	S: *p* = 0.045	
**Adverse events** ^97–101^	MacGrory: 25 intervention 87 standard care		*N* = 1 asymptomatic intracranial haemorrhage		+ooo Very low^a^
	Schultheiss: 20 intervention 40 standard care		*N* = 1 angioedema *N* = 1 aortic aneurysm haemorrhage		
	Raber: 16 intervention 21 conservative		None noted		
	Rozenberg: 121 intervention 23 standard care		*N* = 2 ear barotrauma *N* = 1 seizures and epistaxis		
	Schonecker: 9 intervention 16 standard care		*N* = 1 asymptomatic intracranial haemorrhage		

a Downgraded due to risk of bias, limited precision, and potential publication bias.

### Additional information

Additionally, we found one non-comparator cohort study evaluating intravenous liposomal prostaglandin E1 as an intervention for acute CRAO (Supplemental Table 13).^102^

In addressing ocular stroke, a critical condition affecting visual acuity and ocular function, overall five studies focused on the efficacy of repurfusion treatment, particularly the administration of rtPA (alteplase) within a 4.5-h window.^97,99–102^ Consistent reporting of improved visual acuity, with significant improvement in visual acuity of 0.7–1.0 logMAR, after thrombolysis treatment (rtPA/alteplase within 4.5 h) indicate the value of timely intervention in cases of ocular stroke. However, lesser improvement with treatments initiated after the 4.5-h window highlight the critical timing for intervention effectiveness.

The findings also suggest that while thrombolysis can be beneficial, its effectiveness may depend on various factors, including the specifics of the ocular stroke event and patient characteristics. While rtPA/alteplase for CRAO is reported as feasible and safe, and with improved visual function compared with non-treatment, data from RCTs with intravenous thrombolysis given within 4.5 h time window is still lacking.^101,103^ To that end, we anticipate the outcomes of current trials of alteplase or tenecteplase for ocular stroke (THEIA (A Phase III Randomized, Blind, Double Dummy, Multicentred Study Assessing the Efficacy and Safety of IV Thrombolysis; Alteplase); TenCRAOS (TENecteplase in Central Retinal Artery Occlusion Study); and REVISION (Early Reperfusion Therapy With Intravenous Alteplase for Recovery of VISION in Acute Central Retinal Artery Occlusion)).^104–106^

This research on the treatment of ocular stroke underscores the critical importance of timely intervention and its potential impact on activities of daily living, such as functional reading ability. This aspect of care is paramount, considering the significant apprehension people feel towards the loss of sight, which profoundly influences their quality of life and independence. The significant difference in activity of daily living outcomes between the intervention and control groups points to the efficacy of thrombolysis as a potentially superior therapeutic strategy for improving activities of daily living among ocular stroke survivors. This finding emphasises the need for clinicians to consider thrombolysis as a viable treatment option for eligible patients, potentially setting a new standard of care that prioritises functional recovery and quality of life.

The willingness of stroke survivors to engage in treatment parallels the urgency observed in those with cerebral strokes, although concerns remain for those relying on a single unaffected eye, highlighting the diversity in patient perspectives and the need for personalised care strategies.

Analysis of disposition among ocular stroke survivors towards intervention indicates a broad willingness comparable to that observed in brain stroke survivors, because of the inherent fear of losing sight, the most treasured of senses.^107,108^ A small subset of stroke survivors, however, may decline interventions, especially those with one remaining unaffected eye. A further noteworthy consideration in the acceptance of this intervention is the occurrence of adverse events, which includes instances of intracerebral hemorrhage (1%), orolingual angioedema and systemic bleeding (up to 11%).^97,99,100^ These incidents are crucial for understanding patient hesitancy and weighing the benefits against potential risks: important information for discussion in requesting treatment consent. It is also important to highlight the increased risk of cerebral stroke within the short time window after CRAO with referral for appropriate cerebrovascular work-up, just as is the case for transient ischemic attack and minor stroke, as a preventative measure.

The future treatment of ocular stroke will require close collaboration between family/general practitioners, primary eye care services (e.g. optometry) and hospital services for ophthalmology and stroke physicians/neurologists.


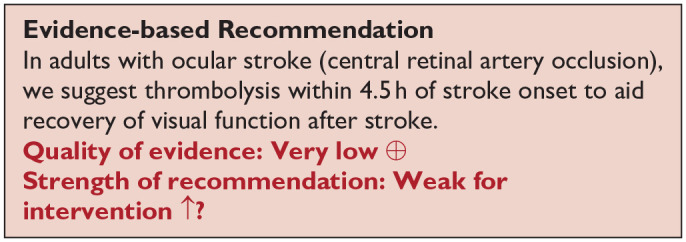


***PICO 10:* For adults with central vision impairment due to stroke, does compensatory, substitute or restitutive intervention, compared to no intervention, improve activities and quality of daily life?**

### Analysis of the current evidence

In this PICO, we consider whether compensatory, substitute or restitutive interventions can improve activities and quality of daily life in stroke patients with central vision impairment. Central vision impairment in this PICO excluded studies specific to central retinal artery occlusion which are discussed separately in PICO 9. For the purpose of the present guidelines, we define compensatory, substitutive and restitutive interventions as treatment options to improve adaptation to the impairment (compensatory, e.g. eccentric viewing), to improve the visual impairment using a device or optical aid (substitutive, e.g. spectacles, magnifier) and to restore visual function (restitution). We considered any point in the stroke pathway.

We found no randomised controlled trials comparing interventions for central visual impairment compared to no intervention, sham intervention or placebo.

### Additional information

**Figure 10.**
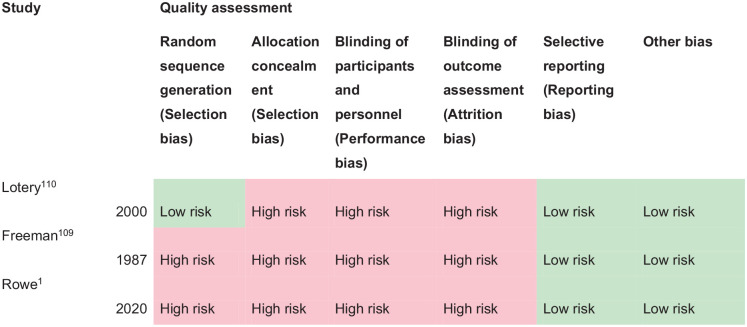
PICO 10 – Risk of bias assessment.

In this PICO, we included outcomes that were rated as critical by the writing group, specifically change in visual acuity and quality of life. We found three observation cohort studies (Supplemental Table 14) documenting change in visual acuity after intervention, with a median sample size of 273 (range 77–1500; mean 616.7, SD 771.2). The median number of those visually assessed was 77 (range 55–1204; mean 445.3, SD 657.1).^1,101,109,110^ No study reported the outcomes of activities of daily living and/or quality of life. Table 10.1 and Figure 10 show the GRADE assessment. Meta analysis was not possible due to considerable heterogeneity across included trials with different interventions, outcome measures and timing of treatment post stroke. Most studies had a high risk of bias. Limitations included study heterogeneity, unblinded interpretation of test results and limited information on complete or missing data.

**Table 10.1.** Summary of findings for PICO 10. Assessment of the interventions for central vision impairment due to stroke. Participants: Stroke survivors with central vision impairment. Settings: Acute. Intervention: Restitutive.

Outcome	*N* participants	Effect sham/standard care	Effect intervention	Significance between groups	Quality of evidence (GRADE)
**Change in visual acuity** Snellen/logMAR^109^	Freeman: 24 of 55	N/A	Change over 6 months: mean 63 days *N* = 12 partial/full recovery, *N* = 5 no recovery, remainder not reviewed	N/A	+ooo Very low^a^
Snellen/logMAR^110^	Lotery: 20 of 77	N/A	Change from baseline to 2 weeks *N* = 11 partial/full recovery with glasses	N/A	
logMAR^1^	Rowe: 354 of 1204	N/A	Change from baseline to 1 year *N* = 126 full recovery, *N* = 129 partial recovery, *N* = 90 no recovery, remainder not reviewed Near visual acuity: Pre: Right/left eye Mean 0.6 (SD 0.356)/Mean 0.61 (SD 0.483) Post: Right/left eye Mean 0.45 (SD 0.279)/Mean 0.50 (SD 0.506) Distance visual acuity: Pre: Right/left eye Mean 0.5 (SD 0.562)/Mean 0.53 (SD 0.594) Post: Right/left eye Mean 0.33 (SD 0.456)/Mean 0.44 (SD 0.793)	N/A	

a Downgraded due to risk of bias, indirectness, limited precision and potential publication bias.

Freeman and Rudge reported a prospective cohort study of stroke survivors receiving specialist orthoptic assessment on the stroke unit.^109^ Of 55 stroke survivors with visual acuity testing, 24 (44%) had impaired visual acuity. Visual acuity improved for 50% (*n* = 12) over an average 63 days (range 1 week to 6 months). Intervention was provision of updated/new spectacles for five cases. Improvement for the remainder was spontaneous. Lotery et al. recruited 77 stroke survivors in a prospective observation study with the aim of evaluating a full visual assessment within 2 weeks of admission on a stroke rehabilitation unit.^110^ Impaired visual acuity worse than 0.3 logMAR was documented for 26% (*n* = 20) of stroke survivors. Intervention was provision of updated/new spectacles and visual acuity improved for half because of having new or updated glasses (*n* = 10). Rowe et al., in a prospective epidemiology study of 1500 consecutive stroke admissions reported findings of visual assessment from 1204 of the overall cohort.^1^ They reported a mean change (in measured visual acuity from first to last visits) in near binocular visual acuity of 0.33 logMAR and a mean change in distance binocular visual acuity of 0.19 logMAR. Full recovery was documented for 35.6% of stroke survivors, partial for a further 36.4% and no improvement for 25.4% stroke survivors, over a mean follow-up of 93.75 days (SD 102.84): median 58 days (range 1–530). Interventions included referral to optometry or low vision services (51.7%) or provision of compensatory strategies and information resources (25.4%).

It is important to note for central visual impairment, that reduction or loss of visual acuity can be due to the stroke event, and pre-existing ocular pathology/refractive error, or a combination. While Freeman and Rudge, and Lotery et al., did not distinguish between new onset versus pre-existing central visual impairment, Rowe et al. categorised their cases.^1,109,110^ Co-existent ocular pathology was reported for about 30% with childhood strabismus/amblyopia accounting for a further 5.4%.^1^ They reported incidence of new onset stroke-related central visual impairment for 29.4% (*n* = 354 stroke survivors) ^1^. Regardless of new onset or pre-existing deficit, it is important to intervene to improve visual acuity in order to promote better visual function for safety of mobilisation and to facilitate greater engagement with general rehabilitation.

There is limited evidence from the included studies to show a percentage of improved visual acuity over time after stroke onset, either spontaneously or because of new/updated spectacles prescription. Change in visual acuity was reported through improvement in level of visual acuity as well as the proportion of those who had change in visual acuity. There was variation across studies. For example, the specific intervention was not always specified: some included standard care, there was a range of different interventions delivered (including advice, typoscopes) and specific single interventions were employed, for example, spectacle prescription. Currently there is a need for methodologically robust studies to evaluate the impact of interventions on improving visual acuity, activities of daily living and/or quality of life of stroke survivors with impaired central vision.

In line with previous systematic reviews on this subject, we agree that evidence relating to the management of patients (from the general population) with age-related visual problems is available from other Cochrane reviews.^15,19^ This continues to be the best evidence available for making treatment decisions about individual patients. When considering patient preferences and values, it is likely that stroke survivors want an intervention to improve their visual acuity compared to no intervention.

Clinicians should provide information to stroke survivors and their caregivers specific to reading aids, electronic aids, filters (e.g. contrasting enhancing/polarised), and environment modifications along with appropriate information and resource materials. Further, it is well-recognised that many stroke survivors will have worn spectacles prior to their stroke. It is therefore important that they have access to their spectacles or receive a retest for spectacles (if lost/broken/old) after their stroke. For those patients who still have reduced central vision even with spectacles correction, low visual aids such as magnifiers may be helpful. Persistently reduced visual acuity post-stroke warrants referral for further ophthalmic evaluation to optometry and ophthalmology services.


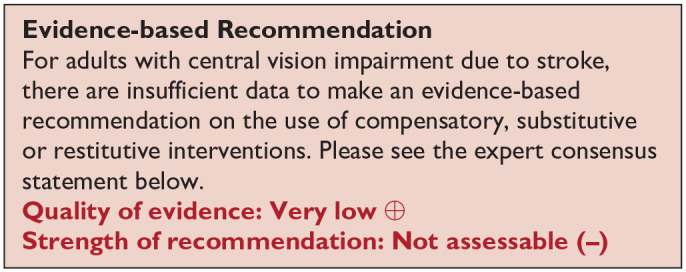



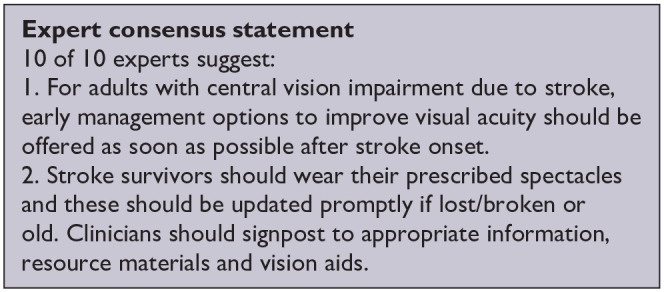


***PICO 11:* For adults with eye movement disorders due to stroke, does compensatory, substitute or restitutive intervention, compared to no intervention, improve activities and quality of daily life?**

### Analysis of current evidence

In this PICO, we consider whether compensatory, substitute or restitutive interventions can improve activities and quality of daily life in stroke patients with eye movement disorders. For the purpose of the present guidelines, we define compensatory, substitutive and restitutive interventions as treatment options to improve adaptation to the impairment (compensatory, e.g. eye scanning training), to improve the visual impairment using a device or optical aid (substitutive, e.g. prism, occlusion) and to restore ocular alignment and motility (restitution, e.g. extraocular muscle surgery). We considered any point in the stroke pathway.

**Figure 11.**
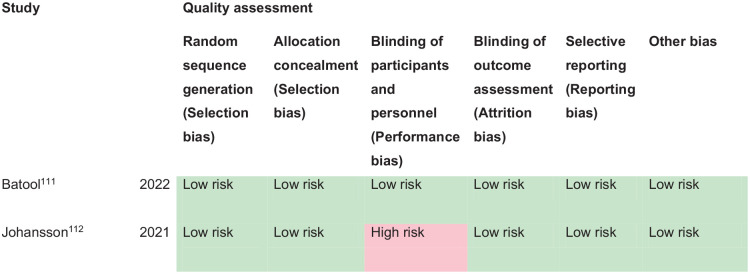
PICO 11 – Risk of bias assessment.

For this PICO, we included outcomes that were rated as critical by the writing group. These included change in eye movement range, activities of daily living, and quality of life. Overall, we found two relevant studies (one RCT, one observation case control study; Supplemental Table 15) using compensatory interventions compared to a control of no intervention or standard care.^111,112^. Mean sample size was 76.5 (SD 17.7; median 76.5, range 64–89). One trial included any ocular motility disturbance due to stroke^111^ and the second case control study addressed binocular vision dysfunction.^112^ Table 11.1 and Figure 11 show the GRADE assessment of interventions for eye movement disorders. Meta analysis was not possible due to considerable heterogeneity across included trials with different interventions, outcome measures and timing of treatment post stroke. These studies had a high risk of bias where limitations included study heterogeneity, unblinded interpretation of test results and limited information on complete or missing data.

**Table 11.1.** PICO 11 – For adults with eye movement disorders due to stroke, does compensatory, substitute or restitutive intervention, compared to no intervention, improve activities and quality of daily life? Summary of findings for PICO 11. Assessment of the interventions for eye movement disorders due to stroke. Participants: Stroke survivors with eye movement disorders. Settings: Acute. Intervention: Compensatory. Reference standard: Control, standard or conservative care.

Outcome	*N* participants	Effect sham/standard care	Effect intervention	Significance between groups	Quality of evidence (GRADE)
**Change in eye movements** Convergence^112^ Convergence facility^112^ Distance vergence reserve^112^ Near vergence reserve^112^	Johansson: 48 intervention 41 standard care	Change from baseline to 8 weeks Pre: Median 20 Post: Median 12 NS Pre: Median 0 Post: Median 3 NS Pre: Median 12 Post: Median 14 *p* = 0.04 Pre: Median 23 Post: Median 27 NS	Change from baseline to 8 weeks Pre: Median 20 Post: Median 15 *p* = 0.02 Pre: Median 0 Post: Median 6 *p* = 0.03 Pre: Median 15 Post: Median 22 *p* < 0.01 Pre: Median 23 Post: Median 28 *p* < 0.01	N/A N/A N/A N/A	+ooo Very low^a^
**Change in activities of daily living** Berg Balance scale^111^ Barthel Index scale^111^	Batool: 32 intervention 32 sham	Change from baseline to 4 weeks Pre: Mean 11.19 ± 2.18 Post: Mean 12.63 ± 2.52 *p* = 0.0001 Pre: Mean 20.31 ± 7.72 Post: Mean 26.25 ± 10.70 *p* = 0.0001	Change from baseline to 4 weeks Pre: Mean 10.75 ± 2.17 Post: Mean 16.34 ± 2.88 *p* = 0.0001 Pre: Mean 18.28 ± 7.47 Post: Mean 32.66 ± 12.69 *p* = 0.0001	S: *p* = 0.0001 S: *p* = 0.033	++++ High
**Change in quality of life** Convergence insufficiency symptom score^112^	Johansson 48 intervention 41 standard care	Change from baseline to 8 weeks Pre: Median 20 Post: Median 15 NS	Change from baseline to 8 weeks Pre: Median 20 Post: Median 15 *p* < 0.01	*p* < 0.01	+ooo Very low^a^

a Downgraded due to risk of bias and potential publication bias.

### Additional information

We found a further three studies using compensatory and substitutive interventions in single group cohort studies.^1,113,114^ Median sample size was 30 (range 20–1500; mean 516.7, SD 851.6) and median number that were visually assessed was 30 (range 20–1204; mean 418, SD 680.7). One study included any ocular motility disturbance due to stroke^1^ and a further two studies addressed binocular vision dysfunction^113,114^ (Supplemental Table 15).

Significant improvement in binocular vision functions (e.g. vergence eye movements and fusional reserves), activities of daily living and quality of life were documented from eye movement training regimes which typically included a combination of clinician-delivered and home-based exercises. However, in reference to the many and varied eye movement disorders that can arise specifically due to stroke, treatment effect could not be determined specifically for cases caused by stroke.

This does not, in any way, infer that interventions for eye movement disorders are ineffective. There are interventions which have been investigated in eye movement disorders due to acquired brain injuries other than stroke that are relevant for stroke patients with the same eye movement disorders, such as prisms, botulinum toxin and extraocular muscle surgery.^115–117^ Prisms have been in clinical use for many decades and are accepted by the clinical community to be effective for the management of eye movement disorders in correcting binocular diplopia.^17,118^ Such interventions do not require further research evaluation given their well-established validated use and proven clinical and cost effectiveness. Similarly, where prisms are not indicated for early correction of diplopia, a patch or other suitable occlusion option is recommended. Typically, the prism or patch/occlusion is placed over the affected eye unless patient preference dictates otherwise, for example, in cases of strong ocular dominance, and can be a total or sector placement on glasses. Further, in addition to use of an eye patch, occlusion in the form of varied extents of blur, can be used, for example, Blenderm, Micropore or Bangerter tape/foils. This can be partial or total occlusion (eye patching) with a caveat of caution where total occlusion is used, thus rendering the patient monocular with potential impact from loss of visual input from the occluded eye.


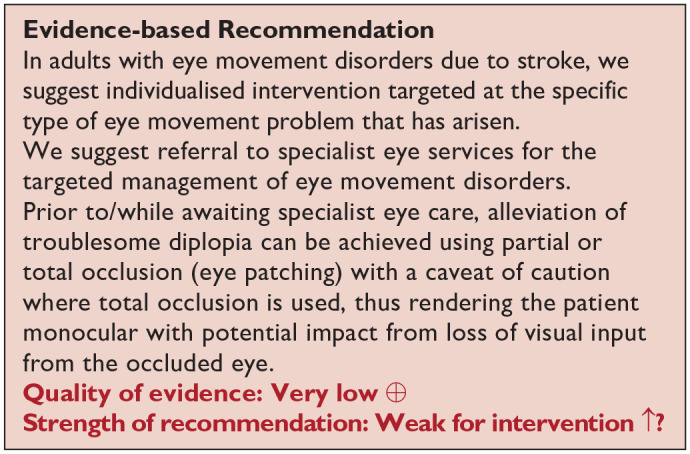


***PICO 12:* For adults with visual neglect due to stroke, does compensatory, substitute or restitutive intervention, compared to no intervention, improve activities and quality of daily life?**

### Analysis of current evidence

In this PICO, we considered whether compensatory, substitute or restitutive interventions can improve activities and quality of daily life in stroke patients with visual neglect/inattention. For the purpose of the present guidelines, we define compensatory, substitutive and restitutive interventions as treatment options to improve adaptation to the impairment (compensatory, e.g. visual scanning training), to improve the visual impairment using a device or optical aid (substitutive, e.g. prisms) and to restore visual attention (restitution). We considered any point in the stroke pathway.

**Figure 12.**
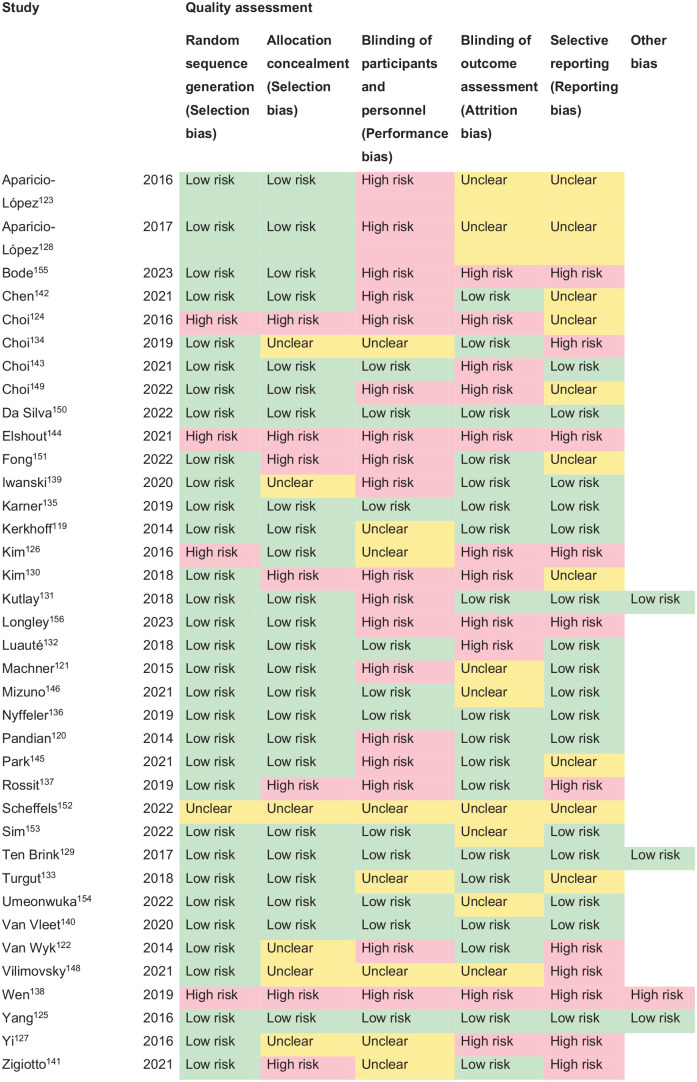
PICO 12 – Risk of bias assessment.

For this PICO, we included outcomes that were rated as critical by the writing group. These were change in visual neglect and change in activities of daily living and quality of life. Overall, we found 44 studies (Supplemental Table 16) of relevance to this PICO with comparison of various interventions.^119–163^ These included 37 (including cross-over) randomised control trials (RCTs)^119–156^ and seven cohort/cross-sectional studies,^157–163^ with a median sample size of 30 (range 20–426; mean 51.0, SD 75.7). Table 12.1 and Figure 12 show the GRADE assessment of RCT interventions for visual neglect. All trials/studies reported measures of activities of daily living and two reported additionally on quality of life. Meta analysis was not possible due to considerable heterogeneity across included trials with different interventions, outcome measures and timing of treatment post stroke. The majority of studies had a high risk of bias in which limitations included study heterogeneity, unblinded interpretation of test results and limited information on complete or missing data.

**Table 12.1.** PICO 12 – For adults with visual neglect due to stroke, does compensatory, substitute or restitutive intervention, compared to no intervention, improve activities and quality of daily life? Summary of findings for PICO 12. Assessment of the interventions for visual neglect due to stroke A Participants: Stroke survivors with visual neglect. Settings: Acute. Intervention: Restitution – computerised therapy. Reference standard: Control, standard or conservative care.

Outcome	*N* participants	Effect sham/standard care	Effect intervention	Significance between groups	Quality of evidence (GRADE)
**Change in visual neglect** Bell cancellation^123^ Figure copying^123^ Line bisection^123^ (rightward deviations) Line bisection (leftward deviations)	Aparicio-Lopez: 13 intervention 15 single therapy	Change from baseline to 5 days Pre: Mean 20.07 (SD 9.16) Post: Mean 29.67 (SD 3.83) *p* = 0.001 Pre: Mean 2.13 (SD 1.72) Post: Mean 0.60 (SD 0.98) *p* = 0.016 Pre: Mean 20.58 (SD 10.39) Post: Mean 11.28 (SD 6.13) *p* = 0.002 Pre: Mean −9.44 (SD 7.65) Post: Mean −9.64 (SD 6.85) *p* = 0.96	Change from baseline to 5 days Pre: Mean 14.08 (SD 9.35) Post: Mean 23.15 (SD 9.20) *p* = 0.003 Pre: Mean 2.46 (SD 1.89) Post: Mean 1.62 (SD 1.75) *p* = 0.131 Pre: Mean 27.51 (SD 16.70) Post: Mean 21.13 (SD 16.39) *p* = 0.019 Pre: Mean −7.23 (SD 7.42) Post: Mean −13.82 (SD 11.30) *p* = 0.248	NS: *p* = 0.856 NS: *p* = 0.496 NS: *p* = 0.316 NS: *p* = 0.417	+ooo Very low^a^
Bell cancellation^128^ Figure copying^128^ Line bisection^128^	Aparicio-Lopez: 13 intervention 18 Single therapy	Change from baseline to 12 weeks Pre: Mean 19.71 (SD 10.09) Post: Mean 28.43 (SD 4.11) *p* = 0.041 Pre: Mean 1.71 (SD 1.38) Post: Mean 1.43 (SD 1.13) *p* = 0.414 Pre: Mean −10.07 (SD 6.62) Post: Mean −9.74 (SD 4.04) *p* = 0.866	Change from baseline to 12 weeks Pre: Mean 11.14 (SD 7.26) Post: Mean 20.29 (SD 9.86) *p* = 0.028 Pre: Mean 3.29 (SD 1.49) Post: Mean 2.14 (SD 2.03) *p* = 0.157 Pre: Mean −7.59 (SD 7.99) Post: Mean −10.25 (SD 12.54) *p* = 0.176		
Posner cueing task^140^	Van Vleet: 14 intervention 14 control	Change from baseline to 6 months 148.22 ms more rightward bias	Change from baseline to 6 months 153.92 ms less rightward bias	NS: *p* = 0.16	
Line bisection (deviation from midline)^143^	Choi: 12 intervention 12 control	Change from baseline to 4 weeks Pre: Mean 7.83 ± 6.28 Post: Mean 9.67 ± 6.61 *p* = 0.005	Change from baseline to 4 weeks Pre: Mean 8.25 ± 5.89 Post: Mean 11.75 ± 5.83 *p* = 0.002	S: *p* = 0.02	
**Change in activities of daily living** Catherine Bergego scale^123^	Aparicio-Lopez: 13 intervention 15 Single therapy	Change from baseline to 5 days Pre: Mean 7.13 (SD 6.48) Post: Mean 6.14 (SD 6.13) *p* = 0.925	Change from baseline to 5 days Pre: Mean 6.82 (SD 6.87) Post: Mean 4.40 (SD 3.37) *p* = 0.064	NS: *p* = 0.254	+ooo Very low^a^
Catherine Bergego scale^128^	Aparicio-Lopez: 13 intervention 18 Single therapy	Change from baseline to 12 weeks Pre: Mean 10.08 (SD 7.29) Post: Mean 9.34 (SD 8.20) *p* = 0.753	Change from baseline to 12 weeks Pre: Mean 9.35 (SD 8.49) Post: Mean 6.28 (SD 3.26) *p* = 0.236		
Combined Barthel index and Catherine Bergego scale^140^	Van Vleet: 14 intervention 14 control	Change from baseline to 6 months 0.09 ± 0.78 (95%CI: −0.24 to 0.43)	Change from baseline to 6 months −0.09 ± 0.81 (95%CI: −0.42 to 0.23)	NS: *p* = 0.65	
Catherine Bergego scale^143^ Barthel index^143^	Choi: 12 intervention 12 control	Change from baseline to 4 weeks Pre: Mean 9.33 ± 6.16 Post: Mean 10.42 ± 6.33 *p* = 0.006 Pre: Mean 38.08 ± 9.80 Post: Mean 44.50 ± 10.19 *p* = 0.002	Change from baseline to 4 weeks Pre: Mean 8.33 ± 5.87 Post: Mean 11.25 ± 5.03 *p* = 0.003 Pre: Mean 37.42 ± 8.73 Post: Mean 47.17 ± 9.73 *p* = 0.003	NS: *p* = 0.052 NS: *p* = 0.143	
**Change in quality of life** SF-12^140^	Van Vleet: 14 intervention 14 control	Change from baseline to 6 months Physical Health 1.5 ± 10.8 (95%CI: −5.0 to 8.2) Mental Health −1.4 ± 7.8 (95%CI: −6.1 to 3.4)	Change from baseline to 6 months Physical Health −1.6 ± 9.4 (95%CI: −6.7 to 3.7) Mental Health 5.2 ± 11.2 (95%CI: −1.0 to 11.5)	NS: *p* = 0.35 NS: *p* = 0.16	+ooo Very low^a^

a Downgraded due to risk of bias, inconsistency, lack of precision and potential publication bias.

**Table:** B Participants: Stroke survivors with visual neglect. Settings: Acute. Intervention: Compensatory – visual scanning/visuomotor training. Reference standard: Control, standard or conservative care.

Outcome	*N* participants	Effect sham/standard care	Effect intervention	Significance between groups	Quality of evidence (GRADE)
**Change in visual neglect** Functional neglect index^119^ Unawareness and behavioural neglect index^119^	Kerkhoff: 12 intervention1 12 intervention2	Change from baseline to 4 weeks Intervention 2 Mean 5.16 *p* < 0.001 Mean 0.37 *p* = 0.001	Change from baseline to 4 weeks Intervention 1 Mean 2.83 *p* = 0.041 Mean ⩽ 0.06 *p* = 1.00	S: *p* = 0.006 S: *p* = 0.004	+ooo Very low^a^
Star cancellation test^122^	Van Wyk: 12 intervention 12 control	Change from baseline to 4 weeks Pre: Mean ~45 Post: Mean ~41	Change from baseline to 4 weeks Pre: Mean ~27 Post: Mean ~50	S: *p* = 0.016	
Clock drawing test^133^ Line bisection^133^ Apples cancellation test^133^	Turgut: 26 – cross over RCT 14 vs 12	Change from baseline to 7 weeks	Change from baseline to 7 weeks	S: *p* = 0.003 S: *p* = 0.045 S: *p* = 0.017	
Behavioural inattention test^137^ Balloon’s cancellation task^137^	Rossit: 10 intervention 10 control	Change from baseline to 4 months Pre: Mean 86.0 (SD 13.0) Post: Mean 102.1 (SD 13.6) *p* < 0.001 Change from baseline to 4 months Pre: Mean 12.2 (SD 6.1) Post: Mean 33.6 (SD 6.7)	Change from baseline to 4 months Pre: Mean 97.1 (SD 7.0) Post: Mean 128.0 (SD 7.7) *p* < 0.001 Change from baseline to 4 months Pre: Mean 25.5 (SD 6.5) Post: Mean 33.7 (SD 6.1)	NS: *p* = 0.059 NS: *p* = 0.664	
Composite neglect score^144^	Elshout: 10 intervention1 10 intervention2	Change from baseline to post 10 sessions Mean 2.7 (SE 5.2) *p* = 0.62	Change from baseline to post 10 sessions Mean −5.99 (SE 2.43) *p* = 0.036	NS: *p* = 0.068	
Neuropsychological test accuracy (%) Bells test (CoC) Line bisection (deviation)^155^	Bode: 20 (cross over RCT)	Change from baseline to 7 weeks Mean 3 (SEM 1) Mean −0.03 (SEM 0.02) Mean −1.3 (SEM 2.8)	Change from baseline to 7 weeks Mean 9 (SEM 2) Mean −0.1 (SEM 0.04) Mean −7 (SEM 3.5)	S: *p* = 0.002 NS: *p* = 0.082 NS: *p* = 0.141	
**Change in activities of daily living** Barthel Index^119^	Kerkhoff: 12 intervention1 12 intervention2	Change from baseline to 4 weeks Intervention 2 Pre: Mean 11 (4) Post: Mean 28 (5) *p* = 0.020	Change from baseline to 4 weeks Intervention 1 Pre: Mean 15 (5) Post: Mean 26 (8) *p* = 0.083	NS: *p* = 0.16	+ooo Very low^a^
Barthel index^122^	Van Wyk: 12 intervention 12 control	Change from baseline to 4 weeks Pre: Mean ~47 Post: Mean ~65 ‘Statistically significant’	Change from baseline to 4 weeks Pre: Mean ~46 Post: Mean ~86	S: *p* = 0.004	
Catherine Bergego Scale Barthel index^133^ Functional independence measure^133^	Turgut: 26 – cross over RCT	Change from baseline to 7 weeks Pre: Mean 10.0 ± 1.9 Post: Mean 8.5 ± 2.1 Pre: Mean 5.0 ± 1.7 Post: Mean 10.0 ± 2.2 Pre: Mean 45.5 ± 3.7 Post: Mean 55.0 ± 5.0	Change from baseline to 7 weeks Pre: Mean 17.0 ± 1.6 Post: Mean 7.5 ± 1.6 Pre: Mean 10.0 ± 2.0 Post: Mean 15.0 ± 2.2 Pre: Mean 41.0 ± 3.8 Post: Mean 55.0 ± 3.9	S: *p* = 0.002	
Stroke impact scale ADL/IADL^137^	Rossit: 10 intervention 10 control	Change from baseline to 4 months Pre: Mean 46.0 (SD 8.7) Post: Mean 46.5 (SD 8.7) *p* = 0.05	Change from baseline to 4 months Pre: Mean 48.1 (SD 9.9) Post: Mean 58.9 (SD 9.3) *p* = 0.06	NS: *p* = 0.823	
Catherine Bergego scale^144^	Elshout: 10 intervention1 10 intervention2	Change from baseline to post 10 sessions 6.25 lower CBS score Significant	Change from baseline to post 10 sessions 3.23 higher CBS score Not significant	S: *p* = 0.01	
Catherine Bergego scale^155^	Bode: 20 (cross over RCT)	Change from baseline to 7 weeks Mean −1.5 (SEM 1.3)	Change from baseline to 7 weeks Mean −3.6 (SEM 1)		

a Downgraded due to risk of bias, inconsistency, lack of precision and potential publication bias.

**Table:** C Participants: Stroke survivors with visual neglect. Settings: Acute. Intervention: Compensatory – combination exercise training. Reference standard: Control, standard or conservative care.

Outcome	*N* participants	Effect sham/standard care	Effect intervention	Significance between groups	Quality of evidence (GRADE)
**Change in visual neglect** Albert test^138^ Shenckenberg test^138^	Wen: 23 intervention 23 control	Change from baseline Pre: Mean 3.31 ± .61 Post: Mean 2.91 ± 0.43 *p* < 0.01 Pre: Mean 2.79 ± 0.22 Post: Mean 2.04 ± 0.21 *p* < 0.01	Change from baseline Pre: Mean 3.24 ± 0.54 Post: Mean 2.12 ± 0.29 *p* < 0.01 Pre: Mean 2.98 ± 0.23 Post: Mean 1.53 ± 0.19 *p* < 0.01	S: *p* < 0.01 S: *p* < 0.01	+ooo Very low^a^
**Change in activities of daily living** Barthel index^138^	Wen: 23 intervention 23 control	Change from baseline Pre: Mean 32.11 ± 4.01 Post: Mean 37.90 ± 4.87 *p* < 0.01	Change from baseline Pre: Mean 30.88 ± 3.62 Post: Mean 46.13 ± 5.11 *p* < 0.01	S: *p* < 0.01	+ooo Very low^a^

a Downgraded due to risk of bias, inconsistency, lack of precision and potential publication bias.

**Table:** D Participants: Stroke survivors with visual neglect. Settings: Acute. Intervention: Compensatory – robot assisted therapy. Reference standard: Control, standard or conservative care.

Outcome	*N* participants	Effect sham/standard care	Effect intervention	Significance between groups	Quality of evidence (GRADE)
**Change in visual neglect** Line bisection^124^ Star cancellation^124^ Albert’s test^124^	Choi: 20 intervention 18 control	Change from baseline to 3 weeks Mean −7.46 ± 10.11 Mean 5.94 ± 4.96 Mean 4.44 ± 2.66	Change from baseline to 3 weeks Mean −11.10 ± 9.40 Mean 7.90 ± 5.46 Mean 3.85 ± 2.08	NS: *p* = 0.306 NS: *p* = 0.325 NS: *p* = 0.477	+ooo Very low^a^
Behavioural inattention test^131^	Kutlay: 28 intervention 25 control	Change from baseline to 4 weeks Pre: Median 98 (IQR 57–110.5) Post: Median 104.5 (IQR 64–123.25) *p* < 0.001	Change from baseline to 4 weeks Pre: Median 110 (IQR 79.5–137) Post: Median 124 (IQR 104.5–140) *p* < 0.001	NS *p* = 0.137	
Cats cancellation test^135^ Line bisection^135^	Karner: 21 intervention 18 control	Change from baseline to 4 weeks Mean 3.33 (SD 4.16) Mean −0.17 (SD 3.10)	Change from baseline to 4 weeks Mean 7.85 (SD 3.68) Mean 1.92 (SD 2.79)	S: *p* < 0.01 NS: *p* > 0.05	
Behaviour inattention test^142^	Chen: 10 intervention 10 control	Change from baseline to 4 weeks Mean 15.70 ± 7.36	Change from baseline to 4 weeks Mean 23.40 ± 7.85	S: *p* = 0.04	
Line bisection^145^ Albert’s test^145^	Park: 12 intervention 12 control	Change from baseline to 4 weeks Mean 0.77 (SE 0.22) *p* < 0.01 Mean 0.83 (SE 0.52) NS	Change from baseline to 4 weeks Mean 3.10 (SE 0.43) *p* < 0.001 Mean 2.66 (SE 0.56) *p* < 0.01	S: *p* < 0.001 S: *p* < 0.05	
**Change in activities of daily living** Barthel index^124^ Catherine Bergego scale^124^	Choi: 20 intervention 18 control	Change from baseline to 3 weeks Mean 12.72 ± 4.00 Mean −4.28 ± 1.53	Change from baseline to 3 weeks Mean 12.55 ± 4.12 Mean −3.70 ± 2.25	NS: *p* = 0.735 NS: *p* = 0.415	+ooo Very low^a^
Functional independence measure^131^	Kutlay: 28 intervention 25 control	Change from baseline to 4 weeks Pre: Median 64 (IQR 54–69.8) Post: Median 79 (IQR 68.3–86.8) *p* < 0.001	Change from baseline to 4 weeks Pre: Median 71 (IQR 60–80.5) Median 82 (IQR 75.5–99) *p* < 0.001	NS: *p* = 0.206	
Scores of Independence index for neurological and geriatric rehabilitation^135^	Karner: 21 intervention 18 control	Change from baseline to 4 weeks Self-care; Mean 5.11 (SD 2.72) Mobility; Mean 2.76 (SD 2.51)	Change from baseline to 4 weeks Self-care; Mean 5.52 (SD 3.94) Mobility; Mean 2.94 (SD 2.12)	S: *p* < 0.01 S: *p* < 0.01	
Barthel index^142^ Catherine Bergego scale^142^	Chen: 10 intervention 10 control	Change from baseline to 4 weeks Mean 21.0 ± 8.89 Mean −4.10 ± 1.73	Change from baseline to 4 weeks Mean 28.90 ± 14.26 Mean −5.40 ± 1.65	NS: *p* = 0.16 NS: *p* = 0.10	
Catherine Bergego scale^145^	Park: 12 intervention 12 control	Change from baseline to 4 weeks Mean 1.25 (SE 0.41) *p* < 0.05	Change from baseline to 4 weeks Mean 4.30 (SE 0.54) *p* < 0.001	S: *p* < 0.001	
**Change in quality of life** WHODAS-2^142^	Chen: 10 intervention 10 control	Change from baseline to 4 weeks Mean −16.20 ± 6.99	Change from baseline to 4 weeks Mean −23.50 ± 7.58	S: *p* = 0.01	+ooo Very low^a^

WHODAS-2: World Health Organisation Disability Assessment Schedule.

a Downgraded due to risk of bias, inconsistency, lack of precision and potential publication bias.

**Table:** E Participants: Stroke survivors with visual neglect. Settings: Acute. Intervention: Substitution – prism therapy. Reference standard: Control, standard or conservative care.

Outcome	*N* participants	Effect sham/standard care	Effect intervention	Significance between groups	Quality of evidence (GRADE)
**Change in visual neglect** Shape cancellation^129^	Ten Brink: 34 intervention 35 control	Change from baseline to 14 weeks Pre: Mean 6.31 (SD 8.41) Post: Mean 2.16 (SD 4.54)	Change from baseline to 14 weeks Pre: Mean 4.56 (SD 5.72) Post: Mean 1.14 (SD 1.98)	NS: *p* = 0.057	+ooo Very low^a^
Line bisection^132^ Line cancellation^132^ Star cancellation^132^	Luaute: 14 intervention 10 control	Change from baseline to 45 days Day 7: Median 6.2 (range −13.9–55.6) Day 45: Median 5.35 (range −18.2–57.4) Day 7: Median 37 (range 18–39) Day 45: Median 37 (range 18–39) Day 7: Median 25 (range 15–54) Day 22: Median 40 (range 16–54)	Change from baseline to 45 days Day 7: Median 10.1 (range −3.5–64.4) Day 45: Median 1.4 (range −4.9–80.2) Day 7: Median 39 (range 13–39) Day 45: Median 38 (range 8–39) Day 7: Median 30 (range 14–52) Day 22: Median 41 (Range 16–49)	NS: *p* = 0.3542 NS: *p* = 0.6601 NS: *p* = 0.5889	
Albert’s test^134^	Choi: 30: 10 in 3 groups	Change from baseline to 3 weeks Group c: Pre: Mean 14.60 ± 2.17 Post: Mean 9.10 ± 1.19 *p* < 0.001	Change from baseline to 3 weeks Group a: Pre: Mean 15.3 ± 3.49 Post: Mean 5.0 ± 2.21 *p* < 0.001 Group b: Pre: Mean 14.0 ± 1.88 Post: Mean 8.0 ± 2.3 *p* < 0.001	S: *p* = 0.001 NS: *p* = 0.29	
Star cancellation^141^	Zigiotti: 10 intervention 10 control	Change from baseline to 11 days 37% accuracy	Change from baseline to 11 days 78% accuracy	S: *p* < 0.001	
Anosognosia score^146^	Mizuno: 15 intervention 19 sham	Change from baseline to discharge (138.3 ± 43.0 days) Pre: Mean 2.91 ± 5.7 Post: Mean −2.28 ± 3.9 *p* < 0.001	Change from baseline to discharge (127 ± 42.2 days) Pre: Mean 2.91 ± 5.4 Post: Mean 0.16 ± 2.4 *p* < 0.001	NS	
Bells test^148^ Line bisection^148^ Scene copying^148^ Average recovery	Vilimovsky: 12 intervention 11 sham	Change from baseline to 4 weeks post-treatment Improved, *p* = 0.006 No effect, *p* > 0.1 Improved, *p* < 0.006 28.6%	Change from baseline to 4 weeks post-treatment Improved, *p* < 0.001 No effect, *p* > 0.1 Improved, *p* < 0.006 39.1%	NS: *p* = 0.280 NS: *p* = 0.387 NS egocentric *p* = 0.527, allocentric *p* = 0.764	
Albert’s test^149^	Choi: 12 intervention 12 intervention2 12 sham	Change from baseline to 4 weeks 1.30 ± 0.67 *p* < 0.01	Change from baseline to 4 weeks Group a; 2.80 ± 0.91 *p* < 0.01 Group b; 1.60 ± 0.84 *p* < 0.01	S: *p* = 0.001	
Apples cancellation test^152^ Line bisection^152^ Clock drawing^152^	Scheffels: 10 intervention 13 control	Change from baseline to 15 days Improved *p* < 0.001 Improved *p* = 0.01 Improved *p* = 0.05	Change from baseline to 15 days Improved *p* < 0.001 Improved *p* = 0.01 Improved *p* = 0.05	NS NS NS	
Hearts cancellation^156^ Star cancellation^156^ Kessler Foundation neglect assessment^156^	Longley: 32 intervention 7 control	Change from baseline to 12 weeks Pre: Median 13 (IQR 3–17) Post Mean 47.3 (SD 2.5) Pre: Median 46 (IQR 26–49) Post Median 25 (IQR NA) Pre: Median 16 (IQR 10–18) Post: Median 2.2 (IQR–3.3)	Change from baseline to 12 weeks Pre: Median 17 (IQR 9–24) Post: Mean 30.6 (SD 12.3) Pre: Median 14 (8–41) NA Pre: Median 18 (IQR 11–23) Post: Median 1.1 (IQR 0–3.8)	NS NS NS	
Neglect recovery BIT-C total^154^	Umeonwuka: 37 intervention 37 control	Change from baseline to 16 days Recovered *n* = 3 (8.11%) Pre: Mean 82.95 ± 20.46 Post: Mean 78.08 ± 33.74	Change from baseline to 16 days Recovered *n* = 23 (62.16%) Pre: Mean 70.59 ± 23.40 Post: Mean 117.68 ± 39.49	S: *p* < 0.001 S: *p* < 0.001	
**Change in activities of daily living** Catherine Bergego scale^129^	Ten Brink: 34 intervention 35 control	Change from baseline to 6 weeks Pre: Mean 15.43 (SD 7.54) Post: Mean 11.04 (SD 7.94)	Change from baseline to 6 weeks Pre: Mean 12.83 (SD 6.62) Post: Mean 9.46 (SD 5.46)	NS: *p* = 0.133	+ooo Very Low^a^
Catherine Bergego scale^132^	Luaute: 14 intervention 10 control	Change from baseline to 45 days Day 7: Median 17 (range 1–28) Day 45: Median 17 (range 4–23)	Change from baseline to 45 days Day 7: Median 15 (range 5–27.5) Day 45: Median 11.1 (range 2–27.5)	S: *p* = 0.0204	
Catherine Bergego scale^134^	Choi: 30: 10 in 3 groups	Change from baseline to 3 weeks Group c: Pre: Mean 20.10 ± 2.76 Post mean 14.80 ± 3.04 *p* < 0.001	Change from baseline to 3 weeks Group a: Pre: Mean 20.9 ± 2.99 Post: Mean 10.8 ± 2.78 *p* < 0.001 Change from baseline to 3 weeks Group b: Pre: Mean 19.0 ± 2.98 Post: Mean 12.7 ± 3.88 *p* < 0.001	S: *p* < 0.001	
Catherine Bergego scale (observed score)^141^	Zigiotti: 10 intervention 10 control	Change from baseline to 2 weeks Pre score 12.4 Post score 18.1 *p* = 0.049	Change from baseline to 2 weeks Pre score 10.8 Post score 13.2 *p* = 0.002		
Catherine Bergego scale^146^	Mizuni: 15 intervention 19 sham	Change from baseline to discharge (138.3 ± 43.0 days) Pre: Mean 9.61 ± 6.1 Post: Mean 3.44 ± 3.9 *p* < 0.001	Change from baseline to discharge (127 ± 42.2 days) Pre: Mean 9.78 ± 6.8 Post: Mean 2.83 ± 3.3 *p* < 0.001	NS	
Catherine Bergego scale^148^	Vilimovsky: 12 intervention 11 sham	Change from baseline to 4 weeks post-treatment Pre: Median 14 (IQR 12–17) Post: Median 4 (IQR 3–5) Improved, *p* < 0.001	Change from baseline to 4 weeks post-treatment Pre: Median 13 (IQR 12.5–20) Post: Median 4 (IQR 3–7) Improved, *p* < 0.001	NS: *p* = 0.539	
Catherine Bergego scale^149^ Barthel index^149^	Choi: 12 intervention 12 intervention2 12 sham	Change from baseline to 4 weeks 4.70 ± 1.89 *p* < 0.01 9.30 ± 1.63 *p* < 0.01	Change from baseline to 4 weeks Group a; 6.20 ± 1.85 *p* < 0.01 Group b; 4.20 ± 1.81 *p* < 0.01 Group a; 10.50 ± 2.71 *p* < 0.01 Group b; 9.10 ± 2.53 *p* < 0.01	S: *p* = 0.041 NS: *p* = 0.333	
Barthel index^152^ Functional independence measure^152^	Scheffels: 10 intervention 13 control	Change from baseline to 15 days Improved *p* < 0.001 Improved *p* = 0.01	Change from baseline to 15 days Improved *p* < 0.001 Improved *p* = 0.01	NS NS	
Nottingham extended ADL^156^	Longley: 32 intervention 7 control	Change from baseline to 12 weeks Mean 7.9 (SD 6.8)	Change from baseline to 12 weeks Mean 7.3 (SD 5.2)	NS	
Catherine Bergego scale^154^	Umeonwuka: 37 intervention 37 control	Change from baseline to 16 days Pre: Mean 20.24 ± 4.18 Post: Mean 19.5 ± 4.28	Change from baseline to 16 days Pre: 21.22 ± 4.02 Post: Mean 13.85 ± 5.73	S: *p* < 0.001	
**Change in quality of life** WHODAS^157^	Chen: 10 intervention 10 control	Change from baseline to 4 weeks −16.20 ± 6.99	Change from baseline to 4 weeks −23.50 ± 7.58	S: *p* = 0.01	+ooo Very low^a^

ADL: Activities of Daily Living; BIT-C: Behavioral Inattention Test-conventional subtest; WHODAS-2: World Health Organisation Disability Assessment Schedule.

a Downgraded due to risk of bias, inconsistency, lack of precision and potential publication bias.

**Table:** F Participants: Stroke survivors with visual neglect. Settings: Acute. Intervention: Substitution – eye patching. Reference standard: Control, standard or conservative care.

Outcome	*N* participants	Effect sham/standard care	Effect intervention	Significance between groups	Quality of evidence (GRADE)
**Change in visual neglect** Neuropsychological test accuracy^121^	Machner: 11 intervention 10 control	Change from baseline to 1 month Post-treatment versus Baseline: Mean 18.4 ± 6.0 *p* = 0.039 Follow-up versus Post-treatment: Mean 4.4 ± 3.7; *p* = 0.804	Change from baseline to 1 month Post-treatment versus Baseline: Mean 21.1 ± 4.2 *p* = 0.001 Follow-up versus Post-treatment: Mean 14.3 ± 4.0; *p* = 0.015	NS: *p* = 0.207	+ooo Very low^a^
Bells cancellation^128^ Figure copying^128^ Line bisection^128^	Aparicio-Lopez: 2017 13 intervention 18 Single therapy	Change from baseline to 12 weeks Pre: Mean 19.71 (SD 10.09) Post: Mean 28.43 (SD 4.11) *p* = 0.041 Pre: Mean 1.71 (SD 1.38) Post: Mean 1.43 (SD 1.13) *p* = 0.414 Pre: Mean −10.07 (SD 6.62) Post: Mean −9.74 (SD 4.04) *p* = 0.866	Change from baseline to 12 weeks Pre: Mean 11.14 (SD 7.26) Post: Mean 20.29 (SD 9.86) *p* = 0.028 Pre: Mean 3.29 (SD 1.49) Post: Mean 2.14 (SD 2.03) *p* = 0.157 Pre: Mean −7.59 (SD 7.99) Post: Mean −10.25 (SD 12.54) *p* = 0.176		
Bells cancellation^123^ Figure copying^123^ Line bisection^123^	Aparicio-Lopez: 2016 13 intervention 15 single therapy	Change from baseline to 5 days Pre: Mean 20.07 (SD 9.16) Post: Mean 29.67 (SD 3.83) *p* = 0.001 Pre: Mean 2.13 (SD 1.72) Post: Mean 0.60 (SD 0.98) *p* = 0.016 Pre: Mean 3.53 (SD 4.10) Post: Mean 0.93 (SD 1.38) *p* = 0.017	Change from baseline to 5 days Pre: Mean 14.08 (SD 9.35) Post: Mean 23.15 (SD 9.20) *p* = 0.003 Pre: Mean 2.46 (SD 1.89) Post: Mean 1.62 (SD 1.75) *p* = 0.131 Pre: Mean 5.23 (SD 4.64) Post: Mean 2.85 (SD 3.55) *p* = 0.066	NS: *p* = 0.856 NS: *p* = 0.496 NS: *p* = 0.892	
**Change in activities of daily living** Catherine Bergego scale^121^ Barthel index^121^	Machner: 11 intervention 10 control	Change from baseline to 1 month Post-treatment versus Baseline: Mean −4.2 ± 2.0; *p* = 0.184 Follow-up versus post-treatment: Mean −8.2 ± 1.8 *p* = 0.004 Post-treatment versus baseline: Mean 11.5 ± 3.0 *p* = 0.012 Follow-up versus post-treatment: Mean 14.0 ± 3.9 *p* = 0.017	Change from baseline to 1 month Post-treatment versus baseline: Mean −3.7 ± 1.9; *p* = 0.222 Follow-up versus post-treatment: Mean −9.0 ± 1.9 *p* = 0.002 Post-treatment versus baseline: Mean 20.9 ± 7.2 *p* = 0.047 Follow-up versus post-treatment: Mean 23.6 ± 8.0 *p* = 0.042	NS: *p* = 0.957 NS: *p* = 0.166	+ooo Very low^a^
Catherine Bergego scale^123^ Catherine Bergego scale^128^	Aparicio-Lopez: 13 intervention 15 single therapy Aparicio-Lopez:	Change from baseline to 5 days Pre: Mean 7.13 (SD 6.48) Post: Mean 6.14 (SD 6.13) *p* = 0.925 Change from baseline to 12 weeks Pre: Mean 10.08 (SD 7.29) Post: Mean 9.34 (SD 8.20) *p* = 0.753	Change from baseline to 5 days Pre: Mean 6.82 (SD 6.87) Post: Mean 4.40 (SD 3.37) *p* = 0.064 Change from baseline to 12 weeks Pre: Mean 9.35 (SD 8.49) Post: Mean 6.28 (SD 3.26) *p* = 0.236	NS: *p* = 0.254	

a Downgraded due to risk of bias, inconsistency, lack of precision and potential publication bias.

**Table:** G Participants: Stroke survivors with visual neglect. Settings: Acute. Intervention: Substitution – mirror therapy. Reference standard: Control, standard or conservative care.

Outcome	*N* participants	Effect sham/standard care	Effect intervention	Significance between groups	Quality of evidence (GRADE)
**Change in visual neglect** Behaviour inattention test^151^ Gap detection test^151^	Fong: 7 intervention 7 sham 1 7 sham 2	Change from baseline to 6 weeks Sham 1: Pre 86.57 (SD 43.70) Post 118.57 (SD 28.27) *p* = 0.005 Sham 2: Pre 58.86 (SD 43.73) Post 64.00 (SD 37.24) *p* = 0.623 Sham 1: Pre 16.43 (SD 14.11) Post 25.57 (SD 5.67) *p* = 0.021 Sham 2: Pre 12.36 (SD 11.95) Post 12.14 (SD 13.75) *p* = 0.954	Change from baseline to 6 weeks Pre 60.29 (SD 30.48) Post 93.00 (SD 23.81) *p* = 0.005 Pre 11.50 (SD 14.59) Post 20.50 (SD 10.95) *p* = 0.023	Intervention versus Sham 1 NS: *p* = 0.961 Intervention versus Sham 2 NS: *p* = 0.072 Intervention versus Sham 1 NS: *p* = 0.978 Intervention versus Sham 2 NS: *p* = 0.091	+ooo Very low^a^
Star cancellation^120^ Line bisection^120^ Picture identification task^120^	Pandian: 26 intervention 20 control	Change from baseline to 6 months Mean 12 Mean 10.4 Mean 2	Change from baseline to 6 months Mean 35 Mean 19 Mean 5.2	S: *p* < 0.0001 S: *p* = 0.001 S: *p* < 0.0001	
Star cancellation^153^ Line bisection^153^ Picture scanning^153^	Sim: 14 intervention 14 control	Change from baseline to 4 weeks Pre: Mean 46.64 ± 1.15 Post: Mean 50.64 ± 1.08 *p* = 0.001 Pre: Mean 16.84 ± 3.06 Post: Mean 8.85 ± 4.02 p=0.001 Pre: Mean 4.64 ± 1.44 Post: Mean 6.92 ± 1.15 *p* = 0.001	Change from baseline to 4 weeks Pre: Mean 46.86 ± 1.29 Post: Mean 51.78 ± 1.42 *p* = 0.001 Pre: Mean 16.19 ± 1.86 Post: Mean 5.86 ± 2.02 p=0.001 Pre: Mean 4.98 ± 1.29 Post: Mean 8.14 ± 1.16 *p* = 0.001	S: *p* = 0.02 S: *p* = 0.04 S: *p* = 0.02	
**Change in activities of daily living** Catherine Bergego scale^151^	Fong: 7 intervention 7 sham 1 7 sham 2	Change from baseline to 6 weeks Sham 1: Pre 15.17 (SD 9.00) Post 6.26 (SD 5.20) *p* = 0.001 Sham 2: Pre 15.68 (SD 9.45) Post 10.39 (SD 5.60) *p* = 0.024	Change from baseline to 6 weeks Pre 13.77 (SD 6.68) Post 8.58 (SD 8.62) *p* = 0.026	Intervention versus Sham 1 NS: *p* = 0.241 Intervention versus Sham 2 NS: *p* = 0.794	+ooo Very low^a^
Functional Independent Measure (FIM)^120^	Pandian: 26 intervention 20 control	Change from 1 to 6 months 1 month: 100% dependent 6 months: 95% dependent	Change from 1 to 6 months 1 month: 100% dependent 6 months: 58% dependent	1 month NS: *p* = 0.99 6 months S: *p* = 0.004	
Catherine Bergego scale^153^ Barthel index^153^	Sim: 14 intervention 14 control	Change from baseline to 4 weeks Pre: Mean 17.5 ± 4.18 Post: Mean 16.14 ± 3.41 *p* = 0.001 Pre: Mean 30.92 ± 11.57 Post: Mean 40.42 ± 11.14 *p* = 0.001	Change from baseline to 4 weeks Pre: Mean 17.85 ± 4.01 Post: Mean 13.07 ± 3.26 *p* = 0.001 Pre: Mean 35.64 ± 16.08 Post: Mean 48.14 ± 14.73 *p* = 0.001	S: *p* = 0.03 NS: *p* = 0.16	

a Downgraded due to risk of bias, inconsistency, lack of precision and potential publication bias.

**Table:** H Participants: Stroke survivors with visual neglect. Settings: Acute. Intervention: Restitution – brain stimulation. Reference standard: Control, standard or conservative care.

Outcome	*N* participants	Effect sham/standard care	Effect intervention	Significance between groups	Quality of evidence (GRADE)
**Change in visual neglect** Letter cancellation^126^	Kim: 15 intervention 19 sham	Change from pre- to post-treatment Pre: 14.42 ± 3.73 Post: 16.63 ± 3.24 *p* < 0.05	Change from pre- to post-treatment Pre: 11.87 ± 3.76 Post: 17.00 ± 2.85 *p* < 0.01	S: <0.01	+ooo Very low^a^
Line bisection^126^		Pre: 45.05 ± 7.83 Post: 39.26 ± 8.48 *p* < 0.05	Pre: 38.47 ± 16.9 Post: 14.45 ± 7.34 *p* < 0.05	S: <0.01	
Behaviour inattention test^125^	Yang: 20 intervention1 20 intervention2 20 control	Change from baseline to 6 weeks BIT-C: Pre; Mean 58.4 ± 31.0 Post; Mean 76.7 ± 33.2 *p* < 0.01	Change from baseline to 6 weeks (1) BIT-C: Pre; Mean 59.0 ± 35.3 Post; Mean 108.8 ± 27.1 *p* < 0.01 *p* < 0.01 (2) Change from baseline to 6 weeks BIT-C: Pre; Mean 56.0 ± 32.2 Post; Mean 99.0 ± 26.5 *p* < 0.01	S: *p* < 0.05	
Motor-free visual perception test^127^	Yi: 10 intervention1 10 intervention2 10 control	Change from baseline to 3 weeks Pre: Mean 8.3 ± 5.2 Post: Mean 10.3 ± 5.5 *p* < 0.05	Change from baseline to 3 weeks (1) Mean 6.8 ± 6.2 Post: Mean 12.0 ± 7.4 *p* < 0.05 (2) Mean 8.2 ± 6.8 Post: Mean 14.8 ± 5.8 *p* < 0.05	S: *p* = 0.043	
Star cancellation^127^		Pre: Mean 6.0 ± 4.8 Post: Mean 8.5 ± 4.9 *p* < 0.05	(1) Mean 6.2 ± 6.2 Post: Mean 13.3 ± 8.2 *p* < 0.05 (2) Mean 6.2 ± 6.6 Post: Mean 13.2 ± 8.5 p<0.05		
Line bisection^128^		Pre: Mean 26.0 ± 13.2 Post: Mean 19.0 ± 12.3 *p* < 0.05	1) Mean 26.8 ± 9.3 Post: Mean 12.4 ± 7.4 *p* < 0.05 (2) Mean 27.3 ± 18.6 Post: Mean 12.5 ± 13.0 *p* < 0.05		
Motor-free visual perception test^130^ Line bisection^130^ Star cancellation^130^ Albert’s test^130^	Kim: 10 intervention1 10 intervention2 10 control	Change from baseline to 2 weeks 11.7 ± 4.1 *p* < 0.05 −6.5 ± 4.1 *p* < 0.05 6.5 ± 2.2 *p* < 0.05 5.4 ± 2.5 *p* < 0.05	Change from baseline to 2 weeks (1) 13.8 ± 4.6 (2) 15.1 ± 3.07 *p* < 0.05 (1) −9.6 ± 2.6 (2) −10.8 ± 3.52 *p* < 0.05 (1) 7.9 ± 4.2 (2) 9.3 ± 2.1 *p* < 0.05 (1) 8.2 ± 1.2 (2) 5.4 ± 2.5 *p* < 0.05	NS: *p* = 0.085 NS: *p* = 0.098 NS: *p* = 0.125 NS: *p* = 0.077	
Composite neglect score^136^	Nyffeler: 10 intervention1 10 intervention2 10 sham	Change from baseline to discharge Mean change −0.2	Change from baseline to discharge (1) Mean change 0.8 (2) Mean change 0.4	S: *p* = 0.002	
Behaviour inattention test^150^	Da Silva: 15 intervention 1 15 intervention 2 16 control	Change from baseline to post-intervention Post: Mean difference 4.5 (95%CI −9.7 to 18.8) *p* = 0.99	Change from baseline to post-intervention (1) Post: Mean difference 18.4 (95%CI 3.9–32.8) *p* = 0.008 (2) Post: Mean difference 13.9 (95%CI −0.3 to 28.1) *p* = 0.057	S: *p* = 0.008 NS: *p* = 0.99	
Behaviour inattention test^139^	Iwanski: 13 intervention 14 control	Change from baseline to 3 months Pre: 81.9 ± 43.0 Post: 111 ± 30.1 *p* = 0.001	Change from baseline to 3 months Pre: 84.1 ± 41.0 Post: 112 ± 37.0 *p* = 0.001	NS: *p* = 0.87	
**Change in activities of daily living** Catherine Bergego scale^125^ Barthel index^125^	Yang: 20 intervention1 20 intervention2 20 control	Change from baseline to 6 weeks Pre: Mean 20.5 ± 5.8 Post; Mean 15.7 ± 6.6 *p* < 0.01 Pre: Mean 33.3 ± 18.4 Post: Mean 54.7 ± 23.2 *p* < 0.01	Change from baseline to 6 weeks (1) Pre: Mean 18.5 ± 6.8 Post: Mean 11.2 ± 6.4 *p* < 0.01 (2) Pre: Mean 21.2 ± 6.5 Post: Mean 13.9 ± 5.2 *p* < 0.01 (1) Pre: Mean 33.3 ± 16.6 Post: Mean 60.7 ± 17.0 *p* < 0.01 (2) Pre: Mean 26.7 ± 10.8 Post: Mean 55.2 ± 17.1 *p* < 0.01	NS: *p* = 0.18 NS: *p* = 0.07 NS: *p* = 0.46 NS: *p* = 0.56	+ooo Very low^a^
Functional independence measure^126^	Kim: 15 intervention 15 control	Change from baseline to 6 weeks Pre: Mean 65.4 ± 11.4 Post: Mean 68.3 ± 18.4 *p* < 0.05	Change from baseline to 6 weeks Pre: Mean 66.8 ± 9.5 Post: Mean 79.57 ± 11.3 *p* < 0.05		
Catherine Bergego scale^127^ Barthel index^127^	Yi: 10 intervention1 10 intervention2 10 control	Change from baseline to 3 weeks Pre: Mean 16.0 ± 9.7 Post: Mean 12.3 ± 10.8 *p* < 0.05 Pre: Mean 22.1 ± 15.8 Post: Mean 36.8 ± 13.3 *p* < 0.05	Change from baseline to 3 weeks (1) Pre: Mean 17.0 ± 10.6 Post: Mean 8.4 ± 9.0 *p* < 0.05 (2) Pre: Mean 16.2 ± 6.4 Post: Mean 10.0 ± 6.2 *p* < 0.05 (1) Mean 19.1 ± 11.8 Post: Mean 42.1 ± 21.3 *p* < 0.05 (2) Mean 22.5 ± 12.2 Post: Mean 46.0 ± 20.5 *p* < 0.05	NS: *p* = 0.132 NS: *p* = 0.305 NS: *p* = 0.152 NS: *p* = 0.870	
Catherine Bergego scale^130^ Barthel index^130^	Kim: 10 intervention1 10 intervention2 10 control	Change from baseline to 2 weeks −7.1 ± 3.0 *p* < 0.05 4.5 ± 5.1 *p* < 0.05	Change from baseline to 2 weeks (1) −7.5 ± 2.3 (2) −9.2 ± 1.4 *p* < 0.05 (1) 15.0 ± 6.7 (2) 15.4 ± 5.41 *p* < 0.05	S: *p* = 0.004 S: *p* = 0.00453	
Catherine Bergego scale^136^ Functional Independence measure^136^	Nyffeler: 10 Intervention1 10 intervention2 10 sham	Change from baseline to discharge Mean change 5 Mean change 15	Change from baseline to discharge (1) Mean change 7.5 (2) Mean change 11 (1) Mean change 24 (2) Mean change 28		
Catherine Bergego Scale^150^ Functional Independence Measure^150^ Barthel Index^150^	Da Silva: 15 intervention 1 15 intervention 2 16 control	Change from baseline to post-intervention Post: Mean difference 0.6 (95%CI −3.8 to 5.2) *p* = 0.93 Post: Mean difference −1.8 (95%CI −14.5 to 10.9) *p* = 0.93 Post: Mean difference 1.0 (95%CI −14.5 to 16.5) *p* = 0.98	Change from baseline to post-intervention (1) Post: Mean difference 0.06 (95%CI −4.5 to 4.6) *p* = 0.99 (2) Post: Mean difference 0.7 (95%CI −3.8 to 5.2) *p* = 0.91 (1) Post: Mean difference −4.0 (95%CI −16.7 to 8.7) *p* = 0.90 (2) Post: Mean difference −2.2 (95%CI −14.9 to 10.5) *p* = 0.73 (1) Post: Mean difference −5.3 (95%CI −20.8 to 10.2) *p* = 0.68 (2) Post: Mean difference −4.3 (95%CI −19.8 to 11.2) *p* = 0.77	NS: *p* = 0.99 NS: *p* = 0.93 NS: *p* = 0.90 NS: *p* = 0.93 NS: *p* = 0.68 NS: *p* = 0.98	
Functional independence measure and Functional adaptation measure^139^	Iwanski: 13 intervention 14 control	Change from baseline to 3 months Pre: Mean 113.5 ± 27.0 Post: Mean ± 137.0 ± 27.0 *p* = 0.001	Change from baseline to 3 months Pre: Mean 124.4 ± 46.0 Post: Mean ± 143.5 ± 41.0 *p* = 0.001	NS: *p* = 0.65	

BIT-C: Behavioral Inattention Test-conventional subtest.

a Downgraded due to risk of bias, inconsistency, lack of precision and potential publication bias.

Generally, the rehabilitation approaches for hemispatial neglect include a combination of restorative (e.g. computerised training, brain stimulation), substitutive (e.g. hemifield eye patching, optokinetic stimulation) and/or compensatory training (e.g. visual scanning, visuomotor training). Studies addressing computerised training, including eye patching and virtual reality/cognitive training, included four trials.^123,128,140,143^ Eleven trials, three cohort and one case control study evaluated prism adaptation^129,132,134,141,146,148,149,152,154,156–158,160,161,162^ Robot-assisted training was evaluated by five trials.^124,131,135,142,145^ The effects of brain stimulation therapy on visual neglect were evaluated by eight trials.^125–127,130,136,139,147,150,163^ Machner et al. and Aparicio-López et al. evaluated the effects of eye patching,^121,128^ while Fong et al., Pandian et al. and Sim et al. evaluated the use of mirror therapy.^120,151,153^ Visual scanning training and/or visuomotor training was evaluated in six trials and one cohort study.^119,122,133,137,144,155^ Further, two studies evaluated the impact of exercise on neglect outcomes.^138,159^

### Additional information

Besides the lack of consistency in the outcome variables, there was considerable heterogeneity in the effectiveness of interventions for visual neglect across studies. Some interventions such as prism adaptation and certain types of visual scanning training showed promise in improving activities of daily living and quality of life in stroke survivors with visual neglect. Other interventions showed improvement in activities of daily living, for both the intervention and control groups, but with no greater significance for the intervention in comparison to the control therapy; for example, computerised training and robot-assisted training. Additional interventions such as mirror therapy and brain stimulation techniques varied across studies and showed effect of intervention for change in neglect measures but not for activities of daily living. Eye patching did not show any significant change in neglect or daily life measures. Generally, there is a lack of high-quality RCTs suggesting that treatments for visual neglect are effective in improving activities of daily living. We agree with previous systematic reviews that the effectiveness of interventions for visual neglect remains unproven and, no rehabilitation approach can be supported or refuted based on the current evidence.^21^


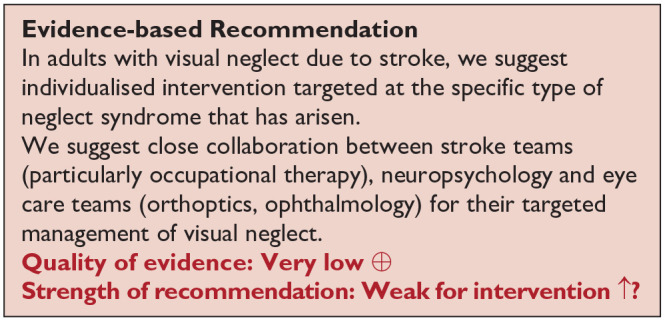


***PICO 13:* For adults with other visual perceptual disorders due to stroke, does compensatory, substitute or restitutive intervention, compared to no intervention, improve activities and quality of daily life?**

### Analysis of current evidence

In this PICO, we considered whether compensatory, substitute or restitutive interventions can improve activities and quality of daily life in stroke patients with visual perceptual disorders. For the purpose of the present guidelines, we define compensatory, substitutive and restitutive interventions as treatment options to improve adaptation to the impairment (compensatory, e.g. visual scanning, blinking), to improve the visual impairment using a device or optical aid (substitutive, e.g. magnifier), and to restore visual perception (restitution, e.g. pharmacological intervention). We considered all steps in the stroke pathway.

For this PICO, we included outcomes that were rated as critical by the writing group. These included change in visual perception, activities of daily living and quality of life. We found four RCTs (Supplemental Table 17) comparing interventions for visual perceptual disorders compared to sham intervention, with a mean sample size of 32 (SD 8.5; median 30, range 24–44).^126,164–166^ Two included compensatory interventions^164,166^ and two addressed the effects of transcranial direct current stimulation (tDCS) or repetitive transcranial magnetic stimulation (rTMS).^126,163,165^ All four trials reported on changes in visual perception and activities of daily living.^126,164–166^ One trial reported additionally on changes to quality of life.^165^

**Figure 13.**
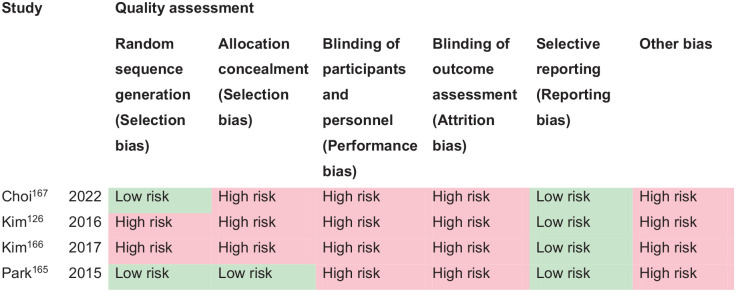
PICO 13 – Risk of bias assessment.

Table 13.1 and Figure 13 show the GRADE assessment of interventions for visual perceptual disorders. Meta analysis was not possible due to considerable heterogeneity across included trials with different interventions, outcome measures and timing of treatment post stroke. Most studies had a high risk of bias. Limitations included study heterogeneity, unblinded interpretation of test results and limited information on complete or missing data.

**Table 13.1.** PICO 13 – For adults with other visual perceptual disorders due to stroke, does compensatory, substitute or restitutive intervention, compared to no intervention, improve activities and quality of daily life? Summary of findings for PICO 13. Assessment of the interventions for visual perceptual disorders due to stroke. A Participants: Stroke survivors with visual perceptual disorders. Settings: Acute. Intervention: Compensatory. Reference standard: Control, standard or conservative care.

Outcome	*N* participants	Effect sham/standard care	Effect intervention	Significance between groups	Quality of evidence (GRADE)
**Change in visual perception** Motor-free visual perception test^166^	Choi: 12 intervention 12 standard care	Change from baseline to 6 weeks Pre: Mean 28.3 ± 1.3 Post: Mean 31.7 ± 1.9 *p* < 0.001	Change from baseline to 6 weeks Pre: Mean 27.8 ± 2.0 Post: Mean 32.7 ± 2.5 *p* < 0.001	NS: *p* = 0.735	++oo Low^a^
Motor-free visual perception test^164^	Park: 15 intervention 15 standard care	Change from baseline to 4 weeks Mean 2.5 (SD 1.7) <0.05	Change from baseline to 4 weeks Mean 6.6 (SD 0.5) *p* < 0.05	*p* < 0.05	
**Change in activities of daily living** Korean modified Barthel index^166^	Choi: 12 intervention 12 standard care	Change from baseline to 6 weeks Pre: Mean 59.2 ± 14.5 Post: Mean 72.5 ± 12.6 *p* < 0.001	Change from baseline to 6 weeks Pre: Mean 55.9 ± 14.4 Post: Mean 80.9 ± 12.3 *p* < 0.001	NS: *p* = 0.15	++oo Low^a^
Lowenstein occupational therapy cognitive assessment^164^	Park: 15 intervention 15 standard care	Change from baseline to 4 weeks Mean 5.3 (SD 2.3) *p* < 0.05	Change from baseline to 4 weeks Mean 14.4 (SD 2.0) *p* < 0.05	*p* < 0.05	

a Downgraded due to high risk of bias and potential publication bias.

**Table:** B Participants: Stroke survivors with visual perceptual disorders. Settings: Acute. Intervention: Restitutive. Reference standard: Control, standard or conservative care.

Outcome	*N* participants	Effect sham/standard care	Effect intervention	Significance between groups	Quality of evidence (GRADE)
**Change in visual perception** Motor-free visual perception test^126^	Kim: 15 intervention 15 sham	Change from baseline to 6 weeks Pre: Mean 21.0 ± 3.9 Post: Mean 23.9 ± 3.8 *p* < 0.05	Change from baseline to 6 weeks Pre: Mean 21.1 ± 3.6 Post: Mean 26.8 ± 3.1 *p* < 0.05	N/A	++oo Low^a^
Motor-free visual perception test^166^	Kim: 22 intervention 22 sham	Change from baseline to 8 weeks Pre: Mean 15.6 ± 4.4 Post: Mean 21.4 ± 5.1 *p* < 0.05	Change from baseline to 8 weeks Pre: Mean 14.9 ± 6.2 Post: Mean 29.0 ± 5.3 *p* < 0.05		
**Change in activities of daily living** Functional independence measure^126^	Kim: 15 intervention 15 sham	Change from baseline to 6 weeks Pre: Mean 65.4 ± 11.4 Post: Mean 68.3 ± 18.4 *p* < 0.05	Change from baseline to 6 weeks Pre: Mean 66.8 ± 9.5 Post: Mean 79.57 ± 11.3 *p* < 0.05	N/A	++oo Low^a^
Functional independence measure^165^	Kim: 22 intervention 22 sham	Change from baseline to 8 weeks Pre: Mean 67.2 ± 7.9 Post: Mean 71.3 ± 7.6 *p* > 0.05	Change from baseline to 8 weeks Pre: Mean 66.6 ± 7.8 Post: Mean 79.6 ± 6.4 *p* < 0.05		
**Change in quality of life** Beck depression inventory^165^	Kim: 22 intervention 22 sham	Change from baseline to 8 weeks Pre: Mean 25.5 ± 1.9 Post: Mean 22.3 ± 5.1 *p* > 0.05	Change from baseline to 8 weeks Pre: Mean 25.8 ± 2.3 Post: Mean 15.5 ± 3.3 *p* > 0.05		++oo Low^a^

a Downgraded due to high risk of bias and potential publication bias.

### Additional information

The evidence suggests limited application of task training (e.g. Nintendo games) for acute stroke survivors due to the lack of practicality in acute care settings. However, this could be a useful approach for chronic stroke survivors with visual perceptual disorders. Similarly, the practicalities of early intervention with rTMS/tDCS warrant further study. Any form of intervention seemed to have a positive effect on activities of daily living whether improvement was from the intervention being studied or interventions/activities undertaken by the control group. There was insufficient evidence to report on change to quality of life.

Overall, there remains insufficient evidence due to difficulties accessing adequate numbers of stroke survivors with visual perceptual disorders. This is due primarily to the considerable heterogeneity that exists for type of visual perceptual disorders. There are not enough numbers of specific types of visual perceptual disorders to power significance for an intervention trial and, to achieve this, large-scale collaboration is required from multiple recruitment sites.^20^ Clinicians should signpost to appropriate vision information and resource materials. There are a variety of free information resources available internationally.^95^


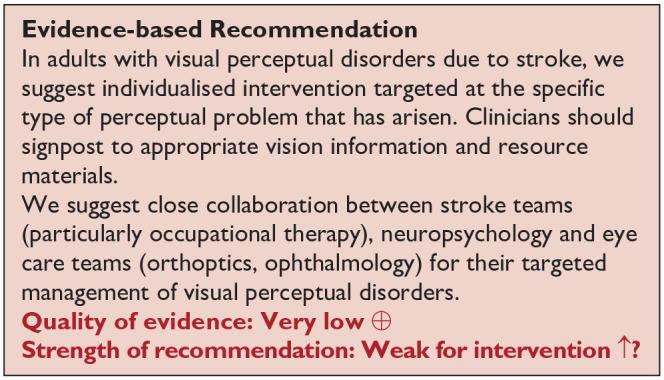


## Discussion

This guideline document was developed following the GRADE methodology and aims to assist physicians in decision-making regarding visual impairment resulting from stroke. All recommendations and expert consensus statements are summarised in Table 14 and Figure 14.

**Table 14.** Vision guideline recommendations and expert consensus statements.

Recommendation	Expert consensus statement
PICO 1 for adults with visual problems due to stroke, does routine use of vision screening, compared to no routine vision screening, improve detection rate?	
In adults with stroke, we suggest vision screening to improve detection of visual problems. Vision screening should be undertaken using a validated vision screening tool or by specialist eye team assessment. Vision screening versus routine stroke screening improves the detection rate of presence of visual impairment while specialist visual assessment further improves the accuracy of detection of visual impairment. Quality of evidence: QUADAS-2 **Medium risk of bias** 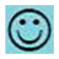 Strength of recommendation: **Weak for intervention ↑?**	
PICO 2 for adults with visual problems due to stroke, does early assessment within 1 week of stroke admission, compared to later assessment, improve activities and quality of daily life?	
For adults with visual problems due to stroke, there are insufficient data to make an evidence-based recommendation on the use of early vision screening. Please see the expert consensus statement to the right. Quality of evidence: **Low ⊕⊕** Strength of recommendation: **Not assessable (–)**	10 of 10 experts suggest: 1. In adults with stroke, early vision screening should be undertaken to detect their visual problems. This is feasible and acceptable within 3–4 days post onset of stroke. The majority can be assessed within 1 week post-stroke onset. 2. Vision screening should be undertaken by specialist eye team assessment or at least by using a validated vision screening tool.
PICO 3 for adults with visual field loss due to stroke, does identification of visual field loss by vision screening or specialist eye team, compared to routine stroke screen, improve detection rate and activities/quality of life?	
For adults with visual field loss due to stroke, there are insufficient data to make an evidence-based recommendation on the use of vision screening or specialist eye team assessment compared to routine stroke screen. Please see the expert consensus statement to the right. Quality of evidence: QUADAS-2 **Medium risk of bias** 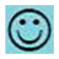 Strength of recommendation: **Not assessable (–)**	10 of 10 experts suggest: 1. In adults with stroke, early vision screening should be undertaken to detect visual field loss. This is feasible and acceptable within 3–4 days post onset of stroke. The majority can be assessed within 1 week post onset. Visual field loss screening should be undertaken by specialist eye team assessment or at least by using a validated vision screening tool. 2. Vision screening versus routine stroke screening improves the detection rate of presence of visual field loss while specialist visual assessment further improves the accuracy of detection of visual impairment.
PICO 4 for adults with central vision impairment due to stroke, does identification of visual acuity loss by vision screening or specialist eye team, compared to routine stroke screen, improve detection rate and activities/quality of life?	
For adults with central vision impairment due to stroke, there are insufficient data to make an evidence-based recommendation on the use of vision screening or specialist eye team assessment compared to routine stroke screen. Please see the expert consensus statement to the right. Quality of evidence: QUADAS-2 **Medium risk of bias** 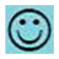 Strength of recommendation: **Not assessable (–)**	10 of 10 experts suggest: 1. In adults with stroke, early vision screening should be undertaken to detect central vision impairment. This is feasible and acceptable within 3–4 days post onset of stroke. The majority can be assessed within 1 week post onset. Visual acuity loss screening should be undertaken by specialist eye team assessment or at least by using a validated vision screening tool. 2. Vision screening versus routine stroke screening improves the detection rate of presence of central vision impairment while specialist visual assessment further improves the accuracy of detection of visual impairment.
PICO 5 for adults with eye movement disorders due to stroke, does identification of strabismus and/or ocular motility deficit loss by vision screening or specialist eye team, compared to routine stroke screen, improve detection rate and activities/quality of life?	
For adults with eye movement disorders due to stroke, there are insufficient data to make an evidence-based recommendation on the use of vision screening or specialist eye team assessment compared to routine stroke screen. Please see the expert consensus statement to the right. Quality of evidence: QUADAS-2 **Medium risk of bias** 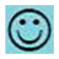 Strength of recommendation: **Not assessable (–)**	10 of 10 experts suggest: 1. In adults with stroke, early vision screening should be undertaken to detect eye movement disorders. This is feasible and acceptable within 3–4 days post onset of stroke. The majority can be assessed within 1 week post onset. Screening for eye movement disorders should be undertaken by specialist eye team assessment or at least by using a validated vision screening tool. 2. Vision screening versus routine stroke screening improves the detection rate of presence of eye movement disorders while specialist visual assessment further improves the accuracy of detection of visual impairment.
PICO 6 for adults with visual perceptual disorders due to stroke, does identification of visual perceptual disorders by screening proforma/tool or specialist team, compared to routine stroke screen, improve detection rate and activities/quality of life?	
For adults with visual perceptual disorders due to stroke, there are insufficient data to make an evidence-based recommendation on the use of vision screening or specialist eye team assessment compared to routine stroke screen. Please see the expert consensus statement to the right. Quality of evidence: QUADAS-2 **Medium risk of bias** 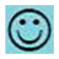 Strength of recommendation: **Not assessable (–)**	10 of 10 experts suggest: 1. In adults with stroke, early vision screening should be undertaken to detect visual perceptual disorders. This is feasible and acceptable within 3–4 days post onset of stroke. The majority can be assessed within 1 week post onset. Screening for visual perceptual disorders should be undertaken by specialist eye team assessment or at least by using a validated vision screening tool. 2. Vision screening versus routine stroke screening improves the detection rate of presence of visual perceptual disorders while specialist visual assessment further improves the accuracy of detection of visual impairment.
PICO 7 for adults with visual neglect due to stroke, does identification of visual neglect by screening proforma/tool or specialist team, compared to routine stroke screen, improve detection rate and activities/quality of life?	
In adults with stroke, we suggest vision screening to improve detection of visual neglect. Vision screening versus routine stroke screening improves the detection rate of presence of visual neglect while specialist visual assessment and use of a battery of tests, further improves the accuracy of detection of visual neglect. Quality of evidence: QUADAS-2 **High risk of bias** Strength of recommendation: **Weak for intervention** ↑?	
PICO 8 for adults with homonymous visual field loss due to stroke, does compensatory, substitute or restitutive intervention, compared to no intervention, improve activities and quality of daily life?	
In adults with visual field loss due to stroke, we suggest compensatory interventions of visual scanning/visual search to aid adaptation to visual field loss after stroke. We suggest early commencement of treatment as soon as is feasible and acceptable to the patient. Quality of evidence: **Very low ⊕** Strength of recommendation: **Weak for intervention** ↑?	
PICO 9 for adults with ocular stroke, does compensatory, substitute or restitutive intervention, compared to no intervention, improve activities and quality of daily life?	
In adults with ocular stroke (central retinal artery occlusion), we suggest thrombolysis within 4.5 h of stroke onset to aid recovery of visual function after stroke. Quality of evidence: **Very low ⊕** Strength of recommendation: **Weak for intervention** ↑?	
PICO 10 For adults with central vision impairment due to stroke, does compensatory, substitute or restitutive intervention, compared to no intervention, improve activities and quality of daily life?	
For adults with central vision impairment due to stroke, there are insufficient data to make an evidence-based recommendation on the use of compensatory, substitutive or restitutive interventions. Please see the expert consensus statement to the right. Quality of evidence: **Very low ⊕** Strength of recommendation: **Not assessable (–)**	10 of 10 experts suggest: 1. For adults with central vision impairment due to stroke, early management options to improve visual acuity should be offered as soon as possible after stroke onset. 2. Stroke survivors should wear their prescribed spectacles and these should be updated promptly if lost/broken or old. Clinicians should signpost to appropriate information, resource materials and vision aids.
PICO 11 for adults with eye movement disorders due to stroke, does compensatory, substitute or restitutive intervention, compared to no intervention, improve activities and quality of daily life?	
In adults with eye movement disorders due to stroke, we suggest individualised intervention targeted at the specific type of eye movement problem that has arisen. We suggest referral to specialist eye services for the targeted management of eye movement disorders. Prior to/while awaiting specialist eye care, alleviation of troublesome diplopia can be achieved using partial or total occlusion (eye patching) with a caveat of caution where total occlusion is used, thus rendering the patient monocular with potential impact from loss of visual input from the occluded eye. Quality of evidence: **Very low ⊕** Strength of recommendation: **Weak for intervention** ↑?	
PICO 12 for adults with visual neglect due to stroke, does compensatory, substitute or restitutive intervention, compared to no intervention, improve activities and quality of daily life?	
In adults with visual neglect due to stroke, we suggest individualised intervention targeted at the specific type of neglect syndrome that has arisen. We suggest close collaboration between stroke teams (particularly occupational therapy), neuropsychology and eye care teams (orthoptics, ophthalmology) for their targeted management of visual neglect. Quality of evidence: **Very low ⊕** Strength of recommendation: **Weak for intervention ↑?**	
PICO 13 for adults with other visual perceptual disorders due to stroke, does compensatory, substitute or restitutive intervention, compared to no intervention, improve activities and quality of daily life?	
In adults with visual perceptual disorders due to stroke, we suggest individualised intervention targeted at the specific type of perceptual problem that has arisen. Clinicians should signpost to appropriate vision information and resource materials. We suggest close collaboration between stroke teams (particularly occupational therapy), neuropsychology and eye care teams (orthoptics, ophthalmology) for their targeted management of visual perceptual disorders. Quality of evidence: **Very low ⊕** Strength of recommendation: **Weak for intervention ↑?**	

**Figure 14.**
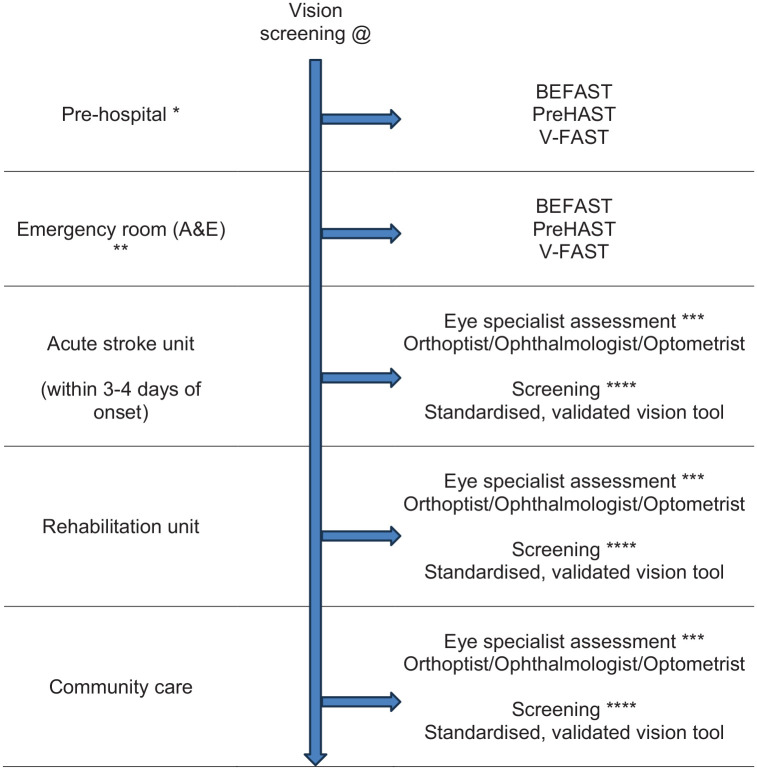
Summary recommendations. A: Diagnosis. *Rapid vision checklist (<5 min duration) as an adjunct to FAST to aid decision making – is this a stroke? **Rapid vision checklist (<5 min duration) as an adjunct to FAST and NIHSS to aid decision making – is this a stroke? ***Ideally, vision assessment for all stroke survivors undertaken by a member of the eye team – does this stroke survivor have a visual problem? Achieves accurate diagnosis rapidly, allowing prompt early management of visual impairment. ****Where limited/no access to eye specialist assessment, vision screening undertaken by a member of the stroke multi-disciplinary team. Use of a standardised and validated vision screening tool (<30 min duration) facilitates detection of visual impairment across main types of visual impairment occurring after stroke, allows test-retest and facilitates triage of referrals.

**Figure 14.**
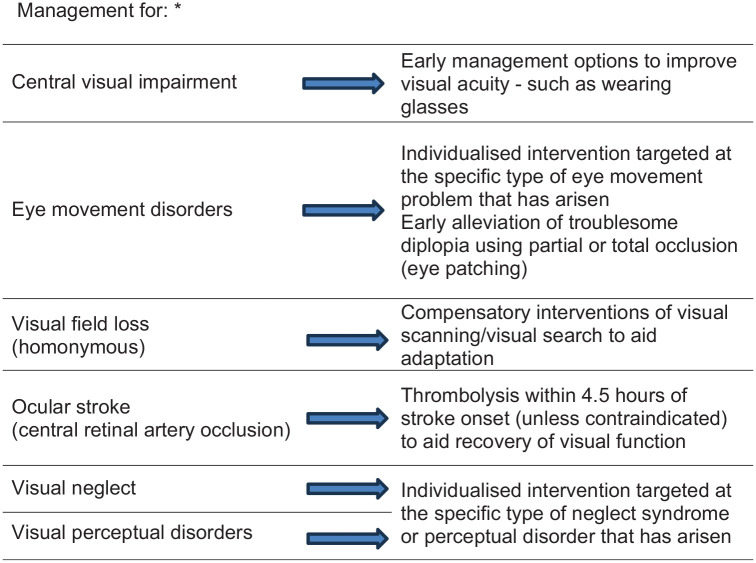
Summary recommendations. B: Management. * Management options for visual impairment should be offered as soon as possible after stroke onset, i.e. within days of stroke onset. This maximises residual visual function to promote best engagement with stroke rehabilitation. Close collaboration between stroke teams (particularly occupational therapy), neuropsychology and eye care teams (orthoptics, ophthalmology) is ideal. Clinicians should provide appropriate vision-specific information, resource materials and vision aids.

Visual problems are common in stroke survivors with prevalence of about 75% and incidence of about 60%.^2^ Rowe and colleagues identified impaired central visual acuity in 56%, eye movement abnormalities in 40%, visual field loss in 28%, visual inattention in 27% and visual perceptual disorders in 5%.^1^ Stroke survivors with visual impairment do not all receive good assessment or management of their visual disorders, with the more disadvantaged being females, black ethnicity, lower socioeconomic status, older age and those with lower education attainment.^12^

Stroke can also affect a person’s ability to interpret and integrate information received from their senses, including vision. This is of vital importance, given that an estimated 70% of all sensory information we perceive is visual.^167^ Recognising visual problems following stroke is important, as their presence is negatively correlated with rehabilitation and quality of life.^13,168^ Any type of visual impairment has the potential to affect quality of life and activities of daily life such as mobility and navigation, social interaction, self-care, independence, mood and depression. One significant effect of stroke-related visual impairment is the impact to driving ability. It is important that vision screening encompasses measures of visual acuity, visual field, visual neglect and eye movements to ensure no-one with visual impairment sufficient to breach driving regulations (which may vary from country to country) is discharged without appropriate information specific to their driving ability. Further, follow-up is required to review their visual requirements. Appropriate treatment (e.g. prisms or patch for double vision) may allow stroke survivors to regain appropriate levels of visual function required for driving.

Since there is currently no standardised protocol for the detection of visual disorders in stroke patients, they may go undetected, resulting in poor self-care,^169^ inability to perform activities of daily living^67^ and reduced quality of life.^170^ Significant inequalities exist for stroke-related visual impairment.^4^ Considerable variability occurs for the way in which vision screening is, or is not, provided, along with access to referral to specialist eye services, management of visual impairment, and provision of appropriate information. This causes considerable health inequalities and unmet need with poor patient experience of stroke vision care, lack of personalised approach to vision rehabilitation with lack of adapted communication needs appropriate to those who are visually impaired.

A further issue relates to the self-reports of visual symptoms by stroke survivors. Norup et al. identified visual problems on the initial neurologic examination in 24% of acute stroke survivors.^52^ Of those that declined further evaluation, the reason given was that they were not aware of visual problems. In stroke survivors with visual neglect, lack of awareness of their visuospatial deficits was the most important predictor of poorer performance in activities of daily life and this seemed more important than the severity of the deficits and, than the time post-stroke.^171^ In fact, 40–60% of stroke survivors with new onset visual impairment do not or cannot report visual symptoms and some seemed unaware of the impact of this deficit in their daily lives despite caregivers reporting frequent collisions and accidents.^29,46^ This poses a dilemma, as these patients risk not receiving adequate rehabilitation if missed. Additionally, patients who are unaware of their visual field loss may continue daily activities such as driving, and possibly pose significant safety issues on road safety.

We therefore need to strive to improve vision services for acute stroke care. In clinical practice, different professionals involved in stroke care may use different assessment tools/batteries for visual and perceptual impairment, with varying degrees of validation/normative data, construct validity, selection appropriateness and so on.^4,37,48^ These tools may not be widely shared if not included in clinical trials/studies. Therefore, there may be a discrepancy between this and other reviews of the scientific literature and the reality of clinical practice. The design, evaluation and validation of reliable screening methods to detect visual disorders after stroke is necessary for the subsequent development and implementation of early rehabilitation interventions. In this regard, we searched for screening methods to detect visual disorders after stroke in general, as well as specific deficits of central or peripheral visual field, eye movements and visual neglect and perception. Despite a general lack of high-quality research, there was consistent evidence that early vision screening was both feasible and acceptable and does improve detection of visual impairment with strong potential to increase accuracy of diagnosis, facilitate timely referrals and access to visual rehabilitation.

Hence, because of the potential implications on diagnosis, treatment and rehabilitation, early vision screening is to be recommended. The use of vision screening versus specialist vision assessment will differ across countries. Some will and some will not have access to specialist vision assessment on stroke units. Ideally, where this is possible, then specialist vision assessment is recommended to provide more robust assessment with accurate diagnosis and access to prompt management at the time of contact. If not, we recommend use of vision screening tools, which are designed to allow screening to be undertaken by any member of the stroke multi-disciplinary team. To this regard it is important that fast and accurate screening tools are utilised to assess all potential post-stroke visual impairments.^7^ First, there are several appropriate vision checklists that can be recommended for pre-hospital and emergency room use as adjuncts to FAST and NIHSS. These are rapid checks taking less than 5 min to complete which aid decision-making to aid stroke detection and, in particular, posterior circulation stroke. Further, there are several appropriate vision screening tools that can be recommended for acute in-patient use for deficits of central and peripheral vision and visual field, and for eye movements. These take between 10 and 30 min for completion, are designed for bedside testing and for testing with stroke survivors with communication or mild cognition issues, and can be used by any member of the stroke multi-disciplinary team. For general vision screening, and timing of such screening, using standardised, validated vision screening improves detection of visual impairment in stroke survivors, allowing prompt management and better engagement with therapy/rehabilitation, and with potential to improve quality of life and daily life activities. Vision screening can take place as early as pre-hospital settings but is eminently feasible at hyperacute/acute stroke care settings within 3–4 days of stroke onset, where diagnosis of visual impairment is a crucial component of overall assessment and subsequent care. For stroke survivors who are not initially able to undertake vision screening, later vision screening should be offered once they improve sufficiently to comply with vision screening.

For vision screening of impaired central and peripheral vision/visual fields and eye movement disorders, early screening increases the detection rate with high sensitivity and specificity of freely available options such as the Vision Impairment Screening Assessment (VISA) tool. Screening with a standardised validated tool is optimal as screening checklists, such as the NIH stroke scale for severity, do not test visual acuity, eye movements in all gaze positions, visual field loss other than hemianopia, and visual perceptual disorders other than neglect. For neglect and visual perceptual disorders, no single test alone has been found to be sufficient to exclude neglect. Although a cancellation test has been suggested as a quick primary screening test,^8^ multiple neglect tests should be attempted. We support the recommendations from The European Academy of Neurology that one or more of line bisection, figure copying, and baking tray task should be added to a cancellation test.^8^ Further, they recommend the Catherine Beregeo Scale for functional and ecological testing.

It should also be noted that presence of cognitive and/or communication impairment in stroke survivors is not a deterrent for vision screening. In many tests of visual function the individual can indicate by hand signal their response to presence/absence or yes/no questions. Further, eye care specialists have a wealth of experience and alternative testing options for assessing visual function of babies and young children; such options are readily utilised for adults with severe cognitive impairment.

Where visual impairment is the only presenting sign of a stroke, recognition of this and for stroke being the underlying cause, allows immediate referral to a stroke unit with important therapeutic consequences, such as being able to offer reperfusion therapy within the therapeutic window of time.^172^ There are no predictive factors for those who will recover fully, partially or not at all regarding their visual impairment. All can benefit from prescription of accurate spectacles, from rehabilitation measures such as prisms, from learning coping strategies^168^ and even from simple magnification while waiting to determine if (or not) visual recovery will occur.^44^

Evidence for interventions for visual rehabilitation was variable dependent on the type of visual impairment. We included interventions for central retinal artery occlusion as it is important to highlight this ocular branch of stroke. Like brain stroke, evidence points to timely (within 4.5 h) thrombolysis in improving visual function outcomes, especially visual acuity. However, this requires close collaboration with stroke/neurology/ophthalmology/primary care teams. Further guidelines on reperfusion therapy can be found in the ESO guideline on this topic. For visual field loss, the greatest indication of treatment benefit was compensatory interventions such as visual scanning or search training with improvement for activities of daily living. Early treatment facilitates a build of compensation and adaptation.

For other visual impairments such as reduced visual acuity and eye movement disorders, although there was a lack of stroke population specific research, there was considerable research evidence for effectiveness of interventions in similar visual impairments caused by other forms of acquired brain injury. Hence, evidence does exist and close liaison with eye care specialists is needed to ensure access to the knowledge base for appropriate management of the varying types of visual impairment that occur following stroke. Conversely, evidence was limited for interventions for visual neglect and visual perceptual disorders and further research is needed here along with close collaboration with neuropsychology.

Future research is needed as high quality diagnostic accuracy studies and interventional randomised controlled trials are lacking. These require appropriately powered sample sizes to ensure both clinical and statistical significance alongside evaluation of cost effectiveness. A focus is needed on utilising core outcome sets and core outcome measures with inclusion of both objective and subjective outcome measures to document change in the visual impairment alongside change to quality of life and daily life activities over appropriate follow-up periods.^173^ Research is needed on the impact to activities of daily living and quality of life from early visual assessment and impact on measures such as length of stay. Further, research is needed to investigate how visual sensitivity and discrimination awareness (blindsight) can improve outcomes for those with visual field loss, and how interventions can impact falls rates and driving performance. We await the outcomes of current on-going trials, as outlined in individual PICOs above. We further advise consideration of outcomes and recommendations of previous systematic reviews (outlined in our methods) that underpinned our searches for this ESO Vision Guideline.

This ESO guideline on visual impairment in stroke represents the currently available scientific data. Refinement and revision of some of these recommendations may be considered whenever further scientific-based data becomes available.

## Supplemental Material

sj-docx-1-eso-10.1177_23969873251314693 – Supplemental material for European Stroke Organisation (ESO) guideline on visual impairment in stroke Supplemental material, sj-docx-1-eso-10.1177_23969873251314693 for European Stroke Organisation (ESO) guideline on visual impairment in stroke by Fiona J Rowe, Lauren R Hepworth, María Begoña Coco-Martin, Celine R Gillebert, Luis Leal-Vega, Anja Palmowski-Wolfe, Eleni Papageorgiou, Stephen James Ryan, Karolina Skorkovska and Anne Hege Aamodt in European Stroke Journal
