# Supplementary material for: European Stroke Organisation (ESO) guideline on visual impairment in stroke
Source: Eur Stroke J. 2025 May 22;10(4):1087–159. doi: 10.1177/23969873251314693 (PMC12098360; doi:10.1177/23969873251314693)
Supplement: sj-docx-1-eso-10.1177_23969873251314693 – Supplemental material for European Stroke Organisation (ESO) guideline on visual impairment in stroke [file sj-docx-1-eso-10.1177_23969873251314693.docx]

**Supplementary table 1; Conflicts of interest of module working group members**

| **Author** | **Discipline and affiliation** | **Intellectual and financial disclosures** |
| --- | --- | --- |
| Fiona Rowe | Professor of Orthoptics, Head of Department, University of Liverpool | Intellectual COI:  Guideline and stroke/vision committees:  1, Member of BIOS stroke and neuro-rehab Clinical Advisory Group.  2, Member of UK intercollegiate stroke working party and guideline development group for UK National Clinical Guidelines for Stroke.  3, Co-opted topic advisor (Orthoptics) on NICE stroke rehab guidelines for adults.  4, Cochrane editor and author - Eyes and Vision group    Relationships with industry:  Speaker fees – Bright Eye Hospital group, China 2022  Royalties – Textbooks: 1, Clinical Orthoptics; 2, Visual fields via the visual pathway  Grant funding - 1, Norway Research Foundation project funding for stroke/vision network. 2019-2023; 2, Fight for Sight project funding for vision rehabilitation of hemianopia. 2020-2023; 3, NIHR Research for Patient Benefit project funding for vision rehabilitation of hemianopia. 2019-2023; 4, Research England / University of Liverpool HLS Policy Support project funding for clinical effectiveness of orthoptic assessment for stroke survivors.2022-2023.  Consultancy – Haag Streit AG, 2019 |
| Maria Begona Coco Martin | PhD, Department of Medicine, Dermatology and Toxicology, Neurology Teaching Unit, University of Valladolid, Spain  Spanish cooperative research network on stroke (RICORS-ICTUS) | Intellectual:  President of the European Society for Low Vision Research and Rehabilitation (ESLRR)  EUNOS Committee Member Visual Rehabilitation and Psychophysics |
| Celine Gillebert | Discipline: Neuropsychology  Affiliations:   1. Department Brain and Cognition, Leuven Brain Institute (LBI), KU Leuven, Leuven, Belgium. 2. TRACE, Centre for Translational Psychological Research, KU Leuven - Hospital East-Limbourgh, Genk, Belgium. | None |
| Lauren Hepworth | Discipline: Orthoptics  Affiliation: Institute of Population Health, University of Liverpool | Intellectual COI:  Guideline and stroke/vision committees:  1, Member of BIOS stroke and neuro-rehab Clinical Advisory Group.  2, Member of UK National Stroke Guideline vision topic group  Involved in team that developed VISA and VFAST |
| Luis Leal Vega | PhD, Department of Medicine, Dermatology and Toxicology, Neurology Teaching Unit, University of Valladolid, Spain  Spanish cooperative research network on stroke (RICORS-ICTUS) | None |
| Anja Palmowski-Wolfe | University Eye Hospital Basel | No financial disclosures relevant to this manuscript.  Intellectual: EUNOS Committee Member Visual Rehabilitation and Psychophysics |
| Eleni Papageorgiou | Department of Ophthalmology, University Hospital of Larissa, Greece | Nothing to declare |
| Stephen James Ryan | Neurologist, Dept. of Neurology, Oslo University Hospital | None |
| Karolina Skorkovska | 1. Department of Optometry and Orthoptics, Masaryk University, Brno, Czech Republic 2. Department of Ophthalmology and Optometry, St. Ann University Hospital, Brno, Czech Republic | none |
| Anne Hege Aamodt | Neurologist, Dept. of Neurology, Oslo University Hospital, Oslo, Norway and the Norwegian University of Science and Technology, Department of Neuromedicine and Movement Science, Trondheim, Norway | No financial disclosures relevant to this manuscript.  Relationships with industry:  Speaker fees – Teva, Abbvie, Novartis, Roche, Lundbeck.  Advisory Board – Lundbeck, Abbvie, Pfizer.  Research grant – unrestricted research grant from Boehringer Ingelheim to study medication in the TenCRAOS trial. |

**Supplementary table 2; List of abbreviations**

ADL Activities of Daily Living

ANOVA Analysis of Variance

ANCOVA Analysis of Covariance

BEFAST Balance, Eyes, Face, Arm, Speech, Time

BEN Batterie d’Évaluation de la Négligence spatiale unilatérale

BI Barthel Index

BIT Behaviour Inattention Test

BIT-C Behaviour Inattention Test-conventional section

BMT Bimanual Mirror Therapy

BVD Binocular Vision Dysfunction

CBS Catherine Bergego Scale

CI Confidence Interval

CMT Congruent Movement Training

CPSS Cincinnati Prehospital Stroke Scale

CRAO Central Retinal Artery Occlusion

cTBS Continuous theta burst stimulation

CT Computed Tomography

CVI Certificate of Visual Impairment

CVSQ Cerebral Vision Screening Questionnaire

EDTRS LogMAR visual acuity chart

EHC Eye Hand Coordination

ERBI Early Rehabilitation Barthel Index

ESO European Stroke Organisation

FAM Functional Assessment Measure

FAST Face, Arm, Speech, Time

FES Functional Electrical Stimulation

FIM Functional Independence Measure

FMA-UE Fugl Meyer Assessment for Upper Extremity

FVE Free Visual Exploration

GOSE Glasgow Functional Outcome Scale - Extended

GRADE Grading of Recommendations, Assessment, Development and Evaluation

HEP Hemifield Eye Patching

HINTS Head, Impulse, Nystagmus, Test of Skew

HVF Humphrey Visual Field

INO Internuclear Ophthalmoplegia

IQR Interquartile Range

IVIS Impact of Visual Impairment after Stroke

IVT Intravenous Thrombolysis

KAT Kinethetic Ability Trainer

K-MBI Korean version Modified Barthel Index

KF-NAP Kessler Foundation Neglect Assessment Process

LBT Line Bisection Test

LOTCA Lowenstein Occupational Therapy Cognitive Assessment

MAC Mobility Assessment Course

MBI Modified Barthel Index

MMSE Mini Mental State Examination

MP Methylphenidate

MRFn Melbourne Rapid Field-neural

mRS modified Rankin Scale

MS Multisensory Stimulation

MT Mirror Therapy

MVF Mirror Visual Feedback

MVPT Motor-free Visual Perception Test

MVPT-3 Motor-free Visual Perception Test 3^rd^ edition

MVPT-V Motor-free Visual Perception Test – Vertical version

MWG Module Working Group

NA-CRAO Non Arteritic Central Retinal Artery Occlusion

NEADL Nottingham Extended Activities of Daily Living scale

NEC Neuro Eye Coach

NEI VFQ National Eye Institute Visual Function Questionnaire

NEI VFQ National Eye Institute Visual Function Questionnaire

NHS National Health Service

NIBS Non Invasive Brain Stimulation

NIHSS National Institute of Health Stroke Scale

NVT Neuro Vision Technology

OCS Oxford Cognitive Screen

OKS Optokinetic Stimulation

PA Prism Adaptation

PARO Interactive robotic toy

PAT Prism Adaptation Therapy

PARO Interactive robotic toy

PICO Population, Intervention, Comparator, Outcome

PMD Perimetric Mean Deviation

PreHAST Prehospital Ambulance Stroke Test

RAT Robot-assisted Arm Training

READ cueing assisted reading therapy

RCT Randomised Controlled Trial

rtACS Repetitive Transorbital Alternating Current Stimulation

rTMS Repetitive Transcranial Magnetic Stimulation

rtPA Recombinant Tissue-type Plasminogen Activator

RUNS Rapid Unilateral Neglect Screening

QoL Quality of Life

QUADAS-2 Quality Assessment of Diagnostic Accuracy Studies-2

SAT Stimulus-driven Attention Test

SCT Star Cancellation Test

SD Standard Deviation

SE Standard Error

SEM Standard Error of the Mean

SINGER Scores of Independence for Neurologic and Geriatric Rehabilitation

SOP Standardised Operating Procedure

SVDST Stroke Vision Defects Screening Tool

tDCS Transcranial Direct Current Stimulation

TOJ Temporal Order Judgement

UMT Unimanual Mirror Therapy

USN Unilateral Spatial Neglect

VA Visual Acuity

V-FAST Vision, Face, Arm, Speech, Time

VISA Vision Impairment Screening Assessment

VLBM Voxel-based Behaviour Mapping

VR Virtual Reality

VSE Visual Scanning Exercises

VSN Visuospatial Neglect

VSS Visuospatial Scale

VST Visual Scanning Therapy

WHODAS World Health Organisation Disability Assessment Scale

**Supplementary table 3; Ranking of outcomes for PICO questions**

| **PICO** | **Outcome** | **Mean score** |
| --- | --- | --- |
| **PICO 1** | Feasibility  Sensitivity  Specificity  Acceptability  Detection rate  Timing of screening  Timing of screening  Reports of visual symptoms  Negative predictive value  Detection percentages  Positive predictive value  Detection numbers | 8.0  7.7  7.5  7.3  7.1  6.9  6.8  6.3  6.2  6.1  5.9  5.2 |
| **PICO 2** | Timing of assessment/screening  Core outcome measures  Units of measurement - quality of life  Units of measurement - functional score  Units of measurement - activity of daily living  Core outcome set  Units of measurement - falls  Reports of visual symptoms  Length of hospital stay | 7.8  7.7  7.6  7.4  7.3  7.2  6.3  6.1  5.5 |
| **PICO 3** | Sensitivity  Specificity  Feasibility  Units of measurement - quality of life  Detection rate  Units of measurement - activity of daily living  Acceptability  Units of measurement - functional score  Units of measurement - visual field area  Negative predictive value  Units of measurement - visual field sensitivity  Positive predictive value  Units of measurement - falls  Time to diagnosis  Time to rehabilitation  Reports of visual symptoms  Length of hospital stay | 8.0  8.0  7.7  7.5  7.2  7.2  7.1  7.1  7.0  6.9  6.8  6.8  6.7  6.6  6.3  6.3  5.1 |
| **PICO 4** | Sensitivity  Specificity  Units of measurement - visual acuity  Feasibility  Units of measurement - quality of life  Detection rate  Acceptability  Positive predictive value  Negative predictive value  Reports of visual symptoms  Units of measurement - functional score  Units of measurement - activity of daily living  Time to diagnosis  Units of measurement - falls  Units of measurement - contrast sensitivity  Units of measurement - colour  Time to rehabilitation  Length of hospital stay | 8.0  7.8  7.4  7.3  7.3  7.1  7.0  6.9  6.9  6.8  6.8  6.6  6.4  6.2  6.1  5.9  5.8  5.1 |
| **PICO 5** | Sensitivity  Specificity  Units of measurement - quality of life  Feasibility  Units of measurement - activity of daily living  Units of measurement - field of binocular single vision  Detection rate  Units of measurement - angle of deviation  Units of measurement - functional score  Acceptability  Units of measurement - Ocular rotation  Positive predictive value  Reports of visual symptoms  Negative predictive value  Time to diagnosis  Units of measurement - falls  Time to rehabilitation  Length of hospital stay | 7.9  7.9  7.2  7.1  7.1  7.0  6.9  6.8  6.8  6.8  6.5  6.4  6.4  6.3  6.3  6.3  6.2  5.2 |
| **PICO 6** | Sensitivity  Specificity  Units of measurement - quality of life  Feasibility  Detection rate  Units of measurement - activity of daily living  Units of measurement - functional score  Acceptability  Positive predictive value  Negative predictive value  Time to diagnosis  Reports of visual symptoms  Time to rehabilitation  Units of measurement - falls  Length of hospital stay | 8.0  7.8  7.4  7.3  7.2  7.2  7.0  6.9  6.8  6.7  6.5  6.4  6.2  5.9  5.0 |
| **PICO 7** | Sensitivity  Specificity  Units of measurement - neglect battery  Units of measurement - quality of life  Feasibility  Detection rate  Units of measurement - activity of daily living  Units of measurement - functional score  Units of measurement - cancellation task  Positive predictive value  Negative predictive value  Acceptability  Units of measurement - clock drawing  Units of measurement - room description  Time to diagnosis  Units of measurement - line bisection  Units of measurement -behavioural task  Time to rehabilitation  Reports of visual symptoms  Units of measurement - falls  Length of hospital stay | 8.1  7.9  7.7  7.3  7.2  7.2  7.2  7.0  7.0  6.7  6.7  6.7  6.6  6.6  6.5  6.5  6.5  6.3  6.3  6.3  5.1 |
| **PICO 8** | Change in visual field boundaries/area  Change in scanning detection speed  Change in scanning detection accuracy  Change in quality of life  Change in activities of daily living  Accident rate  Fall rate  Driving ability  Change in reading speed  Change in reading accuracy  Change in symptom reporting | 7.5  7.7  7.6  7.4  7.1  6.9  6.9  6.9  6.8  6.7  6.2 |
| **PICO 9** | Change in visual field boundaries/area  Change in quality of life  Change in activities of daily living  Change in scanning detection speed  Change in scanning detection accuracy  Driving ability  Change in symptom reporting  Accident rate  Fall rate | 7.6  7.3  7.2  7.0  6.9  6.8  6.6  6.6  6.3 |
| **PICO 10** | Change in visual acuity  Change in activities of daily living  Change in quality of life  Change in reading speed  Driving ability  Change in reading accuracy  accident rate  Fall rate  Change in common symptoms | 7.4  7.1  7.1  6.9  6.8  6.7  6.4  6.3  6.2 |
| **PICO 11** | Change in primary deviation angle  Change in quality of life  Change in binocular field of single vision  Change in activities of daily living  Saccadic parameters  Fixation stability  Driving ability  Change in ocular rotation grade  Fix and follow response  Accident rate  Change in symptom reporting  Fall rate  Nystagmus waveform | 7.3  7.2  7.1  7.1  6.9  6.8  6.8  6.7  6.7  6.6  6.4  6.3  6.1 |
| **PICO 12** | Change in neglect test score  Change in object detection accuracy  Awareness of neglect  Change in activities of daily living  Change in quality of life  Change in object detection speed  Attention  Driving ability  Accident rate  Fall rate  Alertness  Discharge destination | 7.5  7.3  7.3  7.2  7.2  7.1  6.8  6.7  6.4  6.4  6.2  5.7 |
| **PICO 13** | Change in quality of life  Change in activities of daily living  Change in visual symptom reporting  Accident rate  Fall rate | 7.3  7.0  6.6  6.3  6.3 |

**Supplementary Table 4: Search terms**

**Databases:**

- MEDLINE (to March 2023);
- Scopus (to March 2023);
- CINAHL (to March 2023);
- AMED (to March 2023).

**PICO 1**

| Cerebrovascular disorders/  Brain ischaemia/  Intracranial Arterial Disease  Intracranial Arteriovenous Malformations/  Intracranial Embolism and Thrombosis/  Stroke/ | Eye Movements/  Eye/  Eye Disease/  Visually Impaired Persons/  Vision Disorders/  Blindness/  Diplopia/  Vision, Binocular/  Vision, Monocular/  Visual Acuity/  Visual Fields/  Vision, Low/  Ocular Motility Disorders/  Blindness, Cortical/  Hemianopsia/  Abducens Nerve Diseases/  Abducens Nerve/  Oculomotor Nerve/  Trochlear Nerve/  Visual Perception/  Nystagmus  Strabismus  Smooth pursuits  SaccadesDepth perception  Stereopsis  Gaze disorder  Internuclear ophthalmoplegia  Parinaud’s syndrome  Weber’s syndrome  Skew deviation  Conjugate deviation  Oscillopsia  Visual tracking  Agnosia  Hallucinations |  |
| --- | --- | --- |
| Stroke/  Poststroke/  post-stroke/  Cerebral hemorrhage/  Intracranial hemorrhage/  intracerebral hemorrhage  Cerebral Infarction/  Cerebrovascular accident/  Cerebral vascular accident/  CVA/ | Neglect/  Hemineglect/  Visual perception/  Visuoperceptual/  Attentional bias/  Attentional orientation/  Spatial processing/  Attention/  hemi-neglect  perceptual-attention  hemi-attention  inattention  hemi-inattention  hemispatial  hemi-spatial  visuospatial  spatial bias  spatial impairment  spatial awareness  spatial unawareness  biased exploration  awareness bias  directional bias  perceptual bias  bisection bias | Neuropsychological tests/  test  tests  tested  testing  battery  batteries  evaluate  evaluated  evaluation  screen  task  measure  assessment  assessed psychometric  cognitive  examination |
| OR | OR | OR |
| AND | | |

**PICOs 2-7**

| Cerebrovascular disorders/  Brain ischaemia/  Intracranial Arterial  Disease  Intracranial Arteriovenous Malformations/  Intracranial Embolism and Thrombosis/  Stroke/ | Eye Movements/  Eye/  Eye Disease/  Visually Impaired Persons/  Vision Disorders/  Blindness/  Diplopia/  Vision, Binocular/  Vision, Monocular/  Visual Acuity/  Visual Fields/  Vision, Low/  Ocular Motility Disorders/  Blindness, Cortical/  Hemianopsia/  Abducens Nerve Diseases/  Abducens Nerve/  Oculomotor Nerve/  Trochlear Nerve/  Visual Perception/  Nystagmus  strabismus  smooth pursuits  saccades  depth perception  stereopsis  gaze disorder  internuclear ophthalmoplegia  Parinaud’s syndrome  Weber’s syndrome  skew deviation  conjugate deviation  oscillopsia  visual tracking  agnosia  hallucinations |  |
| --- | --- | --- |
| Stroke/  Poststroke/  post-stroke/  Cerebral hemorrhage/  Intracranial hemorrhage/  intracerebral hemorrhage  Cerebral Infarction/  Cerebrovascular accident/  Cerebral vascular accident/  CVA/ | Neglect/  Hemineglect/  Visual perception/  Visuoperceptual/  Attentional bias/  Attentional orientation/  Spatial processing/  Attention/  hemi-neglect  perceptual-attention  hemi-attention  inattention  hemi-inattention  hemispatial  hemi-spatial  visuospatial  spatial bias  spatial impairment  spatial awareness  spatial unawareness  biased exploration  awareness bias  directional bias  perceptual bias  bisection bias | Neuropsychological tests/  test  tests  tested  testing  battery  batteries  evaluate  evaluated  evaluation  screen  task  measure  assessment  assessed psychometric  cognitive  examination |
| OR | OR | OR |
| AND | | |

**PICO 8**

| Cerebrovascular disorders/  Brain ischaemia/  Intracranial Arterial Disease  Intracranial Arteriovenous Malformations/  Intracranial Embolism and Thrombosis/  Stroke/ | Eye Movements/  Eye/  Eye Disease/  Visually Impaired Persons/  Vision Disorders/  Blindness/  Diplopia/  Vision, Binocular/  Vision, Monocular/  Visual Acuity/  Visual Fields/  Vision, Low/  Ocular Motility Disorders/  Blindness, Cortical/  Hemianopsia/  Abducens Nerve Diseases/  Abducens Nerve/  Oculomotor Nerve/  Trochlear Nerve/  Visual Perception/  Nystagmus  strabismus  smooth pursuits  saccades  depth perception  stereopsis  gaze disorder  internuclear ophthalmoplegia  Parinaud’s syndrome  Weber’s syndrome  skew deviation  conjugate deviation  oscillopsia  visual tracking  agnosia  hallucinations |  |
| --- | --- | --- |
| Cerebrovascular disorders/  exp Basal ganglia cerebrovascular disease/  exp Brain ischemia/  exp Carotid artery diseases/  Cerebral small vessel diseases/  Stroke, lacunar/  exp Intracranial arterial diseases/  exp Intracranial arteriovenous malformations/  exp Intracranial embolism and thrombosis/  exp Intracranial hemorrhages/  Stroke/  exp Brain infarction/  Vasospasm, intracranial/  Vertebral artery dissection/ stroke  poststroke  post‐stroke  cerebrovasc$  brain vasc$  cerebral vasc$  cva$  apoplex$  SAH. brain$  cerebr$  cerebell$  intracran$  intracerebral  ischemi$  infarct$  thrombo$  emboli$  occlus$ intracranial  subarachnoid  haemorrhage$  hemorrhage$  haematoma$  hematoma$  bleed$  hemiplegia  exp Paresis/ hemipleg$  hemipar$  paresis  paretic | exp Eye/ exp Visually impaired persons/ exp Ocular physiological processes/  exp Diagnostic techniques, ophthalmological/ Optometry/  Orthoptics/ Eye diseases/  Vision disorders/  Eye manifestations/ Blindness/  Diplopia/ Vision, binocular/  Vision, monocular/  exp Visual acuity/  Visual fields/  Vision, low/  Perimetry/  Ophthalmology/  Vision screening/ exp Ocular motility disorders/ exp Orbital diseases/  exp Pupil disorders/  exp Blindness, cortical/  exp Hemianopsia/  Scotoma/ Abducens nerve/  Oculomotor nerve/  Trochlear nerve/ nystagmus  smooth pursuit  saccades  depth perception  stereopsis  gaze disorder$  ophthalmol$  optic nerve intranuclear ophthalmoplegia parinaud's syndrome  weber's syndrome  skew deviation  conjugate deviation  one and a half syndrome visual$  vision  eyesight  sight  problem$  disorder$  impair$  disabilit$  loss  disease$  defect$  manifestation$  screening  test$  examination$ hemianop$  blindness  low vision  scotoma  diplopia  optometr$  ocular  orthoptic$ oscillopsia  visual tracking  fresnel prism$. III nerve palsy  IV nerve palsy  VI nerve palsy  third nerve palsy  fourth nerve palsy  sixth nerve palsy | Randomized Controlled Trials as Topic/ Random allocation/ Controlled Clinical Trials as Topic/ Control groups/ Clinical trials as topic/  Clinical trials, phase I as topic/  Clinical trials, phase II as topic/  Clinical trials, phase III as topic/  Clinical trials, phase IV as topic/ Double‐blind method/ Single‐blind method/ Placebos/ Placebo effect/ Cross‐over studies/ randomized controlled trial controlled clinical trial clinical trial or clinical trial phase I clinical trial phase II clinical trial phase III clinical trial phase IV random$  RCT  RCTs controlled  trial$  stud$ clinical$  trial$ control  treatment experiment$ intervention  group$  subject$  patient$  quasi‐random$ pseudo‐random$  xperiment$ conservative  therapy  procedure or manage$ singl$ blind$  doubl$ blind$  mask$ cross‐over  cross over crossover  placebo$  sham assign$  allocat$ |
| OR | OR | OR |
| AND | | |

**PICO 9**

| Cerebrovascular disorders/  Brain ischaemia/  Intracranial Arterial Disease  Intracranial Arteriovenous Malformations/  Intracranial Embolism and Thrombosis/  Stroke/ | Eye Movements/  Eye/  Eye Disease/  Visually Impaired Persons/  Vision Disorders/  Blindness/  Diplopia/  Vision, Binocular/  Vision, Monocular/  Visual Acuity/  Visual Fields/  Vision, Low/  Ocular Motility Disorders/  Blindness, Cortical/  Hemianopsia/  Abducens Nerve Diseases/  Abducens Nerve/  Oculomotor Nerve/  Trochlear Nerve/  Visual Perception/  Nystagmus  strabismus  smooth pursuits  saccades  depth perception  stereopsis  gaze disorder  internuclear ophthalmoplegia  Parinaud’s syndrome  Weber’s syndrome  skew deviation  conjugate deviation  oscillopsia  visual tracking  agnosia  hallucinations |
| --- | --- |
| Randomized Controlled Trial/ Controlled Clinical Trial/ randomized  randomised placebo  drug therapy randomly trial  groups | exp Retinal artery/ exp Retinal artery occlusion/ retina* arter* occlus*  obstruct*  clos*  stricture*  steno*  block*  embolism* CRAO |
| OR | OR |
| AND | |

**PICO 10**

| Cerebrovascular disorders/  Brain ischaemia/  Intracranial Arterial Disease  Intracranial Arteriovenous Malformations/  Intracranial Embolism and Thrombosis/  Stroke/ | Eye Movements/  Eye/  Eye Disease/  Visually Impaired Persons/  Vision Disorders/  Blindness/  Diplopia/  Vision, Binocular/  Vision, Monocular/  Visual Acuity/  Visual Fields/  Vision, Low/  Ocular Motility Disorders/  Blindness, Cortical/  Hemianopsia/  Abducens Nerve Diseases/  Abducens Nerve/  Oculomotor Nerve/  Trochlear Nerve/  Visual Perception/  Nystagmus  strabismus  smooth pursuits  saccades  depth perception  stereopsis  gaze disorder  internuclear ophthalmoplegia  Parinaud’s syndrome  Weber’s syndrome  skew deviation  conjugate deviation  oscillopsia  visual tracking  agnosia  hallucinations |  |
| --- | --- | --- |
| Cerebrovascular disorders/  exp Basal ganglia cerebrovascular disease/  exp Brain ischemia/  exp Carotid artery diseases/  Cerebral small vessel diseases/  Stroke, lacunar/  exp Intracranial arterial diseases/  exp Intracranial arteriovenous malformations/  exp Intracranial embolism and thrombosis/  exp Intracranial hemorrhages/  Stroke/  exp Brain infarction/  Vasospasm, intracranial/  Vertebral artery dissection/ stroke  poststroke  post‐stroke  cerebrovasc$  brain vasc$  cerebral vasc$  cva$  apoplex$  SAH. brain$  cerebr$  cerebell$  intracran$  intracerebral  isch?emi$  infarct$  thrombo$  emboli$  occlus$ intracranial  subarachnoid  haemorrhage$  hemorrhage$  haematoma$  hematoma$  bleed$  hemiplegia  exp Paresis/ hemipleg$  hemipar$  paresis  paretic | exp Eye/ exp Visually impaired persons/ exp Ocular physiological processes/  exp Diagnostic techniques, ophthalmological/ Optometry/  Orthoptics/ Eye diseases/  Vision disorders/  Eye manifestations/ Blindness/  Diplopia/ Vision, binocular/  Vision, monocular/  exp Visual acuity/  Visual fields/  Vision, low/  Perimetry/  Ophthalmology/  Vision screening/  exp Eye diseases, hereditary/  exp Eye hemorrhage/  exp Lacrimal apparatus diseases/  exp Lens diseases/  exp Ocular hypertension/  exp Ocular hypotension/  exp Ocular motility disorders/  exp Optic nerve diseases/ exp Orbital diseases/  exp Pupil disorders/  exp Refractive errors/  exp Retinal diseases/  exp Blindness, cortical/  exp Hemianopsia/  exp Vitreoretinopathy, proliferative/  exp Vitreous detachment/ Scotoma/  Abducens nerve/  Oculomotor nerve/  Trochlear nerve/  Nystagmus  smooth pursuit  saccades  depth perception  stereopsis  gaze disorder$  retinal  retinopathy  macular degeneration  glaucoma  cataract$  ophthalmol$  optic nerve  intranuclear ophthalmoplegia parinaud's syndrome  weber's syndrome  skew deviation  conjugate deviation  one and a half syndrome  visual$  vision  eyesight  sight  problem$  disorder$  impair$  disabilit$  loss  disease$  defect$  manifestation$  screening  test$  examination$ hemianop$  blindness  low vision  refractive errors vitreoretinopathy  vitreous detachment  scotoma  diplopia  optometr$  ocular  orthoptic$ oscillopsia  visual tracking  fresnel prism$. III nerve palsy  IV nerve palsy  VI nerve palsy  third nerve palsy  fourth nerve palsy  sixth nerve palsy | Randomized Controlled Trials as Topic/ Random allocation/ Controlled Clinical Trials as Topic/ Control groups/ Clinical trials as topic/  Clinical trials, phase I as topic/  Clinical trials, phase II as topic/  Clinical trials, phase III as topic/  Clinical trials, phase IV as topic/ Double‐blind method/ Single‐blind method/ Placebos/ Placebo effect/ Cross‐over studies/ randomized controlled trial controlled clinical trial clinical trial or clinical trial phase I clinical trial phase II clinical trial phase III clinical trial phase IV random$  RCT  RCTs controlled  trial$  stud$ clinical$  trial$ control  treatment experiment$ intervention  group$  subject$  patient$  quasi‐random$ pseudo‐random$  xperiment$ conservative  therapy  procedure or manage$ singl$ blind$  doubl$ blind$  mask$ cross‐over  cross over crossover  placebo$  sham assign$  allocat$ |
| OR | OR | OR |
| AND | | |

**PICO 11**

| Cerebrovascular disorders/  Brain ischaemia/  Intracranial Arterial Disease  Intracranial Arteriovenous Malformations/  Intracranial Embolism and Thrombosis/  Stroke/ | Eye Movements/  Eye/  Eye Disease/  Visually Impaired Persons/  Vision Disorders/  Blindness/  Diplopia/  Vision, Binocular/  Vision, Monocular/  Visual Acuity/  Visual Fields/  Vision, Low/  Ocular Motility Disorders/  Blindness, Cortical/  Hemianopsia/  Abducens Nerve Diseases/  Abducens Nerve/  Oculomotor Nerve/  Trochlear Nerve/  Visual Perception/  Nystagmus  strabismus  smooth pursuits  saccades  depth perception  stereopsis  gaze disorder  internuclear ophthalmoplegia  Parinaud’s syndrome  Weber’s syndrome  skew deviation  conjugate deviation  oscillopsia  visual tracking  agnosia  hallucinations |  |
| --- | --- | --- |
| Cerebrovascular disorders/  exp Basal ganglia cerebrovascular disease/  exp Brain ischemia/  exp Carotid artery diseases/  Cerebral small vessel diseases/  Stroke, lacunar/  exp Intracranial arterial diseases/  exp Intracranial arteriovenous malformations/  exp Intracranial embolism and thrombosis/  exp Intracranial hemorrhages/  Stroke/  exp Brain infarction/  Vasospasm, intracranial/  Vertebral artery dissection/ stroke  poststroke  post‐stroke  cerebrovasc$  brain vasc$  cerebral vasc$  cva$  apoplex$  SAH. brain$  cerebr$  cerebell$  intracran$  intracerebral  isch?emi$  infarct$  thrombo$  emboli$  occlus$ intracranial  subarachnoid  haemorrhage$  hemorrhage$  haematoma$  hematoma$  bleed$  hemiplegia  exp Paresis/ hemipleg$  hemipar$  paresis  paretic | exp Eye/ exp Visually impaired persons/ exp Ocular physiological processes/  exp Diagnostic techniques, ophthalmological/ Optometry/  Orthoptics/ Eye diseases/  Vision disorders/  Eye manifestations/ Blindness/  Diplopia/ Vision, binocular/  Vision, monocular/  exp Visual acuity/  Visual fields/  Vision, low/  Perimetry/  Ophthalmology/  Vision screening/  exp Eye diseases, hereditary/  exp Eye hemorrhage/  exp Lacrimal apparatus diseases/  exp Lens diseases/  exp Ocular hypertension/  exp Ocular hypotension/  exp Ocular motility disorders/  exp Optic nerve diseases/ exp Orbital diseases/  exp Pupil disorders/  exp Refractive errors/  exp Retinal diseases/  exp Blindness, cortical/  exp Hemianopsia/  exp Vitreoretinopathy, proliferative/  exp Vitreous detachment/ Scotoma/  Abducens nerve/  Oculomotor nerve/  Trochlear nerve/  Nystagmus  smooth pursuit  saccades  depth perception  stereopsis  gaze disorder$  retinal  retinopathy  macular degeneration  glaucoma  cataract$  ophthalmol$  optic nerve  intranuclear ophthalmoplegia  parinaud's syndrome  weber's syndrome  skew deviation  conjugate deviation  one and a half syndrome  visual$  vision  eyesight  sight  problem$  disorder$  impair$  disabilit$  loss  disease$  defect$  manifestation$  screening  test$  examination$ hemianop$  blindness  low vision  refractive errors vitreoretinopathy  vitreous detachment  scotoma  diplopia  optometr$  ocular  orthoptic$ oscillopsia  visual tracking  fresnel prism$. III nerve palsy  IV nerve palsy  VI nerve palsy  third nerve palsy  fourth nerve palsy  sixth nerve palsy | Randomized Controlled Trials as Topic/ Random allocation/ Controlled Clinical Trials as Topic/ Control groups/ Clinical trials as topic/  Clinical trials, phase I as topic/  Clinical trials, phase II as topic/  Clinical trials, phase III as topic/  Clinical trials, phase IV as topic/ Double‐blind method/ Single‐blind method/ Placebos/ Placebo effect/ Cross‐over studies/ randomized controlled trial controlled clinical trial clinical trial or clinical trial phase I clinical trial phase II clinical trial phase III clinical trial phase IV random$  RCT  RCTs controlled  trial$  stud$ clinical$  trial$ control  treatment experiment$ intervention  group$  subject$  patient$  quasi‐random$ pseudo‐random$  xperiment$ conservative  therapy  procedure or manage$ singl$ blind$  doubl$ blind$  mask$ cross‐over  cross over crossover  placebo$  sham assign$  allocat$ |
| Randomized controlled trial/ randomized  randomised placebo randomly  trial  groups  . | exp Brain injuries/ brain injur$ ABI  TBI  non TBI | exp Eye/ exp Visually Impaired Persons/ exp Ocular Physiological Processes/ exp Diagnostic Techniques, Ophthalmological/ exp Optometry/ exp Orthoptics/ exp Eye Diseases/ exp Vision Disorders/ exp Eye Manifestations/ exp Blindness/ exp Diplopia/ Vision, Binocular/ Vision, Monocular/ exp Visual Acuity/ Visual Fields/ Vision, Low/ exp Visual Field Tests/ Ophthalmology/ Vision Screening/ Eye Diseases, Hereditary/ exp Ocular Motility Disorders/ exp Optic Nerve Diseases/ Enophthalmos/ exp Pupil Disorders/ exp Refractive Errors/ Blindness, Cortical/ exp Hemianopsia/ Scotoma/  Abducens Nerve/ Oculomotor Nerve/ Trochlear Nerve/  smooth pursuit  saccades  saccadic  depth perception  stereopsis  gaze disorder$  ophthalm$  optic nerve$ ocular muscle$  ocular align$ esotropi$  exotropi$  hypertropi$  hypotropi$  cyclotropi$  intranuclear ophthalmoplegia  parinaud's syndrome  weber's syndrome  skew deviation  conjugate deviation visual$  vision  eye  eyes  eyesight  sight  problem$  disorder$  impair$  disabilit$  loss  disease$  defect$  manifestation$  screening  test$ or examination$  reading difficult$  reading impair$  hemianop$  blindness  low vision  refractive errors  scotoma  diplopia  optometr$  ocular  orthoptic$ oscillopsia  visual tracking  fresnel prism$ downbeat nystagmus  upbeat nystagmus  vertical nystagmus).tw. gaze$ deficit$  gaze$ pals$  gaze$ disorder$ III nerve pals$  IV nerve pals$  VI nerve pals$  third nerve pals$  fourth nerve pals$  sixth nerve pals$ |
| OR | OR | OR |
| AND | | |

**PICO 12**

| Cerebrovascular disorders/  Brain ischaemia/  Intracranial Arterial Disease  Intracranial Arteriovenous Malformations/  Intracranial Embolism and Thrombosis/  Stroke/ | Eye Movements/  Eye/  Eye Disease/  Visually Impaired Persons/  Vision Disorders/  Blindness/  Diplopia/  Vision, Binocular/  Vision, Monocular/  Visual Acuity/  Visual Fields/  Vision, Low/  Ocular Motility Disorders/  Blindness, Cortical/  Hemianopsia/  Abducens Nerve Diseases/  Abducens Nerve/  Oculomotor Nerve/  Trochlear Nerve/  Visual Perception/  Nystagmus  strabismus  smooth pursuits  saccades  depth perception  stereopsis  gaze disorder  internuclear ophthalmoplegia  Parinaud’s syndrome  Weber’s syndrome  skew deviation  conjugate deviation  oscillopsia  visual tracking  agnosia  hallucinations |  |  |  |
| --- | --- | --- | --- | --- |
| Cerebrovascular accident/  cerebral vascular accident  CVA Stroke/ | Neglect  Hemineglect  Hemi-inattention  Perceptual disorders/ | evaluation  assessment  tool  treatment  intervention strategy  therapeutics | virtual reality  virtual environment  computer-based  virtual  user-computer  interface  computer simulation  therapy computer assisted |  |
| Cerebrovascular disorders/  Basal ganglia cerebrovascular disease/  Brain ischemia  exp Carotid artery diseases/  exp Cerebral small vessel diseases/  exp Intracranial arterial diseases/  exp Intracranial embolism and thrombosis/  exp Intracranial hemorrhages/  Stroke/  exp Brain infarction/  Stroke, lacunar/  Vasospasm, intracranial/  Vertebral artery dissection/  Carotid stenosis/  exp Carotid artery injuries/  Intracranial arterial diseases/  Cerebral arterial diseases/  Infarction, anterior cerebral artery/ Infarction, middle cerebral artery/  Infarction, posterior cerebral artery/  exp Carotid arteries/ Endarterectomy, carotid/  stroke$  poststroke  apoplex$  cerebral vasc$  brain vasc$  cerebrovasc$  cva$  SAH  brain$  cerebr$  cerebell$  vertebrobasil$  hemispher$  intracran$  intracerebral  infratentorial  supratentorial  middle cerebral arter$  MCA$  anterior circulation  posterior circulation  basilar arter$  vertebral arter$  space‐occupying  isch?emi$  infarct$  thrombo$  emboli$  occlus$  hypoxi$ brain$  cerebr$  cerebell$  intracerebral  intracran$  parenchymal  intraparenchymal  intraventricular  infratentorial  supratentorial  basal gangli$  putaminal  putamen  posterior fossa  hemispher$  subarachnoid  hemorrhag$  hematoma$  bleed$ | exp Perceptual disorders/  exp Perception/ Vision disorders/  Alice in wonderland syndrome/  Amblyopia/  Blindness/  Blindness, cortical/  Color vision defects/ Diplopia/  Hemianopsia/  Photophobia/  Scotoma/  Vision, low/ percept$ impair$  percept$ problem$ percept$ abilit$ percept$ deficit$ percept$ distortion$ percept$ defect$ percept$ disabilit$ percept$ disturbance$ percept$ disorder$ percept$ discriminat$  agnosis  agnosia  prosopagnosia  prosophthalmia  Todd syndrome  allesthesia$  synesthesia$  hypoesthesia  hyperesthesia Vision, ocular/  exp Mesopic vision/ Night vision/  amblyop? aniseikonia oscillopsia xanthopsia diplop$  polyop$ metamorphopsia micropsia  vision  visual  visualpercept$ visuospatial visuoconstruct$ ocular  optokinetic  optic$  oculomotor spatial) illusion  blurry  overload  double  percept$  perceive$  discriminat$ distinguish$  recept$  sensitiv$  hallucination$ abnormalit$ distortion$ disturbance$  anomal$ disorientation  allachethesia  deficit$  defect$  disabilit$  disorder$  processing dysfunction$  recogn$  interpretation  analysis  comprehension stereoillusion  kakopsia  kalopsia  pelopsia  achromatopsia akinetopsia  telopsia  stereopsis  palinopsia  teleopsia simultanagnosia  entomopia  palinopsia  asteropsis  strabismus  Anton syndrome  Balint syndrome blindsight  Hyperchromatosis  fac$ intermetamorphosis visual anoneria figure recogn$.  shape recogn$.  orientation recogn$.  form recogn$.  colo?r recogn$.  textur$ recogn$.  crowding recogn$.  contour recogn$.  object recogn$.  face recogn$.  faces recogn$. | Randomized controlled trial/ controlled clinical trial. randomized placebo  clinical trials as topic random$ trial |  |  |
| Cerebrovascular disorders/  exp Basal ganglia cerebrovascular disease/  exp Brain ischemia/ exp Carotid artery diseases/  exp Cerebrovascular trauma/  exp Intracranial arterial diseases/ exp Intracranial arteriovenous malformations/  exp Intracranial embolism and thrombosis/  exp Intracranial hemorrhages/  Stroke/  exp Brain infarction/ Stroke, lacunar/ Vasospasm, intracranial/ Vertebral artery dissection/  exp Hypoxia, brain/ stroke$  poststroke  post‐stroke  apoplex$  cerebral vasc$ cerebrovasc$  cva  SAH brain$  cerebr$  cerebell$  intracran$  intracerebral (isch?emi$  infarct$  thrombo$  emboli$  occlus$ brain$  cerebr$  cerebell$ intracerebral intracranial subarachnoid  haemorrhage$ hemorrhage$ haematoma$ hematoma$ bleed$ exp Hemiplegia/  exp Paresis/  hemipleg$  hemipar$  paresis  paretic cerebral  brain  subarachnoid  haemorrhage  haemorrhage  haematoma hematoma  bleed  acquired brain injur$ exp Brain damage, chronic/  Brain injuries/ | exp Perceptual Disorders/ exp Perception/ Attention/ Extinction (psychology)/ hemineglect  hemi‐neglect  unilateral neglect  spatial neglect perception  inattention  hemi‐inattention  attention  extinction perceptual  visuospatial  visuoperceptual  attentional  disorder$  deficit$  impairment$  abilit$ scanning$  training  re‐training  rehabilitation  intervention  therapy | Randomized Controlled Trials as Topic/ Random Allocation/ Controlled Clinical Trials as Topic/ Control groups/ Clinical trials as topic/  Clinical trials, phase I as topic/ Clinical trials, phase II as topic/  Clinical trials, phase III as topic/ Clinical trials, phase IV as topic/ Double‐blind method/ Single‐blind method/ Placebos/ Placebo effect/ Cross‐over studies/  randomized controlled trial controlled clinical trial clinical trial  clinical trial phase I  clinical trial phase II clinical trial phase III  clinical trial phase IV random$  RCT  RCTs controlled trial$  controlled stud$ clinical$ trial$  treatment  experiment$  intervention  group$  subject$  patient$ quasi‐random$  quasi random$  pseudo‐random$ pseudo random$ control  experiment$  conservative  treatment  or therapy procedure manage$ singl$  doubl$  tripl$  trebl$  blind$  mask$  cross‐over  cross over crossover placebo$  sham  trial assign$  allocat$ controls |  |  |
| Cerebrovascular disorders/  exp Basal ganglia cerebrovascular disease/  exp Brain ischemia/  exp Carotid artery diseases/  exp Intracranial arterial diseases/  exp Intracranial embolism and thrombosis/  exp Intracranial hemorrhages/  Stroke/  exp Brain infarction/  Stroke, lacunar/  Vasospasm, intracranial/  Vertebral artery dissection/  stroke  poststroke  post‐stroke  cerebrovasc$  brain vasc$  cerebral vasc$  cva$  apoplex$  SAH brain$  cerebr$  cerebell$  intracran$  intracerebral  isch?emi$  infarct$  thrombo$  emboli$  occlus$ brain$  cerebr$  cerebell$  intracerebral  intracranial  subarachnoid  haemorrhage$  hemorrhage$  haematoma$  hematoma$  bleed$ Hemiplegia/  exp Paresis/ hemipleg$  hemipar$  paresis  paretic).tw. | Perceptual disorders/ Perception/  exp Visual perception/  Space perception/  Attention/  Functional laterality/ Extinction,  psychological/  hemineglect  hemi‐neglect unilateral neglect  spatial neglect hemispatial neglect visual neglect perception  inattention  hemi‐inattention  extinction perceptual  visuospatial  visuoperceptual  attention$  disorder$  deficit$ or impairment$  abilit$  problem$ | drug effects  drug therapy  pharmacology. Dopamine agents/  1‐methyl‐4‐phenyl‐1,2,3,6‐tetrahydropyridine/  Amantadine/  Amphetamine/  Benserazide/  Benzphetamine/  Carbidopa/  Dihydroxyphenylalanine/  Dopamine/  Fusaric acid/  Levodopa/  Memantine/ Methamphetamine/ Dopamine agonists/  2,3,4,5‐tetrahydro‐7,8‐dihydroxy‐1‐phenyl‐1h‐3‐benzazepine/  Apomorphine/ Bromocriptine/ Dihydroergocornine/ Dihydroergocryptine/  Dihydroergotamine/  Dihydroergotoxine/  Fenoldopam/  Lisuride/  Metergoline/  Pergolide/  Piribedil/  Quinpirole/ dopamine  dopaminergic  amantadine  amphetamine  benserazide  benzphetamine  carbidopa  dihydroxyphenylalanine  fusaric acid  levodopa  L‐dopa  memantine  methamphetamine  apomorphine  bromocriptine  dihydroergocornine  dihydroergocryptine  dihydroergotamine  dihydroergotoxine  fenoldopam  lisuride  metergoline  pergolide  piribedil  quinpirole  rotigotine Adrenergic alpha‐agonists/  Epinephrine/  Etilefrine/  Naphazoline/  Norepinephrine/  Octopamine/ Oxymetazoline/  Phenylpropanolamine/ Synephrine/ Adrenergic alpha‐1 receptor agonists/ Ergotamine/  Mephentermine/  Metaraminol/  Methoxamine/  Midodrine/  Phenylephrine/  Adrenergic alpha‐2 receptor agonists/  Clonidine/  Dexmedetomidine/ Guanabenz/ Guanfacine/  Medetomidine/ Methyldopa/  Xylazine/ adrenergic agonist$  noradrenergic agonist$ norepinephrine  noradrenaline  levarterenol  levonoradrenaline  levonorepinephrine  levophed  levonor  arterenol  epinephrine  etilefrine  naphazoline  octopamine  oxymetazoline  phenylpropanolamine  synephrine  ergotamine  mephentermine  metaraminol  methoxamine  midodrine phenylephrine  clonidine  dexmedetomidine  guanabenz  guanfacine  medetomidine  methyldopa  xylazine  Adrenergic beta‐agonists/  Clenbuterol/  Epinephrine/  Isoproterenol/  Isoxsuprine/  Nylidrin/  Oxyfedrine/  Tretoquinol/  Adrenergic beta‐1 receptor agonists/  Dobutamine/  Etilefrine/  Prenalterol/  Xamoterol/  Adrenergic beta‐2 receptor agonists/  Albuterol/  Fenoterol/  Hexoprenaline/ Isoetharine/  Metaproterenol/  Procaterol/  Ritodrine/  Terbutaline/  Adrenergic beta‐3 receptor agonists/ clenbuterol  isoproterenol  isoxsuprine  nylidrin  oxyfedrine  tretoquinol  dobutamine  etilefrine  prenalterol  xamoterol  albuterol  fenoterol  hexoprenaline  isoetharine  metaproterenol  procaterol  ritodrine  terbutaline exp Drug Therapy/ drug  pharmacol$  therap$  treat$  effect$  pharmacotherap$  fluoxetine  rivastigmine | Randomized Controlled Trials as Topic/ Random allocation/ Controlled Clinical Trials as Topic/ Control groups/ Clinical trials as topic/ Clinical trials, phase I as topic/  Clinical trials, phase II as topic/  Clinical trials, phase III as topic/  Clinical trials, phase IV as topic/ Double‐blind method/ Single‐blind method/ Placebos/ Placebo effect/ Cross‐over studies/ Therapies, Investigational/ Drug Evaluation/ Research Design/ randomized controlled trial controlled clinical trial clinical trial clinical trial phase I  clinical trial phase II clinical trial phase III clinical trial phase IV random$  RCT  RCTs controlled trial$ controlled stud$ clinical$ trial$ control  treatment  experiment$  intervention  group$  subject$  patient$ quasi‐random$  quasi random$  pseudo‐random$  pseudo random$ experiment$ conservative  treatment  therapy  procedure  manage$ singl$  doubl$  tripl$  trebl$  blind$ mask$  cross‐over  cross over  crossover placebo$  sham  trial assign$  allocat$ controls |  |
| OR | OR | OR | OR |  |
| AND | | | | |

**PICO 13**

| Cerebrovascular disorders/  Brain ischaemia/  Intracranial Arterial Disease  Intracranial Arteriovenous Malformations/  Intracranial Embolism and Thrombosis/  Stroke/ | Eye Movements/  Eye/  Eye Disease/  Visually Impaired Persons/  Vision Disorders/  Blindness/  Diplopia/  Vision, Binocular/  Vision, Monocular/  Visual Acuity/  Visual Fields/  Vision, Low/  Ocular Motility Disorders/  Blindness, Cortical/  Hemianopsia/  Abducens Nerve Diseases/  Abducens Nerve/  Oculomotor Nerve/  Trochlear Nerve/  Visual Perception/  Nystagmus  strabismus  smooth pursuits  saccades  depth perception  stereopsis  gaze disorder  internuclear ophthalmoplegia  Parinaud’s syndrome  Weber’s syndrome  skew deviation  conjugate deviation  oscillopsia  visual tracking  agnosia  hallucinations |  |
| --- | --- | --- |
| Cerebrovascular disorders/  Basal ganglia cerebrovascular disease/  Brain ischemia  exp Carotid artery diseases/  exp Cerebral small vessel diseases/  exp Intracranial arterial diseases/  exp Intracranial embolism and thrombosis/  exp Intracranial hemorrhages/  Stroke/  exp Brain infarction/  Stroke, lacunar/  Vasospasm, intracranial/  Vertebral artery dissection/  Carotid stenosis/  exp Carotid artery injuries/  Intracranial arterial diseases/  Cerebral arterial diseases/  Infarction, anterior cerebral artery/ Infarction, middle cerebral artery/  Infarction, posterior cerebral artery/  exp Carotid arteries/ Endarterectomy, carotid/  stroke$  poststroke  apoplex$  cerebral vasc$  brain vasc$  cerebrovasc$  cva$  SAH  brain$  cerebr$  cerebell$  vertebrobasil$  hemispher$  intracran$  intracerebral  infratentorial  supratentorial  middle cerebral arter$  MCA$  anterior circulation  posterior circulation  basilar arter$  vertebral arter$  space‐occupying  isch?emi$  infarct$  thrombo$  emboli$  occlus$  hypoxi$ brain$  cerebr$  cerebell$  intracerebral  intracran$  parenchymal  intraparenchymal  intraventricular  infratentorial  supratentorial  basal gangli$  putaminal  putamen  posterior fossa  hemispher$  subarachnoid  hemorrhag$  hematoma$  bleed$ | exp Perceptual disorders/  exp Perception/ Vision disorders/  Alice in wonderland syndrome/  Amblyopia/  Blindness/  Blindness, cortical/  Color vision defects/ Diplopia/  Hemianopsia/  Photophobia/  Scotoma/  Vision, low/ percept$ impair$  percept$ problem$ percept$ abilit$ percept$ deficit$ percept$ distortion$ percept$ defect$ percept$ disabilit$ percept$ disturbance$ percept$ disorder$ percept$ discriminat$  agnosis  agnosia  prosopagnosia  prosophthalmia  Todd syndrome  allesthesia$  synesthesia$  hypoesthesia  hyperesthesia Vision, ocular/  exp Mesopic vision/ Night vision/  amblyop?  aniseikonia  oscillopsia xanthopsia  diplop$  polyop$  metamorphopsia micropsia  vision  visual  visualpercept$  visuospatial  visuoconstruct$ ocular  optokinetic  optic$  oculomotor spatial) illusion  blurry  overload  double  percept$  perceive$  discriminat$ distinguish$  recept$  sensitiv$  hallucination$ abnormalit$ distortion$ disturbance$  anomal$ disorientation  allachethesia  deficit$  defect$  disabilit$  disorder$  processing dysfunction$  recogn$  interpretation  analysis  comprehension stereoillusion kakopsia  kalopsia  pelopsia achromatopsia akinetopsia  telopsia  stereopsis  palinopsia  teleopsia simultanagnosia  entomopia palinopsia asteropsis strabismus  Anton syndrome Balint syndrome blindsight  Hyperchromatosis  fac$ intermetamorphosis  visual anoneria figure recogn$.  shape recogn$.  orientation recogn$.  form recogn$.  colo?r recogn$.  textur$ recogn$.  crowding recogn$.  contour recogn$.  object recogn$.  face recogn$.  faces recogn$. | Randomized controlled trial/ controlled clinical trial. randomized placebo  clinical trials as topic  random$ trial |
| OR | OR | OR |
| AND | | |

**Supplementary table 5.1: PICO 1: For adults with visual problems due to stroke, does routine use of vision screening, compared to no routine vision screening, improve detection rate?** **Study details reporting sensitivity and specificity of vision screening**

| **Author** | **Date** | **Country** | **Aim** | **Design** | **Sample** | **Vision screen** | **Reference test(s)** | **Sensitivity** | **Specificity** |
| --- | --- | --- | --- | --- | --- | --- | --- | --- | --- |
| **Pre-hospital** | | | | | | | | | |
| Aroor^32^ | 2017 | USA | Determine the current proportion of acute ischemic stroke patients with symptoms not captured by FAST and whether addition of gait-related or visual symptoms would improve detection rates | Retrospective review | 736 | BEFAST | FAST | 14.1% did not have FAST symptoms at presentation. In the FAST-negative group, the use of BEFAST significantly reduced the proportion of non-identified stroke to 4.4% | |
| Rowe^33^ | 2020 | UK | Profile the full range of visual disorders from a large prospective observation study of stroke survivors referred to by stroke multidisciplinary teams to orthoptic services with suspected vision problems | Prospective cohort | 43 | V-FAST | NIHSS | 85.7%  95%CI:  42.1 – 99.6% | 42.1%  95%CI:  20.3 – 66.5% |
| **Stroke units or out-patient clinics** | | | | | | | | | |
| Neumann^34^ | 2016 | Germany | Evaluate the clinical validity and psychometric qualities of the CVSQ for the anamnesis  of CVD in individuals poststroke | Cohort | 461 | CVSQ | Specialist visual assessment | Visual complaints | |
|  |  |  |  |  |  |  |  | 79.8%  Range:  59.8 – 96.5% | 81.7%  Range:  59.3 – 91.7% |
| Quinn^36^ | 2018 | UK | Validate visual assessments in stroke using our StrokeVision digital platform, including visual field assessments and visual inattention/neglect assessments | Diagnostic accuracy | 48 | StrokeVisionApp | Routine screen and specialist visual assessment | 71%  95%CI:  48 – 89% | 83%  95%CI:  64 – 95% |
| Rowe^38^ | 2018 | UK | Develop the new VISA tool intended for use by the stroke team to improve identification of visual impairment in stroke survivors | Diagnostic accuracy | 89 (of 116 total) | VISA | Specialist visual assessment | 90.24%  95%CI:  81.68 – 95.69% | 85.29%  95%CI:  68.94 – 95.05% |
| Rowe^35^ | 2020 | UK | Validate the new VISA tool intended for use by the stroke team to improve identification of visual impairment in stroke survivors | Diagnostic accuracy | 101 | VISA (print version) | Specialist visual assessment | 97.67%  95%CI:  91.85 – 99.72% | 60.00%  95%CI:  32.29 – 83.66% |
|  |  |  |  |  | 100 | VISA (app version) | Specialist visual assessment | 88.31%  95%CI:  78.97 – 94.51% | 86.96%  95%CI:  66.41 – 97.22% |
| Courtney-Harris^39^ | 2022 | Australia | Determine the sensitivity and specificity in the detection of pre-existing or acquired stroke-related visual defects | Diagnostic accuracy | 99 | SVDST | Specialist visual assessment | 91.1% *  95%CI:  86.4 – 94.5% | 92.57% *  95%CI:  88.8 – 95.4% |
| Wijesundera^37^ | 2022 | Australia | Utilize the MRFn iPad application to measure visual acuity with high contrast targets, visual acuity-in noise and visual field integrity in first episode hospitalized  ischemic acute stroke patients with no prior history of  ocular disorder | Case control | 60 | MRFn app | Specialist visual assessment | 93% | 83% |

* Data requested from authors for raw data values

BEFAST (balance, eyes, face, arm, speech, time); CVD (cerebrovascular disease); CVSQ (cerebral vision screening questionnaire); FAST (face, arm, speech, time); MRFn (Melbourne rapid field-neural); NIHSS (National Institute of Health Stroke Screen); SVDST (stroke vision defect screening tool); V-FAST (vision, face, arm, speech, time); VISA (vision impairment screening assessment).

**Supplementary table 5.2: PICO1 - Study details reporting detection rates for vision screening**

| **Author** | **Date** | **Country** | **Aim** | **Design** | **Sample** | **Vision screen** | **Detection rate** |
| --- | --- | --- | --- | --- | --- | --- | --- |
| Beschin^40^ | 2014 | Italy | Investigate the relationship between prose reading and other measures of unilateral spatial neglect, its severity and, between prose reading and single word naming | Cross-sectional | 30 | Specialist visual assessment | 70% - prose reading neglect  73.33% single word neglect |
| Benshir^41^ | 2016 | USA | Undertake vision evaluations and report results to the physiatrists and rehabilitation therapists, with the goal of enhancing the efficacy of therapeutic intervention | Cross-sectional | 200 | Specialist visual assessment | 96.5% visual impairment |
| Herron^42^ | 2016 | USA | Establish a profile for visual deficits following stroke including functional symptoms, diagnoses, and treatment recommendations on rehabilitation unit. Examine the correlation between number of functional deficits identified by occupational therapists and visual diagnoses and recommendations by the optometrist. Explore the role of occupational therapy in vision assessment and effectiveness of working in a partnership with an optometrist on rehabilitation unit | Cross-sectional | 677 | Occupational therapy observations | 19.4% visual impairment |
| Olubor^43^ | 2016 | Nigeria | Determine the prevalence of ocular disorders in patients with stroke in a tertiary hospital in Nigeria | Descriptive cohort | 85 | Specialist visual assessment | 95.5% visual impairment |
| Rowe^44^ | 2017 | UK | Profile the full range of visual disorders from a large prospective observation study of stroke survivors referred to by stroke multidisciplinary teams to orthoptic services with suspected vision problems | Prospective cohort | 915 | Specialist visual assessment | 92% visual impairment |
| Puig-Pijoan^45^ | 2018 | Spain | Describe unilateral spatial neglect in patients with non-dominant hemisphere stroke | Cross-sectional | 62 | Vision screen | 40.3% neglect |
| Dadia^46^ | 2019 | India | Review and understand the association between location of stroke and ophthalmic manifestations | Cross-sectional | 50 | Specialist visual assessment | 90% visual impairment |
| Hammerbeck ^47^ | 2019 | UK | Establish prevalence of spatial neglect and disease profile | Prevalence study | 88664 | Routine stroke screen | 30% neglect |
| Smith^51^ | 2019 | USA | Examine the utility of various visual screening measures and document the results of the screening tools for a cohort of persons with aphasia | Cohort | 23 | Vision screen | 65.22% visual impairment |
| Muratova^48^ | 2020 | Ukraine | Assess frequency of neuro-ophthalmological abnormalities in patients with stroke | Cross-sectional | 298 | Specialist visual assessment | 88.8% visual impairment |
| Rowe^33^ | 2020 | UK | Develop a quick vision screening tool using simple validated assessments of visual function that identify the most common visual impairments associated with stroke, coupled with a supporting education package | Cross-sectional | 43 | Vision screen | 60.5% visual impairment |
| Wijesundera^49^ | 2020 | Australia | Utilize the MRFn iPad application to measure visual acuity with high contrast targets, visual acuity-in noise and visual field integrity in first episode hospitalized  ischemic acute stroke patients with no prior history of ocular disorder | Case control | 60 | Vision screen | 68% visual impairment |
| Moon^50^ | 2021 | Korea | Describe the visual and oculomotor features of thalamic infarction and to delineate clinical outcomes and prognostic factors of the oculomotor deficits from an ophthalmologic point of view | Cohort | 342 | Specialist visual assessment | 11.7% visual impairment |
| Rowe^1^ | 2022 | UK | Determine the incidence and point prevalence of visual problems in an acute stroke population and to explore the timing at which visual assessment can be first undertaken in this population | Prospective epidemiology | 1204 (of 1500 total) | Specialist visual assessment | 73% - prevalence  60% - incidence visual impairment |

MRFn (Melbourne rapid field-neural).

**Supplementary table 6.1: PICO 2: For adults with visual problems due to stroke, does early assessment within one week of stroke admission, compared to later assessment, improve activities and quality of daily life?** **Study details reporting time of vision screen and length of stay**

| **Author** | **Date** | **Country** | **Aim** | **Design** | **Sample** | **Vision screen** | **Length of stay** | **Time to assessment** |
| --- | --- | --- | --- | --- | --- | --- | --- | --- |
| Norup^52^ | 2016 | Denmark | Describe establishment of interdisciplinary visual team and the role of the team in acute/subacute stroke unit; report frequency and type of visual deficits in acute stroke | Cohort | 349 – 22 vision screened, 14 with follow-up | Specialist visual assessment | 37.4 days (SD 27.2) | 81.8% referred to visual team at mean 8 days (SD 8.3) |
| Rowe^54^ | 2017 | UK | Consider the practice of orthoptists internationally in care provision for poststroke visual impairment though an international survey | Survey | 290 | Specialist visual assessment |  | 35.5% reporting first vision assessments within 2 weeks and 55.5% within 1 month |
| Raty^53^ | 2018 | Finland | Identify pre-hospital pathways and delays of occipital strokes presenting with mainly visual symptoms, investigate obstacles and factors with greater delays | Cross-sectional | 245 | Routine stroke screen |  | 20.8% seen within 4.5 hours |
| Rowe^2^ | 2019 | UK | Determine incidence and point prevalence of visual problems in an acute adult stroke population and explore feasibility of early timing of visual assessment | Epidemiology cohort | 1295 – 1033 vision screened | Specialist visual assessment | Normal visual assessments (mean 13.5 days, SD 45.9)  Visual impairment confirmed (mean 49.9 days, SD 68.3)  Greater severity of stroke (Barthel score, p = 0.0001) | Vision screen for 1033:  Mean 6.5 days (SD 24)  Median 3 days (IQR 2)  Full visual assessment:  Mean of 13.4 days (SD 33.8)  Median 4 days (IQR 7)  Visual assessment within the first 4 days of hospitalization in over 70% (n=824) of stroke population  90% of visual problems in stroke survivors diagnosed within first month post stroke |

**Supplementary table 7.1: PICO 3: For adults with visual field loss due to stroke, does identification of visual field loss by vision screening or specialist eye team, compared to routine stroke screen, improve detection rate and activities/quality of life?** **Study details reporting sensitivity and specificity of vision screening for visual field loss**

| **Author** | **Date** | **Country** | **Aim** | **Design** | **Sample** | **Vision screen** | **Reference test(s)** | **Sensitivity** | **Specificity** |
| --- | --- | --- | --- | --- | --- | --- | --- | --- | --- |
| **Pre-hospital** | | | | | | | | | |
| Karimi^38^ | 2020 | Iran | Compare the accuracy of PreHAST to CPSS in patients with a suspicion of stroke | Cross-sectional | 883 | PreHAST | CPSS | 5.3% | 98.1% |
| **Stroke units or out-patient clinics** | | | | | | | | | |
| Quinn^36^ | 2018 | UK | Validate visual assessments in stroke using the StrokeVision digital platform, including visual field assessments and visual inattention/neglect assessments | Diagnostic accuracy | 48 – 45 completing visual field screen | StrokeVisionapp | Routine screen and specialist visual assessment | Vs confrontation | |
|  |  |  |  |  |  |  |  | 71%  95%CI: 48-89% | 83%  95%CI: 64-95% |
|  |  |  |  |  |  |  |  | Vs perimetry | |
|  |  |  |  |  |  |  |  | 79%  95%CI: 54-94% | 88%  95%CI: 68-97% |
| Rowe^38^ | 2018 | UK | Develop a new VISA tool intended for use by the stroke team to improve identification of visual impairment in stroke survivors | Diagnostic accuracy | 116 – 89 completing visual field screen | VISA | Specialist visual assessment | 88.89% *  95%CI:  70.84 – 97.65% | 89.74% *  95%CI:  80.79 – 95.47% |
| Rowe^35^ | 2020 | UK | Validate a new VISA tool intended for use by the stroke team to improve identification of visual impairment in stroke survivors | Diagnostic accuracy | 101 | VISA (print version) | Specialist visual assessment | 82.05% *  95%CI:  66.47 – 92.46% | 70.97% *  95%CI:  58.05 – 81.80% |
|  |  |  |  |  | 100 | VISA (app version) | Specialist visual assessment | Vs confrontation | |
|  |  |  |  |  |  |  |  | 92.86% *  95%CI:  80.52 – 98.50% | 79.31% *  95%CI:  66.65 – 88.83% |
|  |  |  |  |  |  |  |  | Vs perimetry | |
|  |  |  |  |  |  |  |  | 100.00% *  95%CI:  83.89 – 100.00% | 100.00% *  95%CI:  39.76 – 100.00% |

* Data requested from authors for raw data values

CPSS (Cincinnati Prehospital Stroke Scale); PreHAST (Prehospital Ambulance Stroke Test); VISA (Visual Impairment Screening Assessment).

**Supplementary table 7.2: PICO 3 - Study details reporting detection rates for vision screening of visual field loss**

| **Author** | **Date** | **Country** | **Aim** | **Design** | **Sample** | **Vision screen** | **Detection rate of visual field loss** |
| --- | --- | --- | --- | --- | --- | --- | --- |
| Benshir^41^ | 2016 | USA | Undertake vision evaluations and report results to the physiatrists and rehabilitation therapists, with the goal of enhancing the efficacy of therapeutic intervention | Cross-sectional | 200 | Specialist visual assessment | 54% |
| Herron^42^ | 2016 | USA | Establish a profile for visual deficits following stroke including functional symptoms, diagnoses, and treatment recommendations on rehabilitation unit. Examine the correlation between number of functional deficits identified by occupational therapists and visual diagnoses and recommendations by the optometrist. Explore the role of occupational therapy in vision assessment and effectiveness of working in a partnership with an optometrist on rehabilitation unit | Cross-sectional | 677 | Occupational therapy observations | 25.2% |
| Mao^56^ | 2016 | China | Develop and validate a new stroke recognition instrument for differentiating acute stroke from stroke mimics in an emergency setting | Cross-sectional | 416 | Guangzhou stroke scale | 19% |
| Norup^52^ | 2016 | Denmark | Describe establishment of an interdisciplinary visual team and the role of the team in the acute and sub-acute phase at a hospital stroke unit, and to report the results of a preliminary prospective study investigating the frequency and type of visual and/or visuo-attentional deficits in patients with acute stroke | Cohort | 22 (of 349 total) | Specialist visual assessment | 45.4% |
| Berthold-Lindstedt^57^ | 2017 | Sweden | Examine and analyse the occurrence of self-reported visual changes in a Swedish out-patient group with medium to severe acquired brain injury | Cross-sectional | 170 | Vision screen | 27% |
| Rowe^44^ | 2017 | UK | Profile the full range of visual disorders from a large prospective observation study of stroke survivors referred to by stroke multidisciplinary teams to orthoptic services with suspected vision problems | Prospective cohort | 915 | Specialist visual assessment | 68% |
| Dadia^46^ | 2019 | India | Review and understand the association between location of stroke and ophthalmic manifestations | Cross-sectional | 50 | Specialist visual assessment | 38% |
| Rowe^2^ | 2019 | UK | Determine the incidence and point prevalence of visual problems in an acute stroke population and to explore the timing at which visual assessment can be first undertaken in this population | Prospective epidemiology | 1204 (of 1500 total) | Specialist visual assessment | 25.6% |
| Ambika^58^ | 2020 | India | Report the visual profile of patients with acquired brain injury who reported to a Neuro-Optometry clinic of a tertiary eye care center in India | Cross-sectional | 241 | Specialist visual assessment | 27% |
| Muratova^48^ | 2020 | Ukraine | Assess frequency of neuro-ophthalmological abnormalities in patients with stroke | Cross-sectional | 298 | Specialist visual assessment | 27.9% |
| Rowe^33^ | 2020 | UK | Develop a quick vision screening tool using simple validated assessments of visual function that identify the most common visual impairments associated with stroke, coupled with a supporting education package | Cross-sectional | 43 | Vision screen | 18.6% |
| Tharaldsen ^59^ | 2020 | Norway | Detect visual field defects after occipital infarction, investigate the rate of recovery and the impact of visual field defects upon vision-related quality of life | Cohort | 76 | Specialist visual assessment | 79% |
| Wijesundera^37^ | 2020 | Australia | Utilize the MRFn iPad application to measure visual acuity with high contrast targets, visual acuity-in noise and visual field integrity in first episode hospitalized ischemic acute stroke patients with no prior history of ocular disorder | Case control | 60 | Vision screen | 68% |
| Lee^60^ | 2022 | South Korea | Analyze and compare ocular manifestations, visual field pattern, and visual field test performance in traumatic brain injury and stroke patients | Cross-sectional | 118 | Specialist visual assessment | 65.2% |
| Nedumgattil^61^ | 2022 | India | Assess neuro-ophthalmological abnormalities in patients with stroke | Cross-sectional | 52 | Specialist visual assessment | 18.1% |

MRFn (Melbourne Rapid Field-neural).

**Supplementary table 8.1: PICO 4: For adults with central vision impairment due to stroke, does identification of visual acuity loss by vision screening or specialist eye team, compared to routine stroke screen, improve detection rate and activities/quality of life?** **Study details reporting sensitivity and specificity of vision screening for visual acuity loss**

| **Author** | **Date** | **Country** | **Aim** | **Design** | **Sample** | **Vision screen** | **Reference test(s)** | **Sensitivity** | **Specificity** |
| --- | --- | --- | --- | --- | --- | --- | --- | --- | --- |
| Neumann^34^ | 2016 | Germany | Evaluate the clinical validity and psychometric qualities of the CVSQ for the anamnesis of cerebro vascular disorders in individuals poststroke | Cohort | 461 | CVSQ | Specialist visual assessment | Reading problems | |
|  |  |  |  |  |  |  |  | 83.9% | 74.7% |
|  |  |  |  |  |  |  |  | Blurred vision | |
|  |  |  |  |  |  |  |  | 79.1% | 86.7% |
| Rowe^38^ | 2018 | UK | Develop a new VISA tool intended for use by the stroke team to improve identification of visual impairment in stroke survivors | Diagnostic accuracy | 89 (of 116 total) | VISA | Specialist visual assessment | Near visual acuity | |
|  |  |  |  |  |  |  |  | 78.72% *  95%CI:  64.34 – 89.30% | 88.89% *  95%CI:  78.44 – 95.41% |
|  |  |  |  |  |  |  |  | Distance visual acuity | |
|  |  |  |  |  |  |  |  | 82.61% *  95%CI:  68.58 – 92.18% | 94.92% *  95%CI:  85.85 – 98.94% |
| Rowe^35^ | 2020 | UK | Validate a new VISA tool intended for use by the stroke team to improve identification of visual impairment in stroke survivors | Diagnostic accuracy | 101 | VISA (print version) | Specialist visual assessment | Near visual acuity | |
|  |  |  |  |  |  |  |  | 67.61% *  95%CI:  55.45 – 78.24% | 58.62% *  95%CI:  38.94 – 76.48% |
|  |  |  |  |  |  |  |  | Distance visual acuity | |
|  |  |  |  |  |  |  |  | 81.63% *  95% CI:  67.98 – 91.24% | 75.00% *  95% CI:  61.05 – 85.97% |
|  |  |  |  |  | 100 | VISA (app version) | Specialist visual assessment | Near visual acuity | |
|  |  |  |  |  |  |  |  | 40.62% *  95%CI:  23.70 – 59.36% | 95.52% *  95%CI:  87.47 – 99.07% |
|  |  |  |  |  |  |  |  | Distance visual acuity | |
|  |  |  |  |  |  |  |  | 83.78% *  95%CI:  67.99 – 93.81% | 93.65% *  95%CI:  84.53 – 98.24% |

* Data requested from authors for raw data values

CVSQ (Cerebral Vision Screening Questionnaire); VISA (Visual Impairment Screening Assessment).

**Supplementary table 8.2: PICO 4 - Study details reporting detection rates for vision screening of visual acuity loss**

| **Author** | **Date** | **Country** | **Aim** | **Design** | **Sample** | **Vision screen** | **Detection rate of visual acuity loss** |
| --- | --- | --- | --- | --- | --- | --- | --- |
| Neumann^34^ | 2016 | Germany | Evaluate the clinical validity and psychometric qualities of the CVSQ for the anamnesis of cerebro vascular disorders in individuals poststroke | Cohort | 461 | Questionnaire | Reading difficulty 54%  Blurred vision 31.5% |
| Olubor^43^ | 2016 | Nigeria | Determine the prevalence of ocular disorders in patients with stroke in a Tertiary Hospital in Nigeria | Descriptive cohort | 85 | Specialist visual assessment | 32.4% |
| Rowe^44^ | 2017 | UK | Profile the full range of visual disorders from a large prospective observation study of stroke survivors referred to by stroke multidisciplinary teams to orthoptic services with suspected vision problems | Prospective cohort | 915 | Specialist visual assessment | 24% |
| Smith^51^ | 2019 | USA | Examine the utility of various visual screening measures and document the results of the screening tools for a cohort of persons with aphasia | Cohort | 23 | Vision screen | 43% |
| Rowe^33^ | 2020 | UK | Develop a quick vision screening tool using simple validated assessments of visual function that identify the most common visual impairments associated with stroke, coupled with a supporting education package | Cross-sectional | 43 | Vision screen | Reading difficulty 20.9% |
| Rowe^1^ | 2022 | UK | Determine the incidence and point prevalence of visual problems in an acute stroke population and to explore the timing at which visual assessment can be first undertaken in this population | Prospective epidemiology | 1204 (of 1500 total) | Specialist visual assessment | Prevalence 43.9%  Incidence 29.4% |

CVSQ (Cerebral Vision Screening Questionnaire).

**Supplementary table 9.1: PICO 5: For adults with eye movement disorders due to stroke, does identification of strabismus and/or ocular motility deficit loss by vision screening or specialist eye team, compared to routine stroke screen, improve detection rate and activities/quality of life?** **Study details reporting sensitivity and specificity of vision screening for eye movement disorders**

| **Author** | **Date** | **Country** | **Aim** | **Design** | **Sample** | **Vision screen** | **Reference test(s)** | **Sensitivity** | **Specificity** |
| --- | --- | --- | --- | --- | --- | --- | --- | --- | --- |
| **Pre-hospital** | | | | | | | | | |
| Rowe^33^ | 2020 | UK | Profile the full range of visual disorders from a large prospective observation study of stroke survivors referred to by stroke multidisciplinary teams to orthoptic services with suspected vision problems | Prospective cohort | 43 | V-FAST | NIHSS | V-FAST detected eye movement problems in 27.9% vs 15.4% on the NIHSS horizontal gaze item | |
| **Stroke units or out-patient clinics** | | | | | | | | | |
| Rowe^38^ | 2018 | UK | Develop a new VISA tool intended for use by the stroke team to improve identification of visual  impairment in stroke survivors. | Diagnostic accuracy | 89  86 fully completed | VISA | Specialist visual assessment | 16.00% *  95%CI:  5.54 – 36.08% | 93.41% *  95%CI:  86.20 – 97.54% |
| Rowe^35^ | 2020 | UK | Validate new VISA tool intended for use by the stroke team to improve identification of visual  impairment in stroke survivors. | Diagnostic accuracy | 101 | VISA (print version) | Specialist visual assessment | 66.67% *  95%CI:  47.19 – 82.71% | 73.24% *  95%CI:  61.41 – 83.06% |

* Data requested from authors for raw data values

NIHSS (National Institute for Health stroke scale); V-FAST (vision, face, arm, speech, time); VISA (vision impairment screening assessment)

**Supplementary table 9.2: PICO 5 - Study details reporting detection rates for vision screening of eye movement disorders**

| **Author** | **Date** | **Country** | **Aim** | **Design** | **Sample** | **Vision screen** | **Detection rate** |
| --- | --- | --- | --- | --- | --- | --- | --- |
| Kerber^63^ | 2015 | USA | Estimate the ability of bedside information (ABCD2 score, general neurological examination and specialised oculomotor examination) to stratify stroke risk in acute dizziness presentations | Cross-sectional | 272  29 were stroke | Specialist visual assessment | 59% overall  24/29 (82.7%) for stroke |
| Norup^52^ | 2016 | Denmark | Describe establishment of an interdisciplinary visual team and the role of the team in the acute and sub-acute phase at a hospital stroke unit, and to report the results of a preliminary prospective study investigating the frequency and type of visual and/or visuo-attentional deficits in patients with acute stroke | Cohort | 349 | Specialist visual assessment | 27.2% |
| Rowe^44^ | 2017 | UK | Profile the full range of visual disorders from a large prospective observation study of stroke survivors referred to by stroke multidisciplinary teams to orthoptic services with suspected vision problems | Prospective cohort | 915 | Specialist visual assessment | 68% |
| Kim^64^ | 2019 | South Korea | Elucidate eye movement abnormalities in lesions confined to middle cerebellar peduncle | Cohort | 23 | Specialist visual assessment | 78% |
| Dadia^46^ | 2019 | India | Review and understand the association between location of stroke and ophthalmic manifestations | Cross-sectional | 50 | Specialist visual assessment | 62% |
| Pimentel^65^ | 2019 | Brazil | Investigate vestibular and oculomotor functions in patients with dizziness after stroke, and to compare the results with vestibular and visual symptoms | Cross-sectional | 50 | Specialist visual assessment | 56% |
| Rowe^33^ | 2020 | UK | Develop a quick vision screening tool using simple validated assessments of visual function that identify the most common visual impairments associated with stroke, coupled with a supporting education package | Cross-sectional | 43 | Vision screen | 27.9% |
| Rowe^1^ | 2022 | UK | Determine the incidence and point prevalence of visual problems in an acute stroke population and to explore the timing at which visual assessment can be first undertaken in this population | Prospective epidemiology | 1204 | Specialist visual assessment | 44.3% - prevalence  39.3% - incidence |

ABCD2 (Age, Blood pressure, Clinical, Duration, Diabetes mellitus).

**Supplementary table 10.1: PICO 6: For adults with visual perceptual disorders due to stroke, does identification of visual perceptual disorders by screening proforma/tool or specialist team, compared to routine stroke screen, improve detection rate and activities/quality of life? Study details reporting sensitivity and specificity of vision screening for visual perceptual disorders**

| **Author** | **Date** | **Country** | **Aim** | **Design** | **Sample** | **Vision screen** | **Reference test(s)** | **Sensitivity** | **Specificity** |
| --- | --- | --- | --- | --- | --- | --- | --- | --- | --- |
| Neumann^34^ | 2016 | Germany | Evaluate the clinical validity and psychometric qualities of the CVSQ for the anamnesis of cerebro vascular disorders in individuals poststroke | Cohort | 461 | CVSQ | Specialist visual assessment | Depth/reaching | |
|  |  |  |  |  |  |  |  | 86.4% | 86.0% |
|  |  |  |  |  |  |  |  | Dark vision | |
|  |  |  |  |  |  |  |  | 59.8% | 90.9% |

CVSQ (Cerebral Vision Screening Questionnaire).

**Supplementary table 10.2: PICO 6 - Study details reporting detection rates for vision screening for visual perceptual disorders**

| **Author** | **Date** | **Country** | **Aim** | **Design** | **Sample** | **Vision screen** | **Detection rate** |
| --- | --- | --- | --- | --- | --- | --- | --- |
| Neumann^34^ | 2016 | Germany | Evaluate the clinical validity and psychometric qualities of the CVSQ for the anamnesis  of cerebro vascular disorders in individuals poststroke | Cohort | 461 | Questionnaire | Dark vision 16.1%  Colour perception 6.9%  Visual hallucinations 16.9%  Reaching objects 16.9% |
| Prince^67^ | 2017 | Canada | Identify which variables best explain the persistence of visual perceptual disorders in seniors with stroke | Cohort | 195 | Specialist visual assessment  Motor-free visual perceptual test | 50.8% |
| Rowe^44^ | 2017 | UK | Profile the full range of visual disorders from a large prospective observation study of stroke survivors referred to by stroke multidisciplinary teams to orthoptic services with suspected vision problems | Prospective cohort | 915 | Specialist visual assessment | 4.6% with visual perceptual disorders (excluding visual neglect) |
| Dadia^46^ | 2019 | India | Review and understand the association between location of stroke and ophthalmic manifestations | Cross-sectional | 50 | Specialist visual assessment | 6% |
| Lammers^68^ | 2022 | The Netherlands | Establish the prevalence and clinical characteristics of deficits in eight important ‘mid-range’ visual functions, and evaluate their co-occurrence in a large prospective cohort of stroke patients | Cohort | 220 | Specialist visual assessment  Gaze-contingent display | Deficits in:  Motion-perception 26%  Colour 22%  Texture 22%  Location 21%  Orientation 18%  Contrast 14%  Shape 14%  Glossiness 13% |
| Rowe^1^ | 2022 | UK | Determine the incidence and point prevalence of visual problems in an acute stroke population and to explore the timing at which visual assessment can be first undertaken in this population | Prospective epidemiology | 1204 (of 1500 total) | Specialist visual assessment | 4.9% |
| van den Berg^69^ | 2022 | The Netherlands | Investigate how deficits in a broad range of mid-range visual functions (i.e., color, shape, location, orientation, contrast, texture and motion) are associated with performance on neuropsychological tasks assessing higher-order cognitive functions, requiring an advanced processing of visual information (i.e., facial emotion recognition, visual recognition memory and visuoconstructive functioning), and whether these associations are in line with the two pathway model or with an alternative patchwork model | Cohort | 182 | Specialist visual assessment | Mid-range disorders 17.68%  Higher-order 22.83% |

CVSQ (Cerebral Vision Screening Questionnaire).

**Supplementary table 11.1: PICO 7. For adults with visual neglect due to stroke, does identification of visual neglect by screening proforma/tool or specialist team, compared to routine stroke screen, improve detection rate and activities/quality of life?Study details reporting sensitivity and specificity of vision screening for visual neglect**

| **Author** | **Date** | **Country** | **Aim** | **Design** | **Sample** | **Vision screen** | **Reference test(s)** | **Sensitivity** | **Specificity** |
| --- | --- | --- | --- | --- | --- | --- | --- | --- | --- |
| **Vs routine stroke screen** | | | | | | | | | |
| Grech^70^ | 2017 | Australia | Investigate the relationship between basic visual function  b and MAC performance. To investigate the diagnostic utility of the MAC for the assessment of neglect | Case control | 67 | MAC | Routine stroke screen | Total scores | |
|  |  |  |  |  |  |  |  | 74.2% | 69.4% |
|  |  |  |  |  |  |  |  | Vs star cancellation | |
|  |  |  |  |  |  |  |  | 43.3% | 94.4% |
|  |  |  |  |  |  |  |  | Vs line bisection | |
|  |  |  |  |  |  |  |  | 35.7% | 100% |
| Ten Brink^71^ | 2018 | The Netherlands | Assess the feasibility of the MAC, a visual search multitask, to assess neglect, and its relation with existing neglect tasks | Cohort | 113 | MAC | Routine stroke screen | Vs shape cancellation | |
|  |  |  |  |  |  |  |  | 82.8% | 90.5% |
|  |  |  |  |  |  |  |  | Vs line bisection | |
|  |  |  |  |  |  |  |  | 81.0% | 81.4% |
|  |  |  |  |  |  |  |  | Vs Catherine Bergego scale | |
|  |  |  |  |  |  |  |  | 70.7% | 96.0% |
| Moore^72^ | 2019 | UK | Evaluate the diagnostic sensitivity of the NIHSS’ visual neglect item compared with a brief neuro psychological cancellation test and to identify factors which modulate this sensitivity | Diagnostic accuracy | 428 | Oxford Cognitive screen | Routine stroke screen | 91.2% | 31.6% |
| Spreji^73^ | 2020 | The Netherlands | Use of a simulated driving task was used to assess (1) differences in performance between patients with left- and right-sided VSN, recovered VSN, without VSN, and healthy participants; (2) the relation between average position and VSN severity; and (3) its diagnostic accuracy in relation to traditional tasks | Cohort | 100 | Driving performance | Routine stroke screen | 52% | 94.3% |
| **Vs other screen or specialist visual assessment** | | | | | | | | | |
| Rowe^38^ | 2018 | UK | Develop a new VISA tool intended for use by the stroke team to improve identification of visual impairment in stroke  survivors | Diagnostic accuracy | 84 (of 116 total) | VISA (pilot) | Specialist visual assessment | 87.5% *  95%CI:  47.35-99.68% | 78.95% &  95%CI:  68.08-87.46% |
| Rowe^35^ | 2020 | UK | Validate a new VISA tool intended for use by the stroke team to improve identification of visual impairment in stroke i survivors | Diagnostic accuracy | 99 (of 101 total) | VISA (print version) | Specialist visual assessment | 88.24% *  95%CI:  72.55-96.70 | 67.69% *  95%CI:  54.95-78.77 |
|  |  |  |  |  | 100 | VISA (app version) | Specialist visual assessment | 60.0% *  95%CI:  32.29-83.66 | 81.18% *  95%CI:  71.24-88.84 |
| Kaufman^74^ | 2020 | Switzerland | Explore if video-oculography during free visual exploration, which can be performed in few minutes, is sensitive in mirroring neglect in everyday behaviour and whether it is more sensitive than conventional neuropsychological paper-pencil tests | Diagnostic accuracy | 78 | Video-oculography | Catherine Bergego scale | Mean gaze position | |
|  |  |  |  |  |  |  |  | 85.0% | 94.4% |
|  |  |  |  |  |  |  |  | Early orientation | |
|  |  |  |  |  |  |  |  | 83.3% | 61.1% |
| Bieth^75^ | 2021 | France | Validate a new test for early assessment of unilateral neglect in acute stroke: The RUNS test | Diagnostic accuracy | 75 | RUNS test | BEN test | 95%  95%CI:  89-100% | 80%  95%CI:  63-97% |
| Motomura^76^ | 2022 | Japan | To examine the accuracy, reliability, and validity of a new method to assess stimulus-evoked attention function in unilateral spatial neglect | Diagnostic accuracy | 44 | Simulus-driven attention test | Catherine Bergego scale | 65.13% | 94.75% |

* Data requested from authors for raw data values

BEN (Batterie d’Évaluation de la Négligence spatiale unilatérale); MAC (Mobility Assessment Course); NIHSS (National Institutes of Health Stroke Scale), RUNS (Rapid Unilateral Neglect Screening); VISA (Visual Impairment Screening Assessment); VSN (Visual Spatial Neglect).

**Supplementary table 11.2: Study details reporting detection rates for vision screening for visual neglect**

| **Author** | **Date** | **Country** | **Aim** | **Design** | **Sample** | **Vision screen** | **Detection rate** |
| --- | --- | --- | --- | --- | --- | --- | --- |
| Norup^52^ | 2016 | Denmark | Describe establishment of an interdisciplinary visual team and the role of the team in the acute and sub-acute phase at a hospital stroke unit, and to report the results of a preliminary prospective study investigating the frequency and type of visual and/or visuo-attentional deficits in patients with acute stroke | Cohort | 22 (of 349 total) | Specialist visual assessment | 31.8% |
| Sperber^77^ | 2016 | Germany | To test whether the line bisection task can provide a valid diagnosis of visual field defects and spatial neglect in the acute phase of stroke | Diagnostic accuracy | 180 | Routine screen | 46% |
| Rowe^44^ | 2017 | UK | Profile the full range of visual disorders from a large prospective observation study of stroke survivors referred to by stroke multidisciplinary teams to orthoptic services with suspected vision problems | Prospective cohort | 915 | Specialist visual assessment | 15% |
| Puig-Pijoan^45^ | 2018 | Spain | Describe unilateral spatial neglect in patients with non-dominant hemisphere stroke | Cross-sectional | 62 | Vision screen | 40.3% |
| Ten Brink^71^ | 2018 | The Netherlands | Assess the feasibility of the MAC, a visual search multitask, to assess neglect, and its relation with existing neglect tasks | Cohort | 113 | Vision screen | 34.6% |
| Van der Stigchel^78^ | 2018 | The Netherlands | Relate performance on the temporal order judgement test to performance on line bisection and cancellation tests | Cohort | 73 | Stroke screen | 21.9% |
| Moore^72^ | 2019 | UK | Evaluate the diagnostic sensitivity of the NIHSS’ visual neglect item compared with a brief neuropsychological cancellation test and to identify factors which modulate this sensitivity | Diagnostic accuracy | 428 | Vision screen | 46.5% |
| Gerafi^79^ | 2020 | Sweden | Investigate presence and predictors for signs of lateralized inattention 7 years after stroke | Cohort | 188 | Stroke screen | 11.7% |
| Kaufman^74^ | 2020 | Switzerland | Explore if video-oculography during free visual exploration, which can be performed in few minutes, is sensitive in mirroring neglect in everyday behaviour and whether it is more sensitive than conventional neuropsychological paper-pencil tests | Diagnostic accuracy | 78 | Vision screen | 76.92% |
| Rowe^35^ | 2020 | UK | Validate a new VISA tool intended for use by the stroke team to improve identification of visual impairment in stroke survivors | Diagnostic accuracy | 101 (of 201 total) | Vision screen | 26.2% |
| Bieth^75^ | 2021 | France | Validate a new test for early assessment of unilateral neglect in acute stroke: The RUNS test | Diagnostic accuracy | 75 | Vision screen | 73.3% |
| Gillen^80^ | 2021 | USA | Determine the prevalence and functional impact of unilateral spatial neglect on right hemisphere stroke patients in an inpatient rehabilitation facility using a multidimensional, functionally based behavioral measure (Catherine Bergego Scale) | Cohort | 742 | Vision screen | 86.2% |
| Rowe^1^ | 2022 | UK | Determine the incidence and point prevalence of visual problems in an acute stroke population and to explore the timing at which visual assessment can be first undertaken in this population | Prospective epidemiology | 1204 (of 1500 total) | Specialist visual assessment | 26.2% |

MAC (Mobility Assessment Course); NIHSS (National Institutes of Health Stroke Scale), RUNS (Rapid Unilateral Neglect Screening); VISA (Visual Impairment Screening Assessment).

**Supplementary table 12: PICO 8: For adults with homonymous visual field loss due to stroke, does compensatory, substitute or restitutive intervention, compared to no intervention, improve activities and quality of daily life? Intervention results.**

| **Compensatory interventions** | | | | | | | | |
| --- | --- | --- | --- | --- | --- | --- | --- | --- |
| **Author** | **Date** | **Country** | **Aim** | **Design** | **Sample** | **Intervention** | **Control** | **Results** |
| de Haan^83^ | 2016 | The Netherlands | Examine the effects of a compensatory scanning training  program using horizontal scanning on mobility-related activities and participation in daily life | RCT | 26 intervention  23 waiting list  25 healthy controls | Horizontal eye movement scanning.  15 individual sessions of 60–90 minutes each, 18.5 hours of face-to-face training in total during a period of 10 weeks.  Scanning strategy consisting of a triad of horizontal saccades | Waiting list control | Self-reported improvements after training were found, accompanied by improvements in detecting peripheral stimuli and avoiding obstacles during walking, especially in dual task situations in which a second task limits the attentional capacity available for compensatory scanning. Training only improved mobility-related activities in which detection of peripheral stimuli is important, while no improvement was found on tests that require other visual skills, such as reading, visual counting and visual search |
| Rowe^84^ | 2016 | UK | Compare visual rehabilitation interventions with NHS standard care, in patients with hemianopia following stroke. We wished to explore whether visual rehabilitation was more effective than standard care (advice only) at improving functional outcome in patients with hemianopia following stroke and whether prism therapy or visual search therapy was more effective at improving functional outcome in patients with hemianopia following stroke | RCT | 87  27 prisms  30 VST  30 controls  Completions:  25 prisms  24 VST  22 controls | Treatment A: Sector Fresnel prisms of 40 prism dioptre strength on their glasses (or plain glasses if not already worn). Participants were advised to wear the prisms for a minimum of 2 hours daily, for a minimum 6 weeks, from prism affixation.  Treatment B: Visual search training comprising an A4 landscape card with horizontal and diagonal numbered circles radiating out from a central fixation target | Treatment C: Control—standard care (information only) | Relative change in visual field area was 0.05, SD 0.14 (not significant). No evidence of differences across arms was found for any of the other secondary outcomes, including functional mobility (ANCOVA p=0.36, extended daily level index (ANCOVA p=0.93), EQ-5D visual analogue score (ANCOVA p=0.60), change of general health status (ANCOVA p=0.51), reading speed and reading accuracy. A significant effect was seen for quality of life measured with NEI-VFQ-25 questionnaire for the visual search training in comparison to the other two groups. Related adverse events were common with Fresnel prisms (69.2%), typically headaches |
| Crotty^85^ | 2018 | Australia | Compare the effectiveness of a standardised program versus current individualised therapy in patients with homonymous hemianopia | RCT | 24  13 VST  11 usual care  Completed:  10 VST  10 usual care | Sstandardised program which consisted of three weeks (9 sessions) static scanning training using the NVT scanning device and four weeks (12 sessions) of mobility training using NVT scanning techniques: pattern of systematic visual search strategies, defining the perimeter of the affected visual field as a starting point for consistent visual search patterns | Usual care, including one-to-one occupational therapy and mobility instruction promoting visual scanning and mobility training by the Guide Dogs Association | No significant differences were found between intervention groups for the primary outcome measure of scanning ability whilst walking at 7 weeks and at 3 months (p>0.05). No significant change in reading speed at 7 weeks compared to the control group. However, at 3 months significant differences were found for the NEI VFQ-25 total score (p=0.03) and dependency sub-score (p=0.03) measures |
| Sahraie ^93^ | 2019 | UK | Report on changes in performance of a large group of hemianopic patients who undertook NEC outside a clinic environment | Cross-sectional | 296  294 included | NEC visual search.  NEC contains 12 levels, with 4 levels at each of pop-out, complex, and conjunction search categories, hence the task difficulty is systematically increased as therapy progresses.  The patient’s task was to indicate whether or not a specific target was present by pressing one of two mouse buttons. Training up to 3 episodes of 15 min training per day and for at least 5 days per week | N/A | Patients’ performance on two objective (visual search times and errors) and one subjective (self-reported disability) measures of performance were assessed before and after therapy. The disability score at pre-therapy (mean18.68, SD 7.14) was higher than that on post-therapy (mean15.47, SD 6.14) and this difference was significant [t(253) = 8.70, p<0.001, Cohen’s d = 0.546]. Overall, 66% (167/254) reported a subjective improvement in their level of disability after the therapy and the reduction in disability score from pre-training (mean 26.44, SD 4.77) compared to post-training (mean 19.70, SD 6.3) was significant [t(92) 9.623,p<0.001. Effect depended on the initial disability score. |
| Dehn^86^ | 2020 | Germany | Examine the effects of training in a VR supermarket on cognitive functions,  depressive symptoms, and subjective cognitive complaints in patients with hemianopia/quadrantanopia and healthy controls | RCT | 20 participants  20 healthy controls | VR supermarket scanning training. required the participants to learn a shopping list (learning task) and to subsequently buy the respective products of this shopping list in a VR supermarket environment (supermarket task)  14 sessions over 8 days | As for the intervention | Stroke survivors with hemi- or quadrantanopia and matched healthy controls showed enhanced performances after the training than before the training. These improvements involved different cognitive sub-functions such as visual scanning, mental rotation, visuo construction, and cognitive flexibility. VR training differentially affected the rehabilitation of visual memory skills with a particular benefit for the patient group (training x group interaction) |
| Szalados^94^ | 2020 | UK | Investigated the clinical effectiveness of Eye-Search, a web-based therapy app designed to improve visual search times, in a large group of patients with either hemianopia, neglect or both hemianopia and neglect | Cohort | 426 | Eye-Search web-based training.  A ramp-step pursuit paradigm in which participants have to follow a “rolling ball” stimulus that contains a “C” shape and moves in two phases. The subjects’ goal is to find the ball’s resting place. The therapy moves through 16 progressively harder levels of 300 trials as static distractor stimuli are added in, and the contrast between the target and background is reduced | N/A | Significant three-way interaction between therapy, side and group. Eye-Search therapy improved search times to the affected visual field of patients with either hemianopia alone or neglect and hemianopia, but not those with neglect alone. Effect sizes were moderate to large. Significant interaction between therapy and group for the patient-reported outcome measure “finding things” that most closely matched the impairment-based outcome (visual search).  For three of the ADL scores, there was no therapy by group interaction but there was a significant main effect of therapy for “Shopping”, “Meal Preparation” and, “Collisions” |
| **Restitutive interventions** | | | | | | | | |
| Elshout^87^ | 2016 | The Netherlands | Compared the effectiveness of training with visual point stimuli to optic flow stimuli that effectively stimulate the visual motion sensitive regions | RCT  Cross-over design | 27 | Home-based training. Viewing distance was fixed at 40 cm. The subject’s face was indirectly lighted for eye tracking with the web cam. The computer was prepared with eye tracking software, and training programs that were adjusted to the particular visual field defect of the patient. Each patient served as its own control in a double (defect and intact) training paradigm. During defect training, high contrast stimuli (C > 0.9) were offered within the field defect along its border. During intact training, stimuli were presented within the intact field at about the same eccentricities as for the patient’s defect training. During stimulus presentation patients shifted attention covertly (without shifting eye fixation) toward the stimulus and responded using the keyboard. Only the fixation point was shown during the intertrial interval of 2 seconds. Trained 1 hour a day, 5 days a week during 8 weeks to complete at least 40 hours of training per hemifield |  | Visual discrimination training reduced the field defect. Goldmann perimetry revealed a statistically significant reduction of the visual field defect after the test training, but not after the control training or after no intervention. For both training rounds combined, Humphrey perimetry revealed that the effect of a directed training (sensitivity change in trained hemifield) exceeded that of an undirected training (sensitivity change in untrained hemifield). The interaction between trained and tested hemifield was just above the threshold of significance (p=0.058). Reading speed improved significantly for defect and intact training. |
| Bergsma^88^ | 2017 | The Netherlands | Determine if early start of visual restitution therapy improves visual field loss after stroke | RCT  Cross-over design | 17 subacute stroke participants  7 chronic stroke participants  30 completed | Training 1 hour a day, 5 days a week during both training rounds and completed at least 40 hours of training per training round. High contrast stimuli (C > 0.9) were presented for 5 seconds in the border area of the region of the visual field defect that is trained. If the dot was detected, the patient had to report (key press) whether a clockwise or counter-clockwise position was seen relative to the reference line. No response was given if no dot was detected | Training one ­half of the defect for 8 weeks, while monitoring spontaneous recovery in the other (control) half of the defect.  Next, trained and control regions were swapped, and training continued for another 8  weeks | In both groups, field stability was assessed during a no intervention period. Defect reduction was significantly greater in the trained part of the defect than in the simultaneously untrained part of the defect irrespective of training onset (p=0.001). In subacute patients, training contributed about twice as much to their defect reduction as the spontaneous recovery. Goal Attainment Scores were significantly and positively correlated with the total defect reduction (p=0.01), percentage increase reading speed was significantly and positively correlated with the defect reduction induced by training (epoch 1: p=0.0044; epoch 2: p=0.023). |
| Cavanagh^89^ | 2017 | USA | Assess if visual discrimination training improves performance on visual perimetry tests in chronic stroke patients with visual cortex involvement | Cohort | 17 intervention  5 untrained | Patients trained on left-right direction discrimination of random dot stimuli (n=6), vertical-horizontal orientation discrimination of static Gabors (n=5), or both tasks (n=6) at non overlapping, blind field locations. Training locations were chosen as sites where performance first dropped to chance (50% correct) during blind field border mapping.  5 days/week; 300 Trials/day, at home | Untrained | PMD in the 24-2 HVFs increased in trained patients by 1.2 +/- 0.29 dB vs decreases in untrained patients of 0.06 +/- 0.14 dB . Correlation PMD change with computed area of improvement: r=0.9224. After training, luminance detection sensitivity improved by at least 6 dB over 108.1 (SD 22.8) degrees^2^ untrained control: 16 degrees^2^) (independent t test, unequal variance: t17.5 = 17.49, p=0.001, CI95= +/- 49.4 degrees^2^. Sensitivity improvement trained up to 28dB (9.48 +/- 0.4 dB) versus untrained up to 14.5 dB (7.3+/-0.1 dB) (independent t test: p=0.003.  86% of HVF improvement occurred where pretraining sensitivity was between 3 and 18dB. Improvements dropped almost linearly above 15dB, with only around 10% occurring where pretraining sensitivity was above 18dB |
| Cavanagh^90^ | 2021 | USA | Does psycho physical training within hemianopic fields result in greater PMD improvements on 24-2 HVF than identical training within the sighted visual field? | RCT | 48  25 intervention  23 sham  46 completed:  23 intervention  23 sham | Training was performed at home for six months, consisting of repeated visual discriminations at a single location for 20-30 minutes per day. The study intervention was direction discrimination training in either the deficit or sighted visual hemifields. Stimuli consisted of 100% coherent black dots presented on a mid-grey background within a 5° diameter aperture. Subjects responded using arrows on a keyboard to indicate the perceived direction of motion. Auditory feedback was provided on every trial to indicate correct and incorrect responses | For subjects randomized to the Sighted-field training arm, their training location was the mirror-symmetric location in their sighted field and for the same treatment period as the intervention arm | Changes in PMD on Humphrey Visual Field Analyzer in both eyes. Mean PMDs improved over 6 months in Deficit-trained subjects (mean change OD 0.58dB,; mean change OS 0.84 dB, ). No improvement was observed in Sighted-trained subjects (mean change OD 0.12dB,; mean change OS 0.10dB). However, there were no significant differences between the alternative training modalities (OD: p=0.19; OS: p=0.10).  No significant training effect on HVF PMD differences compared to identical training within the visually intact field |
| El Nahas^91^ | 2021 | Egypt | Study the effect of navigated rTMS applied to perilesional areas on the recovery of patients with cortical visual field defects | RCT | 32  21 active group, 11 sham | 16 sessions of active or sham high frequency navigated perilesional rTMS that were scheduled to be every other day. Each of the four predetermined perilesional targets was stimulated for 4 consecutive sessions of 1000 pulses each, for a total of 4000 pulses per target point, then shifting to the next target until all 4 were covered over 16 sessions | Patients in the sham group received similar pattern of stimulation yet using a sham coil. It is magnetically shielded, yet optically identical to the active coil, producing sounds and sensations similar to the active coil without expected therapeutic effects | The active group showed statistically significant improvement in mean deviation values post-stimulation for the right and left eyes (p=0.008 and 0.001; respectively). There was statistically significant improvement of the visual field index values of the right and left eyes (p=0.002 and 0.02; respectively). NEI VFQ-25 values showed a trend towards improvement in the active group (p=0.06). None of the measured parameters showed significant improvement in the sham group |
| Raty^92^ | 2021 | Finland | Explore different non-invasive brain stimulation modalities for rehabilitation of hemianopia after chronic stroke | RCT | 56 | The three experiments were: i) repetitive transorbital alternating current stimulation (rtACS, n=8) vs. rtACS with prior cathodal transcranial direct current stimulation over the intact visual cortex (tDCS/rtACS, n=8) vs. sham (n=8); ii) rtACS (n=9) vs. sham (n=9); and iii) tDCS of the visual cortex (n=7) vs. sham (n=7).  The treatment comprised 10 NIBS sessions with 20–40 min daily stimulation within a two-week period (weekends off).  The stimulation was delivered for 20 min daily with the current amplitude of 1.5 mA, clearly above the phosphene threshold.  tDCS/rtACS: Cathodal tDCS was applied on the intact hemisphere for 10 min immediately prior to rtACS to “prime” the damaged hemisphere for the rtACS effect.  Experiment 2 rtACS: The stimulation was delivered for 30 min during treatment days 1–5 and for 40 min during days 6–10. Stimulation was delivered sequentially at each stimulation electrode.  Experiment 3 tDCS: tDCS was delivered as bilateral, dual-mode stimulation over the visual cortex. tDCS was delivered with fixed 2-mA amplitude for 20 min daily (30-s fade-in) | Experiment 1; The rtACS-sham condition comprised occasional current bursts (one 5 Hz burst every 1 min) at 100% of the phosphene threshold that induced weak phosphenes to ensure blinding. In the tDCS-sham condition, current was ramped up for 30 s, then stopped, and at the end of the session ramped down for another 30s, which enabled eliciting few cutaneous sensations.  Experiment 2: The sham condition comprised occasional current bursts of ten pulses (one 5-Hz burst every 5 min) at 100% of the phosphene threshold to induce weak phosphenes to ensure blinding.  Experiment 3: The sham condition was identical to the one in Experiment 1 | Primary outcomes in Experiments 1 and 2 were negative. Only significant between-group change was observed in Experiment 3, where tDCS increased visual field of the contralesional eye compared to sham. tDCS/rtACS improved dynamic vision, reading, and visual field of the contralesional eye, but was not superior to other groups. rtACS alone increased foveal sensitivity, but was otherwise ineffective. All trial-related procedures were tolerated well. This exploratory trial showed safety but no main effect of NIBS on vision restoration after stroke. However, tDCS and combined tDCS/rtACS induced improvements in visually guided performance that need to be confirmed in larger-sample trials |

ADL (activities of daily living); HVF (Humphrey visual fields); NEC (Neuro Eye Coach); NEI VFQ (National Eye Institute Visual Function Questionnaire); NIBS (non-invasive electrical brain stimulation); NVT (Neuro Vision Technology); RCT (randomised controlled trial); rtACS (repetitive transorbital Alternating Current Stimulation); tDCS (transcranial Direct Current Stimulation); VR (virtual reality).

**Supplementary table 13: PICO 9: For adults with ocular stroke, does compensatory, substitute or restitutive intervention, compared to no intervention, improve activities and quality of daily life?** **Intervention results.**

| **Author** | **Date** | **Country** | **Aim** | **Design** | **Sample** | **Intervention** | **Control** | **Results** |
| --- | --- | --- | --- | --- | --- | --- | --- | --- |
| Schultheiss^97^ | 2018 | Germany | Report on visual outcomes and safety of IVT and propose a sample size calculation for a multicentre prospective randomized placebo-controlled phase III trial | Cohort | 20 intervention  40 control | rTPA | Standard care: EAGLE CST-arm | 20 patients received IVT within 4.5 hours after NA-CRAO with a median onset-to-treatment time of 210 minutes (IQR 120–240). Visual acuity improved in the intervention group (baseline visual acuity of 2.46 ±0.33 to 1.52 ±1.09 at day 5 and 1.60 ±1.08 logMAR at day 30) and in standard care (baseline visual acuity of 2.09 ±0.51 to 1.78 ±0.60 at day 5 and 1.63 ±0.62 logMAR at day 30) with no significant differences between groups at final follow-up. functional recovery to reading ability occurred more frequently after IVT: 6/20 (30%) versus 1/39 (3%) at d5 (p=0.005) and at d30 5/20 (25%) versus 2/37 (5%) (p=0.045). Two patients experienced serious adverse events (one angioedema and one bleeding from an abdominal aortic aneurysm) but recovered without sequelae |
| MacGrory ^101^ | 2020 | USA | Evaluate the efficacy of intravenous tissue-type plasminogen activator (IV alteplase) in the treatment of CRAO | Cohort | 112: 25 intervention, 87 control | Alteplase within 4.5 hours of onset | Standard care | Mean change in visual acuity was 1.0 ±1.11 logMAR in the intervention group which was significantly improved compared to 0.3 ±0.7 logMAR in controls. In the intervention group, 56.3% had visual acuity improvement of at least 0.3 logMAR. One patient had an asymptomatic intracerebral haemorrhage after IV alteplase treatment. Forty-four percent of alteplase-treated patients had recovery of visual acuity when treated within 4.5 hours versus 13.1% of those not treated with alteplase (p=0.003) and 11.6% of those presenting within 4 hours who did not receive alteplase (p=0.03). The updated patient-level meta-analysis of 238 patients included 67 patients treated with alteplase within 4.5 hours since time last known well with a recovery rate of 37.3%. This favourably compares with a 17.7% recovery rate in those without treatment. In linear regression, earlier treatment correlated with a higher rate of visual recovery (p=0.01) |
| Rozenberg^98^ | 2022 | Israel | Compare the visual outcome of patients treated for NA-CRAO in a medical centre that uses hyperbaric oxygen therapy as part of the standard of care to a medical centre that does not | Case comparison | 134:  121 intervention  23 standard care | Hyperbaric oxygen therapy | Standard care | Significant improvements in BCVA in the intervention group (baseline 2.89 ±0.98 to last assessment of 2.15 ±1.07 logMAR) compared to non-significant changes in the control group (baseline 3.04 ±0.82 to last assessment of 2.80 ±1.50 logMAR)​​ |
| Schonecker^99^ | 2022 | Germany | Evaluate the efficacy of iIVT and describe the prevalence of co-occurring ischemic stroke in patients with acute visual loss due to ischemia | Case comparison | 25:  9 intervention  16 standard care | rTPA | Standard care | Impact of intervention on ADL was measured by the mRS. The trial reports a significant difference in the mRS, with an improvement of 0.9 ± 0.9 in the intervention group compared to a marginal improvement of 0.1 ± 0.6 in the control group. Patients treated with IVT had a significantly better functional outcome at discharge compared to patients treated conservatively. No additional ischemic brain lesions were detected (0 of 38). Three patients had extracranial carotid artery stenosis ≥50%. Atrial fibrillation was present in four patients, three of whom already received oral anticoagulation. In the remaining 31 patients no embolic source was detected. However, the number of plaques were rated mild to moderate. Within three months, one patient developed transient visual loss while another suffered a contralateral transient ischemic attack. |
| Suzuki^102^ | 2022 | Japan | Investigate the anatomical and functional changes in patients with CRAO treated with 10 µg/day intravenous lipo-PGE1 | Cohort | 21 | IVlipo-PGE1 | N/A | Seventeen patients received lipo-PGE1 for 14 days and the other four patients for 7days. The mean time from onset to lipo-PGE1 administration was 54.7hours; range, 2–240hours. There were no adverse effects including cerebral haemorrhage recognised in the current study.  Visual acuity improved significantly from baseline (mean 2.18 ±0.60 logMAR) to follow-up at 1-month (mean 1.54 ±0.84 logMAR) and at 3 months (mean 1.53 ±0.88)**.** The BCVA values at 1month and 3 months were significantly better than that at the first visit (p=0.030 and p=0.027, respectively). |
| Raber^100^ | 2023 | Germany | Investigate the feasibility, effectiveness, and safety of IVT in the treatment of acute NA-CRAO in patients classified as functionally blind (WHO) | Case comparison | 37:  16 intervention  21 controls | rTPA | Conservative treatment like bulbar massage and pressure lowering medications | Patients were treated either conservatively or with IVT within 4.5 hours. Visual acuity was significantly improved in the intervention group (baseline visual acuity of 2.30 logMAR, IQR 2.10-2.30 to post 2.10, IQR 1.45-2.30) vs controls (baseline visual acuity of 2.30 logMAR, IQR 1.90-2.70 to post 2.30, IQR 1.60-2.30). 3 patients (19%) of the IVT group showed a favourable outcome, all control group patients remained at the level of functional blindness. No serious adverse events were observed after IVT |

ADL (activities of daily living); BCVA (Best corrected visual acuity); CRAO (Central retinal artery occlusion); IV (intravenous); IVT (intravenous thrombolysis); lipo-PGE-1 (Liposomal prostaglandin E1); mRS (modified Rankin Score); NA-CRAO (non-arteritic central retinal artery occlusion); rTPA (recombinant tissue plasminogen activator); World Health Organisation (WHO).

**Supplementary table 14: PICO 10: For adults with central vision impairment due to stroke, does compensatory, substitute or restitutive intervention, compared to no intervention, improve activities and quality of daily life?** **Intervention results.**

| **Author** | **Date** | **Country** | **Aim** | **Design** | **Sample** | **Intervention** | **Control** | **Results** |
| --- | --- | --- | --- | --- | --- | --- | --- | --- |
| Freeman & Rudge^109^ | 1987 | UK | Identify the orthoptic problems associated with stroke | Cohort | 55 (of 273 total) | Orthoptic assessment and management with glasses, advice, observation and CVI registration for reduced VA | N/A | 4 with reduced visual acuity, 12 with partial or full recovery over average 63 days (1 week to 6 months); 5 with no recovery. Remainder not retested to evaluate recovery |
| Lotery^110^ | 2000 | UK | Examine visual status of patients after stroke | Cohort | 77 | Glasses prescription within 2 weeks of admission with stroke | N/A | VA measured in 67 of 77 patients: 20 had impaired VA of 6/12 or worse but improved for 11 with updated glasses |
| Rowe^1^ | 2022 | UK | Profile visual impairment following  stroke in a large epidemiology study, taking into account the extent and type of visual impairment, associated symptoms and ocular deficits, management options and outcomes | Epidemiology | 1204 (of 1500 total) | Orthoptic assessment and management with glasses, reading aids, overlays, typoscopes and advice | N/A | VA improved on follow-up with a mean follow-up of 93.75 days (SD 102.84) after stroke; median 58 days (range 1–530). Full recovery (better than 0.2 logMAR) was recorded for 35.6% (n=126), partial recovery for 36.4% (n=129) and no recovery  noted for 25.4% (n=90). The remaining stroke survivors did not have follow-up data because of illness, discharge or death. The time duration to attain full recovery was 47.71 days (SD 55.87; median 31 days (6–308)) |

CVI (Certification of visual impairment); VA (Visual acuity).

**Supplementary table 15: PICO 11: For adults with eye movement disorders due to stroke, does compensatory, substitute or restitutive intervention, compared to no intervention, improve activities and quality of daily life? Intervention results.**

| **Author** | **Date** | **Country** | **Aim** | **Design** | **Sample** | **Intervention** | **Control** | **Results** | |
| --- | --- | --- | --- | --- | --- | --- | --- | --- | --- |
| Schaadt^114^ | 2014 | Germany | Evaluate whether impaired motor fusion and stereopsis can be treated using prismatic and dichoptic devices in 2 consecutive samples of patients with either stroke or traumatic brain injury (TBI) | Cohort | 20 | The intervention was delivered twice weekly for 60 minutes over a 6-week period. Three treatment devices were used:  1) Horizontal, convergent fusion with prisms at an amplitude they could just fuse. Increasing the amplitude when able to fuse images for at least 2 minutes without diplopia. 2) Application of a dichoptic device  3) A cheiroscope was used.  5 minutes practice per picture, performing 3 drawings in each therapy session.  Each treatment device was used for 20 minutes every session | N/A | There was a main effect of treatment for convergent motor fusion: p<0.001. Pairwise comparisons indicated a significant improvement between pre-therapy for stroke (mean 8.39, SD 4.54) and post-therapy (mean 23.9, SD 10.55). No significant difference was found between the post-therapy above and follow-up measurements (mean 22.50, SD 10.31).  There was a significant difference between pre- and post-treatment for subjective reading duration (pre-treatment 14.41 minutes (SD 13.90), post-treatment 60.55 minutes (SD 44.05)).  A main effect of treatment was found to be statistically significant (p<0.001). | |
| Schow^113^ | 2016 | Denmark | To evaluate the effect of a four-month rehabilitation program for individuals with balance problems and BVD after a stroke | Cohort | 30 | Vision training including fixation training, vergence, eye-hand co-ordination and binocularity.  The group training intervention was delivered three days per week for 2 months followed by two days per week for a further two months, with home exercises instructed on every day not participating in group training.  Balance training involved individualized sensory integration, vestibular and proprioceptive exercises.  Visual therapy included training of fixation, tracking, vergence, eye-hand coordination and binocularity | N/A | All measures: stereoacuity, King Devick, binocular fusion, positive relative vergence improved significantly from baseline to the 6-month follow-up visit except from distance binocular fusion and near negative relative vergence.  Functional recovery as measured by the GOSE, at baseline 44.4% had severe disability, 55.2% had moderate disability, with only one person (3.4%) having a good recovery). At the 6-month follow-up visit 8% continue to have severe disability, 68% moderate and 24% had improved to a good functional outcome. The change in the GOSE mean was statistically significant (p<0.01).  The overall mean EQ-5D-3L quality of life score at baseline: 0.643 (SD 0.14) and 6-month follow-up was 0.743 (SD 0.199), indicating a statistically significant change of 0.1 (p=0.01). The mean EQ-5D visual analogue score at baseline was 46.03 (SD 19.72) and at 6-month follow-up improved to 62.36 (SD 22.11), a statistically significant change of 14.56 (p<0.001). | |
| Johansson^112^ | 2021 | Sweden | Investigate the effects of vision therapy as part of neuro rehabilitation after acquired brain injury | Case control | 89:  48 intervention  41 controls | Vision therapy for ocular motility dysfunction. The vision therapy group received therapy targeted to their type of ocular motor deficit. Therapy was undertaken 3 times per week (total therapy time of 60 minutes per week).  The vision therapy program began with eye teaming exercises followed by gaze-related exercises. | Standard care | Within the intervention group, the near point of convergence improved from median 20cm to 12cm (p=0.02). The improvement seen for the control group was not statistically significant.  Convergence facility improved for both groups. Only the intervention group had a statistically significant change (p=0.03). Vergence reserves increased statistically significantly or distance viewing in both intervention (p<0.01) and the control group (p=0.04). Both groups improved the level of vergence reserves for near viewing to within or equal to the target, on discharge. This change was statistically significant for the intervention group (p<0.01). The intervention group also saw a statically significant reduction in symptoms (p<0.01), which was not found in the control group | |
| Batool^111^ | 2022 | Pakistan | Determine the effects of visual scanning exercises in addition to task specific approach on balance and activities of daily living in stroke patients with eye movement disorders | RCT | 64:  32 intervention  32 control | Visual scanning exercises with a task-specific approach. Participants performed eye movements (upward, downward, towards midline, laterally and diagonal movement of eyes) which were impaired in each functional position i.e. in supine lying, side lying to sitting, in sitting, during sit to stand, in standing and during walking. Intervention was delivered for 45 minutes per day, 6-days per week over a 4-week period | Task specific approach with placebo eye exercises.  Participants performed different task specific exercises e.g. feet balanced on mat/gym ball in lying position while doing bridging.  The task specific approach was delivered for 30 minutes with placebo exercises delivered for 15 minutes.  The placebo eye exercises involved random eye movements responding to a torch light displayed by the therapist.  The control was delivered for a total of 45 minutes, 6- days per week, over a 4-week period | Berg balance scale revealed significant improvement for the intervention group and control group (p=0.0001) the mean differences were 5.59 ± 0.71 and 1.44 ± 0.34, respectively.  Barthel index score also revealed significant improvement for the intervention and control groups (p=0.0001) the mean differences were 14.39 ± 5.22 and 5.94 ± 2.98, respectively.  The after intervention intergroup comparison of intervention vs control groups revealed a significant different in the Berg Balance Scale of 16.34 ± 2.88 vs 12.63 ± 2.52 (p=0.0001) and Barthel Index of 32.66 ± 12.69 intervention vs 26.25 ± 10.70 controls(p=0.033) |  |
| Rowe^1^ | 2022 | UK | Profile visual impairment following  stroke in a large epidemiology study, taking into account the  extent and type of visual impairment, associated symptoms and  ocular deficits, management options and outcomes | Epidemiology | 1204 (of 1500 total) | Standard orthoptic assessment and routine management options offered. | N/A | Full or partial improvement of ocular motility abnormalities was recorded for 328 (69.4%) stroke survivors. Full recovery was reported for 15% of ocular misalignment, 35.7% of ocular movement disorders and 26% of binocular vision defects. Full recovery was attained in a mean of 88.58 days (SD 94.25) for ocular misalignment, 59.66 days (SD 64.71) for ocular movement disorders and 58.02 days (SD 66.58) for binocular vision defects.  Various management strategies were offered and were aimed at the stroke survivor’s main visual symptom. The routine management options offered included prisms/occlusion (10.4%), scanning training (5.9%), orthoptic exercises (1.3%), advice and compensatory strategies (24.1%) and referral to other ophthalmic clinics (50.7%). |  |

BVD (binocular vision dysfunction); GOSE (Glasgow functions Outcome Scale – Extended); RCT (randomised controlled trial).

**Supplementary table 16: PICO 12: For adults with visual neglect due to stroke, does compensatory, substitute or restitutive intervention, compared to no intervention, improve activities and quality of daily life? Intervention results.**

| **Author** | **Date** | **Country** | **Aim** | **Design** | **Sample** | **Intervention** | **Control** | **Results** |
| --- | --- | --- | --- | --- | --- | --- | --- | --- |
| Chen^157^ | 2014 | USA | Used lesion localization to examine the ability of frontal lesions to predict functional improvement after PAT and used VLBM to identify the intact regions that may mediate the PAT effect on functional improvement | Cohort | 21 | 7 assessment sessions and 10 sessions of the PAT. Catherine Bergego scale used to assess spatial neglect in functional activities. The outcome was quantified in 10 items: limb awareness, personal belongings, dressing, grooming, gaze orientation, auditory attention, navigation, collisions, eating, and cleaning after meal. Participants received PAT for two weeks, five sessions per week, one session per day. The prisms displaced the visual field horizontally rightward by 12.4 degrees of visual angle, and the goggle provided an opaque frame to block distraction from peripheral visual stimuli | N/A | Functional activities improved after two weeks of PAT and continued improving for four weeks. Neglect patients with lesions involving the frontal cortex (n=13) experienced significantly better functional improvement than did those without frontal lesions (n=8). VLBM revealed that in comparison to the group of patients without frontal lesions, the frontal-lesioned neglect patients had intact regions in the medial temporal areas, the superior temporal areas, and the inferior longitudinal fasciculus. The medial cortical and subcortical areas in the temporal lobe were especially distinguished in the “frontal lesion” group. The findings suggest that the integrity of medial temporal structures may play an important role in supporting functional improvement after PAT.  In the visual-proprioceptive pointing task, the “frontal lesion” group's leftward prism after effect reached significance, p=0.050, but the “no frontal lesion” group's did not, p=0.306. In the proprioceptive pointing task, the “frontal lesion” group's leftward prism after effect approached significance, p=0.051, but the “no-frontal lesion” group's did not, p=0.306 |
| Goedert^158^ | 2014 | USA | Test the effect of PAT - 3 groups: one with Where only, one with Aiming only, the third with Where + Aiming. test the hypothesis that classifying patients by their profile of Where-versus-Aiming spatial deficit would predict response to prism adaptation and specifically that patients with Aiming bias would have better recovery than those with isolated Where bias | Cohort | 24 | Classified the spatial errors of 24 subacute right stroke survivors with left spatial neglect as (1) isolated Where bias, (2) isolated Aiming bias, or (3) both.  received PAT once daily for 10 days.  Each PAT session  lasted 15 to 20 minutes | N/A | The Aiming-only and Aiming + Where participants differed significantly from the Where-only participants (z = −2.60; p=0.009) but not from each other (z = −1.02; p=0.306). Aiming-only participants had the steepest linear recovery (β = −0.56; b = −0.41; standard error [SE] = 0.17; confidence interval [CI] = −0.73, −0.08; z = −2.44; p=0.015), whereas Where-only participants had a slope that did not differ from zero (β = −0.11; b = −0.08; SE = 0.17; CI = −0.42, 0.24; z = −0.52; p=0.516). Participants with Aiming + Where bias had an intermediate slope (β = −0.41; b = −0.30; SE = 0.16; CI = −0.61, 0.01; z = −1.89; p=0.058). These results support the hypothesis: participants with a rightward Aiming bias showed greater improvement with PAT |
| Grattan^159^ | 2014 | USA | Establish feasibility and tolerability of a repetitive task practice program for USN after stroke. Examine improvements in symptoms associated with USN syndrome after treatment | Cohort | 20 | 1 hour/day, 3 days/week for 6 weeks. Standardized repetitive task practice program to individuals with chronic stroke. The repetitive task practice program involved high doses of repetitive training for the impaired arm in the context of functional tasks, and were administered 1 hour/day, 3 days/week for 6 weeks. Tasks were switched, downgraded, and upgraded throughout the course of the 18 intervention sessions. Participants completed both unilateral and bilateral tasks during the sessions | N/A | Participants continued to have mild to moderate neglect but experienced a small but significant improvement on the Catherine Bergego Scale (p<0.01). No changes were seen in self-assessment Catherine Bergego Scale. Participants continue to report difficulties to complete activities of daily living; the small reduction in disability was not statistically significant |
| Kerkhoff^119^ | 2014 | Germany | Compare the effects of smooth pursuit eye movement training and VST in post acute stroke at 1 month with left neglect | RCT | 24 | VST. Patients were trained to scan systematically from left to right and from top to bottom, naming all objects, or counting certain stimuli.  20 treatment sessions (30 minutes each) in  total (1 session daily, from Monday to Friday) over a period  of 1 month | Smooth pursuit eye movement training. The patient was encouraged to conduct smooth pursuit eye movements, repeatedly following the stimulus pattern from right to left, without head movements | Significantly greater improvements were obtained after smooth pursuit eye movement training versus VST and there were continued improvements selectively in the smooth pursuit eye movement training group 2 weeks later. Conclusions from this study were that smooth pursuit eye movement training accelerates recovery from functional neglect and reduces unawareness significantly.  After both treatments, the Barthel Index and Help Index improved but without a statistical differential treatment effect (as shown by the nonsignificant interaction). |
| Pandian^120^ | 2014 | India | Explore the effectiveness of MT in the treatment of unilateral neglect in stroke patients. | RCT | 48 | Patients received treatment for 1–2 hours a day 5 days a week for 4 weeks.  MT; Patients were instructed to perform flexion and extension movements of the nonparetic wrist and fingers while looking into the mirror | Patients received treatment for 1–2 hours a day 5 days a week for 4 weeks. The control group carried out similar exercises for the same time period but they used the non-reflecting side of the mirror. The paretic hand was hidden from their sight | Based on the FIM, the patients in the treatment group were more likely to be independent during follow-up. Good outcome was seen in more patients in the treatment group at 6 months (mRS).  Improvement in scores on the star cancellation test over 6 months was greater in the MT group (mean difference 23, 95% confidence interval [CI] 19–28; p<0.0001). Similarly, improvement in the MT group was observed in the scores on the picture identification task (mean difference 3.2, 95% CI 2.4–4.0; p<0.0001) and line bisection test (mean difference 8.6, 95% CI 2.7–14.6; p=0.006) |
| Van Wyk^122^ | 2014 | South Africa | To determine the effect of saccadic eye movement training with visual scanning exercises integrated with task-specific activities on USN poststroke | RCT | 24 | Saccadic eye movement training + task-specific activities. 45 minute sessions, 5 days per week for 4 consecutive weeks | Task-specific activities. 45 minute sessions, 5 days per week for 4 consecutive weeks | A statistically significant difference (p=0.04) was noted when the functional improvement (reading performance measured with the German version of the Rivermead Behavioural Memory test) between the two groups after the intervention period was compared with the mixed-model rank ANCOVA analysis |
| Machner^121^ | 2014 | Germany | To assess if HEP repetitive OKS in acute stroke patients with neglect induces greater remission of neglect than in the spontaneous course | RCT | 21 | Patients in the treatment group received HEP OKS in addition to the usual stroke care (physio-, speech, and occupational therapy). HEP was applied by spectacle frames containing noncorrective lenses of which the right half was patched with dark non translucent tape. Participants were instructed to wear the glasses all-day for 7 days and only to remove them for the OKS treatment sessions | Usual stroke care (physio-, speech, and occupational therapy) | For Catherine Bergego Scale score, there was no significant main effect of group or the interaction group*session, but only of session (F(2,18)=45.2, p<0.001). Within both groups, the Catherine Bergego Scale score decreased equally between post-treatment and follow-up session (treatment: d=−9.0±1.9, p<0.01; control: d=−8.2±1.8, p<0.01) |
| Yang^125^ | 2015 | China | Compare the effects of rTMS combined with sensory cueing, rTMS alone, and  conventional rehabilitation on unilateral neglect, hemiplegic arm functions and performance of  activities of daily living | RCT | 60 | Intervention 1; rTMS. Inhibitory rTMS at 1Hz was applied over P5 of the contralesional hemisphere at an intensity of 90% of the individual’s resting motor threshold. The stimulus was delivered at 900 pulses/session, with 1 session daily for 2 weeks.  In the sensory cueing protocol patients were asked to wear the device on their left wrist for three hours a day, five times a week, over the two weeks | Conventional therapy - that also the other two groups received. 2-week conventional rehabilitation treatment consisted of 30 sessions of 45 minutes each, 2 sessions for physiotherapy sessions, and 1 occupational therapy session daily, for 5 days per week | Both rTMS combined with sensory cueing (99.6±33.0) and rTMS alone (88.2±28.7) significantly reduced unilateral neglect than conventional rehabilitation (72.7±33.1) when measured using the conventional subtests of the Behavioural Inattention Test, but the combination was better than rTMS alone. Hemiplegic arm functions and activities of daily living improved in all patients across the three groups but no significant differences were found between the groups |
| Aparicio-Lopez^128^ | 2016 | Spain | Analyse whether the combined administration of computerized cognitive rehabilitation with right hemifield eye-patching in patients with left spatial neglect following a right hemisphere stroke is more effective than computerized cognitive rehabilitation applied in isolation | RCT | 28  13 intervention  15 alternative | Average of 15 one-hour sessions of computerized cognitive rehabilitation using the Guttmann, NeuroPersonalTrainer telerehabilitation platform  combined administration of computerized cognitive rehabilitation with right hemifield eye-patching | Average of 15 one-hour sessions of computerized cognitive rehabilitation using the Guttmann, NeuroPersonalTrainer telerehabilitation platform  computerized cognitive rehabilitation applied in isolation | After the intervention, the ST group showed statistical significance in Bell Cancellation Test (p=0.001), Figure Copying of Ogden (p=0.016), Line Bisection (right deviation) (p=0.002), Line Bisection (lines omitted) (p=0.017), Baking Tray Task (left) (p=0.026), and Baking Tray Task (right) (p=0.026), whereas the control group showed statistical significance in Bell Cancellation Test (p=0.003), Line Bisection (right deviation) (p=0.019), Baking Tray Task (left) (p=0.042), and Baking Tray Task (right) (p=0.042). No differences in either group were observed in the Catherine Bergego Scale administered pre- and post-intervention |
| Choi^124^ | 2016 | Korea | Investigate the effectiveness of an upper limb rehabilitation robot therapy on hemispatial neglect in stroke patients | RCT | 38 | 30 minutes per day, 5 days a week for 3 weeks.  Neuro-X system was used for the robot treatment of hemispatial neglect. During the treatment, each patient sat on the right side of the robot so that the monitor was located to the left side of the patient. In this position, the patient could focus continuously on the left side | 30 minutes per day, 5 days a week for 3 weeks.  conventional neglect treatment, such as visual scanning training and range of motion exercises, administered by occupational therapists | Both groups showed significant improvements in the MVPT), line bisection test, star cancellation test, Albert’s test, Catherine Bergego scale, Mini-Mental State Examination and Korean version of MBI. The changes in all measurements showed no significant differences between the two groups. During the robot treatment, there were no treatment side effects, such as dizziness and upper extremity pain. |
| Kim^126^ | 2016 | Korea | Examine the effects of tDCS on visual perception and performance of activities of daily living in patients with stroke | RCT | 30 | Traditional occupational therapy treatment and tDCS. A current of 1 mA was applied for 20 min | Traditional occupational therapy and sham tDCS: electrodes were applied to the same location for the same length of time as above, but the current was discontinued after 30 seconds | Both groups improved in visual perception function and in performance of activities of daily living (FIM). Although there was no significant difference between the two groups, the experimental group exhibited higher scores.  For both groups, comparison of the MVPT results before and after the intervention revealed a statistically significant difference (p<0.05). Results of the MVPT showed that the experimental group demonstrated a significant improvement of approximately six points, from 21.1 ± 3.6 points to 26.8 ± 3.1 points on average, after the intervention. The control group also improved in visual perception after the intervention, increasing from 21.0 ± 3.9 points to 23.9 ± 3.8 points, which was also statistically significant (p=0.05). The total FIM score showed that the experimental group increased by 13 points after the intervention, from 66.8 ± 9.5 points to 79.57 ± 11.3 points. The FIM score of the control group increased by three points, from 65.4 ± 11.4 points to 68.3 ± 18.4 points. Both increases were statistically significant (p<0.05) |
| Yi^127^ | 2016 | Korea | Examine whether tDCS applied over the posterior parietal cortex improves visuospatial attention in stroke patients with left visuospatial neglect | RCT | 30 | Intervention 1; anodal tDCS with a constant current of 2 mA was delivered for 30 minutes, 5 times per week for 3 weeks.  Intervention 2;  catodal tDCS with a constant current of 2 mA was delivered for 30 minutes, 5 times per week for 3 weeks | Sham tDCS with a constant current of 2 mA was delivered for 30 minutes, 5 times per week for 3 weeks | From pre- to post-treatment, there was an improvement in the MVPT, line bisection test, star cancellation test, Catherine Bergego Scale, Korean version of MBI), and Functional Ambulation Classification in all 3 groups. Improvements in the MVPT, star cancellation, and line bisection were greater in the anodal and cathodal groups than in the sham group. However, improvements in the outcomes were not significantly different between the three groups, although there was a tendency for improved Catherine Bergego Scale or Korean version of MBI scores in the anodal and cathodal groups, as compared with the sham group |
| Aparicio-Lopez^128^ | 2017 | Spain | Assess how the time between stroke and the start of treatment impacts (or can impact) patient improvement, and to analyse patient response to two treatments (single treatment: computerised cognitive stimulation vs. combination treatment: computerised cognitive stimulation with right hemifield eye-patching) | RCT | 31  13 intervention  18 single therapy | Average of 15 one-hour sessions of computerized cognitive rehabilitation using the Guttmann, NeuroPersonalTrainer telerehabilitation platform  combined administration of computerized cognitive rehabilitation with right hemifield eye-patching | Average of 15 one-hour sessions of computerized cognitive rehabilitation using the Guttmann, NeuroPersonalTrainer telerehabilitation platform  computerized cognitive rehabilitation applied in isolation | No statistically significant differences between groups for Catherine Bergego Scale whether self-administered or hetero-administered.  After the intervention, the combined treatment group showed statistical significance in Bell Cancellation Test (p=0.046 for ≤ 12 weeks, 0.028 for > 12 weeks), Line Bisection (right deviation) (p=0.028 for ≤ 12 weeks, 0.176 for > 12 weeks) and sentence reading (p=0.109 for ≤ 12 weeks, 0.042 for > 12 weeks). No statistically significant differences were observed for Catherine Bergego Scale self-administered (p=0.207 for ≤ 12 weeks, 0.236 for > 12 weeks) or hetero-administered (p=0.173 for ≤ 12 weeks, 0.499 for > 12 weeks). After the intervention, the single treatment group showed statistical significance in Bell Cancellation Test (p=0.009 for ≤ 12 weeks, 0.041 for > 12 weeks), Figure Copying of Ogden (p=0.025 for ≤ 12 weeks, 0.414 for > 12 weeks), Line Bisection (right deviation) (p=0.016 for ≤ 12 weeks, 0.018 for > 12 weeks), Line Bisection (lines omitted) (p=0.041 for ≤ 12 weeks, 0.109 for > 12 weeks), Baking Tray Task (left) (p=0.088 for ≤ 12 weeks, 0.042 for > 12 weeks) and Baking Tray Task (right) (p=0.136 for ≤ 12 weeks, 0.042 for > 12 weeks). No statistically significant differences were observed for Catherine Bergego Scale self-administered (p=0.722 for ≤ 12 weeks, 0.753 for > 12 weeks) or hetero-administered (p=0.066 for ≤ 12 weeks, 0.917 for > 12 weeks) |
| Hreha^160^ | 2017 | USA | Determine the feasibility and effectiveness of using PAT to improve spatial and motor functions in stroke survivors with multiple strokes | Cohort | 26 | 60 times or until 20 min had elapsed, 1 session per day, 10 sessions in total of PAT. The PAT protocol first required the participant to wear wedged 20 dioptre prism lenses that were fixed into a goggle. The participant moved their right arm, starting at their body centre to a specified target (line or circle on a piece of paper) and then marks with a pen, the centre position of that line or circle. These targets were placed on the table either at the middle, right, or left of the participant | comparison group received the standard occupational therapy. Standard therapy included “neglect therapies” (not including PAT) that the treating therapist deemed appropriate | For the line bisection test, out of the four two-way com- parisons using the U-test, only one comparison reached  The treatment group improved significantly from baseline to post treatment, p=0.002. Likewise, the star cancellation data only one comparison resulted in a p value reaching significance. The treatment group performed better post treatment, comparing to their baseline performance (p=0.001). For the Catherine Bergego Scale via the KF-NAP data, a 2 × 2 repeated measures ANOVA yielded a significant main effect of Assessment time, p<0.01.  Unfortunately, a significant treatment group results did not hold true for the Catherine Bergego Scale via KF-NAP, however both groups did show functional improvements over time. Regarding motor function, on the Motor FIM, both the treatment and the comparison groups’ motor function improved after the intervention. However, the two groups did not differ with their improvement on the Motor FIM score |
| Ten Brink^129^ | 2017 | The Netherlands | Assess whether PAT in the subacute phase ameliorates neglect in situations of varying complexity | RCT | 69 | Once daily each working day for 2 weeks  Patients wore a pair of goggles fitted with wide-field point-to-point prismatic lenses, inducing an ipsilesional optical shift of 10° (PAT). Exposure consisted of 100 fast pointing movements to 3 stimuli (red, yellow, blue) presented on a horizontal axis at a distance of 65 cm. The left and right stimuli were located 10° away from the body midline. The investigator indicated which stimulus was the target | 30-60 minutes each working day  Usual care differed per patient and contained 4 to 6 therapies (e.g., physical, occupational, speech.  Patients wore goggles with plain lenses | Intervention: Catherine Bergego Scale week 4: mean 9.02, SD 5.54.  Shape cancellation week 4: mean 2.50, SD 3.93. MAC week 4: mean 3.27, SD 3.07. CBS week 6: mean 9.46, SD 5.46. SC week 14: mean 1.14, SD 1.98.  Control: Catherine Bergego Scale week 4: mean 10.88, SD 7.25. SC week 4: mean 2.85, SD 5.87. MAC week 4: mean 4.53, SD 3.46. MAC week 14: mean 2.51, SD 2.28. CBS week 6: mean 11.04, SD 7.94. SC week 14: mean 2.16, SD 4.54. MAC week 14: mean 3.03, SD 2.67.  There were significant time-dependent improvements in performance as measured with the Catherine Bergego Scale, mobility assessment, and star cancellation (all F > 15.57; p<0.001). There was no significant difference in magnitude of improvement between groups on the CBS, MAC, and SC (all F < 2.54; p> 0.113] |
| Kim^130^ | 2018 | Korea | Effect of combined therapy of robot and low-frequency repetitive transcranial magnetic stimulation on hemispatial neglect in stroke patients | RCT | 30 | Intervention 1; rTMS therapy with a coil stimulator shaped like a figure-8 at diameter of 70 mm using MagPro. Sessions included 900 stimuli applied over the contralesional posterior parietal cortex at an intensity of 95% motor thresholds and a frequency of 0.9 Hz. 20 minutes per day, 5 days a week for 2 weeks (10 times total).  Intervention 2; rehabilitation robot (Neuro-X).  Robot therapy program was conducted through games that induced passive and active assistive range of motion of the wrist, elbow, and shoulder joints. These games consisted of two isometric exercises and two range of motion exercises. 20 minutes per day, 5 days a week for 2 weeks (10 times total) | Combination: both rTMS therapy and robot therapy. To maximize the therapeutic effect in the combined group, the rTMS therapy was performed first and then the robot therapy was performed within 2 hours after the first therapy was completed which was the aftereffect period. treatment for 40 minutes per day, 5 days a week for 2 weeks (10 times total) | Two weeks after the therapy, all groups showed significant improvement in (motor free visual perception test) MVPT-3, line bisection test, star cancellation test, Catherine Bergego Scale, MMSE, and K-MBI (Korean modified Barthel index). However, changes in measurements showed no significant differences among groups |
| Kutlay^131^ | 2018 | Turkey | Investigate the effects of kinaesthetic ability training with the Kinesthetic Ability Trainer on unilateral neglect and functional outcomes in stroke patients | RCT | 64 | 5 times per week, with a session-duration of 20–30 min. Using the SportKAT 2000 for a 4-week period | 5 times per week, with a session-duration of 20–30 min. For 4 weeks.  Conventional programme is tailored to the patient’s needs and consists of physical and occupational therapy | Both groups showed significant improvements in all subscales of the BIT(p<0.001) and the FIM motor scale (p<0.001) after therapy. The FIM scores improved significantly in both therapy groups except the FIM cognitive score in the Kinesthetic Ability Trainer group. When percentage change in the FIM scores from baseline to after therapy were compared, no significant difference was detected between the 2 groups |
| Luaute^132^ | 2018 | France | Test whether combining PAT and methylphenidate could enhance the recovery of neglect patients at a functional level | RCT | 24 | PAT + methylphenidate  five sessions of PAT between T0 and T1 + 20 mg of methylphenidate per day, for 5 days | PAT+ placebo  five sessions of PAT between T0 and T1 + 20 mg of placebo per day, for 5 days | The main result was a long-term functional improvement (on the FIM and on the Catherine Bergego Scale) induced by methylphenidate combined with PAT. The interaction between groups and time showed a significant effect of treatment over time only for two variables: the FIM and Catherine Bergego Scale scores. A greater improvement was observed across sessions for both variables in the methylphenidate group as compared with the placebo group |
| Turgut^133^ | 2018 | Germany | Investigate the effect of adaptive cueing during a reading task as a possible treatment for neglect by including (1) a task relevant for the patient’s daily life, (2) a fading out procedure to stimulate independent orientation to the left by self-cueing, and (3) a clear definition of neglect severity for the adaptive treatment protocol | RCT  Cross  Over | 26 | The daily treatment for the intervention group consisted of a reading task with adaptive cueing for at least 20 minutes and a maximum of 45 minutes. Cueing consisted of visual highlights (exogenous) and verbal instructions, which require intrinsic actions (endogenous). Patients were categorized based on their reading performance and the independent implementation of the cues | After 15 daily sessions in 3 weeks, the evaluation of the first condition was performed immediately after the last therapy session (T3). Again 15 daily sessions in 3 weeks were performed and ended with the evaluation of the second condition | Significant improvements were shown after intervention on scores for reading (word and text reading), ADL (Catherine Bergego Scale), line bisection, and clock drawing task.  Catherine Bergego Scale: pre intervention 17.0 (±1.6), post 7.5 (±1.6) (±2.1), clock drawing test pre 1.5 (±0.1), post 1.0 (±0.1), line bisection (cm) pre 4.6 (3.6), post 3.1 (2.9), apples cancellation task (omission) 38.1 (11.6), post 26.7 (15.0) |
| Choi^134^ | 2019 | Korea | Examine the combined effects of PAT plus FES on stroke patients with unilateral neglect, and suggest a new intervention method for acute-phase stroke patients | RCT | 30 | Group A received 30 minutes of conventional occupational therapy, followed by FES application on the upper limb on the affected side and PAT for 20 minutes, for a total of 50 minutes.  Group B received 30 minutes of conventional occupational therapy, followed by PAT on the upper limb on the affected side for 20 minutes, for a total of 50 minutes | Group C received 30 minutes of conventional occupational therapy, followed by FES application for 20 minutes, for a total of 50 minutes | Group a; Mean MVPT: -8.249 9 p<0.001, Albert test mean pre 15.3 ± 3.49 post 5.0 ± 2.21 t 13.285 df 9 p<0.001, Catherine Bergego Scale comparison pre 20.9 ± 2.99, post 10.8 ± 2.78, t 17.237, df 9, p value < 0.001.  All three groups showed unilateral neglect reduction after the intervention, but PAT plus FES (complex intervention method) was more effective than PAT or FES alone [effect size: MVPT (0.80), Albert test (0.98), Catherine Bergego Scale (0.92)] |
| Karner^135^ | 2019 | Germany | Evaluate the effects of an intervention using the robot device PARO (an interactive robotic toy with the appearance of a baby seal, which can move, produce sounds, and react to speech and touch) on visuospatial hemineglect and activities of daily living, and its acceptance during stroke rehabilitation | RCT | 39 | For 2 weeks: 30 minutes/day; three days per week, 6 sessions /patient.  PARO was placed on the neglected side so that it  was possible for the patient to see and grasp it. The  task for the patient was focussing the attention on  the robot | For 2 weeks: 30 minutes/day; three days per week, 6 sessions /patient.  Patients were read aloud from a book for the same time as the PARO intervention | Both groups showed an improvement in the SINGER subcategories of self-care, mobility, and communication and cognitive ability over the study period. The differences between the groups in the subcategories of self-care, mobility, and communication were not significant. In the subcategory of cognitive abilities, we found a significant improvement in the PARO group compared to the control group at T1 and T2 |
| Nyffeler^136^ | 2019 | Switzerland | Assess the characteristics and determinants of the effects of inhibitory non-invasive brain stimulation in neglect, identifying which patients would respond to this therapeutic approach and which would not | RCT | 60 | Intervention 1; 8 trains of cTBS, over the left posterior parietal cortex. cTBS: 801 pulses, delivered in a continuous train of 267 bursts. The patients were asked to close their eyes during stimulation application. cTBS was delivered at  100% of the patients’ individual resting motor threshold. Intervention 2; 16 trains of cTBS, over the left posterior parietal cortex. The patients were asked to close their eyes during stimulation application. cTBS was delivered at 100% of the patients’ individual resting motor threshold. | Sham;  cTBS protocol  as for intervention, except for the use of a sham coil.  Controls; stroke survivors without neglect having standard care therapy. | On a group level, both cTBS protocols (i.e. eight and 16 trains) significantly reduced neglect severity in both the Catherine Bergego Scale and the neuropsychological tests, at discharge and 3 months later. Furthermore, cTBS significantly improved general functional outcome.  On an individual level, hierarchical cluster and voxel-based lesion-symptom mapping analyses revealed that the variability in the responses to cTBS is determined by the integrity of interhemispheric connections within the corpus callosum, in particular parieto-parietal connections. In cTBS responders, in whom neglect and general functional outcome were significantly improved, the corpus callosum was intact, whereas this was not the case in cTBS non-responders. The recovery of neglect and of the activities of daily living was accelerated only in cTBS responders. Furthermore, the level of activities of daily living recovery of these neglect patients was brought close to the one of right-hemispheric control patients without neglect. |
| Rossit^137^ | 2019 | UK | Compare the immediate and long-term effects of visuomotor feedback training vs. a control training when delivered in a home-based setting | RCT | 20 | The treatment involved repeated grasping and lifting of rods, using the non-paretic (usually right) limb. The intervention group was asked to grasp the centre of the rod until it balanced. The treatment was delivered in two consecutive phases: experimenter-led and self-led. Run for two consecutive times of approximately 30 minutes over two days. The patient independently repeated the training for 10 days. Each self-led session consisted of 72-rod lifts (8 repetitions) | Grasping and lifting of rods, in an unbalanced manner as compared to the treatment group. The control was asked to grasp the rod at one side.  10x 30-minute sessions in total, 5 times a week, for 2 weeks | Visuomotor feedback training also had an effect in the activity of daily living at four-months post-training: the visuomotor feedback training group marginally improved in activities of daily living whilst the control group deteriorated significantly.  Significantly greater short and longterm improvements were obtained after visuomotor feedback training when compared to control training in line bisection, BIT and spatial bias in cancellation. Visuomotor feedback training also produced improvements on activities of daily living |
| Wen^138^ | 2019 | China | Observe the influence of rehabilitation intervention of adjuvant horticultural therapy on unilateral spatial neglect in the stroke | RCT | 46 | Sensory stimuli and exercise training: cold stimulation (5~100℃), thermal stimulation (50~55℃), rubbing patients’ limbs and the weight training were all operated under the patients’ watch. Vision: A conscious scanning and neglect of limbs was trained. Visual training: Patients were trained to scan and track the target objects in view, making their heads turn to the neglected side | Treated through the Bobath operation combined with electroacupuncture, massage and physical therapy etc. Meanwhile, the patients were also guided to do the ADL capability training | Intergroup differences of Fugl-Meyer assessment, modified Barthel Index and Hamilton Rating Scale for Depression scores were not significant (p>0.05) before therapy, but the intergroup differences became significant after therapy (p<0.01). FIM pre. Shenckenberg test mean +/-s 2.98 +/-0.23. Albert test 3.24 +/-0.54. Clock drawing 2.09 +/-0.12. Copy drawing 2.45 +/-0.23. MBI pre. mean +/-s 30.88 +/- 3.62 |
| Iwanski^139^ | 2020 | Poland | Investigate the therapeutic effect of 1 Hz rTMS applied over the left angular gyrus combined with VST in patients with left visual spatial neglect in the subacute stroke phase | RCT | 28 | Daily 5/days a week, 30-min rTMS sessions, followed by 45-min visuospatial training, for 3 weeks.  experimental (fifteen sessions of rTMS  consisted of 1800 magnetic pulses delivered to the left angular gyrus with a neuronavigation control) | Daily 5/days a week, 30-minrTMS sessions, followed by 45-min visuospatial training, for 3 weeks.  control group  (fifteen sessions of sham stimulation), followed by VST | After the intervention both groups improved significantly (p=0.001) in all outcomes. For measures including FIM/FAM/VSS, almost all showed large effect size (r > 0.5). However, the rate of improvement was similar in both groups (rTMS vs sham) |
| Van Vleet^140^ | 2020 | USA | Examine the effectiveness of a digital health intervention targeting the intrinsic regulation of goal-directed alertness in patients with chronic hemispatial neglect | RCT | 31  15 intervention  16 controls | 39 sessions of cognitive training, intended to be delivered in 30-minute sessions over 12 weeks.  Plus 3-month no-training/no-contact follow-up period. The experimental cognitive training program was a commercially available cognitive training exercise (Freeze Frame). The exercise targeted sustained goal-directed attention-to response and inhibitory control (executive function) | The active control program was designed to provide an experience that was matched to the experimental treatment program in intensity and duration, while plausibly engaging cognitive systems to maintain the patient blind. Previously vetted off-the-shelf computer games were selected and were delivered with a schedule identical to the experimental treatment | For the primary outcome measure, the experimental group (ET) showed a significant advantage (228.57 milliseconds less rightward bias) over the control group (AC) (288.33 milliseconds more rightward bias) at the post- training visit (p = 0.010, Cohen’s d = 0.96) and demonstrated a positive trend at the follow-up visit (p = 0.16, Cohen’s d = 0.54; ET = 153.92 milliseconds less rightward bias and AC = 148.22 milliseconds more rightward bias). On a within-group basis, improvement in the ET group was 1.8 times larger than that of the AC group at post-training, and 2.0 times larger at follow-up.  Regarding the secondary measures, the ET group showed a significant advantage over the AC group (p = 0.027, Cohen’s d = 0.24) in the functional composite measure ( Catherine Bergego Scale; Barthel index) when comparing the pre- versus post-training visit (3.13 points of improvement in the Barthel and 1.06 points improvement in the Catherine Bergego Scale for ET, versus no change in the Barthel and 0.14 points worsening in the Catherine Bergego Scale for the AC group). There was no between-group difference in the functional abilities composite at the follow-up visit (p = 0.65). There was no significant between-group difference in the quality-of-life measures |
| Zigiotto^141^ | 2020 | Italy | Assess the effects of an intensive audio-visual multisensory stimulation on unilateral spatial neglect, and compare them with those of PAT | RCT | 20 | Multisensory stimulation  2x 20 minute sessions per day, 5 days a week, for 2 weeks.  Consisted in a bimodal audio-visual stimulation of the visual field. During the training, patients sat in a low illuminated room, at 100 cm from the centre of the apparatus, and were asked to look at the fixation point. Bimodal audio-visual stimuli were presented, which consisted in a visual target coupled to a sound, both lasting 100 ms | Prism adaptation  2x 20 minute sessions per day, 5 days a week, for 2 weeks.  Patients sat at a table in front of the therapist, wearing prismatic goggles inducing a 10° rightward shift of the visual field. Patients were asked to perform daily life activities (four per session) while wearing the prisms | Results showed that multisensory stimulation brought about an amelioration of USN deficits overall comparable to that induced by PAT; personal neglect was improved only by multisensory stimulation, not by PAT. The multisensory stimulation treatment had positive effects also on USN manifestations in daily living, as judged by occupational therapists (i.e., observed- Catherine Bergego Scale). However, such functional changes were not acknowledged by patients, as indexed by the self-administered form of the Catherine Bergego Scale, a result that may reflect the persistence of anosognosia for USN. The anosognosia index, although not statistically significant, showed a positive trend, indicating that PAT may also influence awareness of USN at least in daily living, a result in line with previous evidence |
| Chen^142^ | 2021 | China | Investigate the effects of RAT on unilateral spatial neglect, arm motor function, activities of daily living, and social participation after stroke | RCT | 20  10 intervention  10 controls | 5 days a week for 4 weeks. 45 min daily: 15-min passive mode and 30-min assist-as-need mode.  RAT for remediating patients' neglect of contralateral space and affected upper extremity supervised by a therapist | 45 mins at 5 times per week.  general cognitive and occupational rehabilitation dedicated for USN, consisting of VSTy, passive range of movement of upper extremity and perceptual retraining integrated with task-specific activities | Intervention: BIT-C 109.70 ± 28.28, Difference 23.40 ± 7.85. Catherine Bergego Scale 7.10 ± 4.33, Difference −5.40 ± 1.65. FMA-UE 37.70 ± 11.11, Difference 13.60 ± 4.70. MBI 74.50 ± 14.73, Difference 28.90 ± 14.26. WHODAS-2.0 98.60 ± 8.70, Difference −23.50 ± 7.58.  Controls: BIT-C 98.20 ± 28.39, Difference 15.70 ± 7.36. Catherine Bergego Scale 10.60 ± 4.95, Difference −4.10 ± 1.73. FMA-UE 30.00 ± 7.90, Difference 9.50 ± 2.64. MBI. 71.40 ± 12.65, Difference 21.00 ± 8.89. WHODAS-2.0 107.80 ± 11.70, Difference −16.20 ± 6.99 |
| Choi^143^ | 2021 | Korea | Investigate the effects of VR-based digital practice program on unilateral spatial neglect rehabilitation in patients  with subacute stroke | RCT | 24  12 intervention  12 control | Digital practice for 30 minutes, 3 times/week for 4 weeks.  Participants wore Oculus Rift DK2 and Leap Motion (Leap Motion Inc., San Francisco, CA) and were seated in a chair (with a seat back and arm rest or wheelchairs) to perform 10 different applications from Oculus share and Leap Motion developers. Participants were instructed to perform the VR applications task with their non-affected hand | Conventional unilateral spatial neglect specific training for  30 minutes, 3 times a week for 4 weeks,.  structured  visual tracking, reading and writing, drawing and copying, and  puzzles | Intervention: MVPT-V Processing time (s) Intervention group pre-test: 5.52±0.17, post test: 3.36±1.19 p= 0.002. LBT Intervention group pre-test: 8.25±5.89, post test: 11.75±5.83 p= 0.002. CBS Intervention group pre-test: 8.33±5.87, post test: 11.25±5.03 p=0.003. MBI Intervention group pre-test: 37.42±8.73, post test: 47.17±9.73 p= 0.003.  Controls: LBT control group pre-test: 7.83±6.28 9.67±6.61, post test: p= 0.005; Between groups Intervention & control p= 0.020. CBS control group pre-test: 9.33±6.16, post test: 10.42±6.33 p=0.006; Between groups Intervention& control p=0.52. MBI control group pre-test: 38.08±9.80, post test: 44.50±10.19 p= 0.002; Between groups intervention & control 0.143 |
| Elshout^144^ | 2021 | The Netherlands | Explore 1) whether CMT results in greater attenuation of visuospatial neglect symptoms than VST. 2) to explore whether fixation patterns are  different for visuospatial neglect patients compared to healthy individuals and whether training can improve a potential fixation imbalance. 3) to explore whether the performance during the training task is predictive for training outcome as assessed during the visuospatial neglect tests | RCT | 20 | 10x 30-minute sessions.  CMT patients are instructed to make congruent eye and pointing movements during a game-like task on a touchscreen 15-inch laptop. A grid of nine empty circles (target grid) is placed beneath the template grid | 10x 30-minute sessions.  VST patients have to compare a template grid consisting of nine filled circles with different colours placed in their ipsilesional hemifield with a target grid. The instruction was to report how many items were differently coloured at each grid location (ranging from zero to a maximum of nine targets. | The CMT group had lower scores -which represent attenuation of neglect symptoms (a 6.25 lower Catherine Bergego Scale score) on post measurement. The VST group performed slightly worse Catherine Bergego Scale (3.23 higher Catherine Bergego Scale score). Of note, the groups were not directly compared. line bi-section tests: reliable data in CMT (n=6) & VST (n=6).  Fixation index CMT (mean 0.94, SE 0.19); VST (mean 0.46, SE 0.14) both within normal range prior to training (t (11) = 1.716, p =0.06; t (11)=-0.716, p=0.25) with respect to the healthy control group (mean 0.57, SE = 0.1). Fixation index CMT was reduced to 0.67 (SE 0.09), and in the VST group increased to 0.62 (SE 0.15). Both were not different from the healthy controls (CMT: t (11) =0.726, p=0.24; VST: t (11) =217, p =0.42). In the CMT group, four of the six patients showed a more balanced fixation index, whereas in the VST group only one patient showed a more balanced fixation index |
| Park^145^ | 2021 | Korea | Investigate the effects of the robot-assisted hand training on hemispatial neglect of older patients with chronic stroke | RCT | 24 | The experiment group performed 20 30-minute sessions (five days a week for four weeks) of robot-assisted hand training using the Amadeo Robotic device. The participant's hand motion was assisted by the robot and adjusted to the individual's level of function through the assistive therapy mode of the Amadeo robot. During the training, the participants in the experiment group received visual feedback of their hand movements via video animation presented on a monitor | The control group received the 20 30-minute sessions of the conventional treatments that lasted 30 minutes each session for hemispatial neglect symptoms. These treatments included VST using a prism and vibration stimulation applied on the left neck extensors and a middle part of the left forearm | After intervention, both groups showed a significant improvement in the Catherine Bergego Scale. On the other hand, there was a statistically significant difference in changes in the Catherine Bergego Scale (p<0.001; h2=0.569). This finding indicated that robot-assisted hand training was more clinically beneficial in reducing hemispatial neglect symptoms in the participants’ activities of daily living.  Improvements in the line bisection test, the Albert test, and the Catherine Bergego Scale were found in the experimetral group whereas there were significant improvements in the line bisection test and the Catherine Bergego Scale but not the Albert test in the controls. In addition, the experimental group showed a significantly greater gain in all outcome measures compared to the controls (p<0.05) |
| Mizuno^146^ | 2021 | Japan | Investigate how PAT affects ADL and self-awareness in subacute USN patients | RCT | 34 | PAT, repetitive pointing task under a table to hide their hand trajectories 90 times with prism glasses that shifted their visual field 12 to the  right. Patients underwent 2x 20-minute daily sessions, 5 days a week for 2 weeks, for a total of 20 sessions | Sham: same task as intervention group but wearing neutral glasses | Two of ten items (gaze orientation and exploration of personal belongings) were significantly improved in the prism group compared with those in the control group. The absolute value of the anosognosia score was significantly improved by PAT.  Catherine Bergego Scale: Scores of gaze orientation and the personal belongings were significantly lower in the prism group than in the control group at T2. The other items were not significantly different between the prism and control groups at each time point |
| Szalados^161^ | 2021 | UK | Investigate the clinical effectiveness of Eye-Search, a web-based therapy app designed to improve visual search times, in a large group of patients with either hemianopia, neglect or both hemianopia and neglect | Cohort | 426 | The therapy is a ramp-step pursuit paradigm in which participants have to follow a “rolling ball” stimulus that contains a “C” shape and moves in two phases. The therapy moves through 16 progressively harder levels of 300 trials as static distractor stimuli are added in, and the contrast between the target and background is reduced | N/A | Patients with hemianopia alone, or with neglect plus hemianopia, showed an improvement in self-reported measures of visual search: “finding things”. The neglect only group showed no significant improvements on either the impairment or self-reported measures of visual search. Regarding the five other ADLs these results are largely confirmatory of their previous study. There was a main effect of therapy across the four groups for “Shopping”, “Meal Preparation” and, “Collisions” but no significant effect of therapy on “Hygiene” and “Getting Lost”, indicating that Eye-Search does not have a generalized effect on self-reported visuospatial functions |
| Vilimovsky ^148^ | 2021 | Czech Republic | Evaluate the efficacy of PAT on visuo spatial symptoms of spatial neglect in an inpatient rehabilitation setting that offered a highly intensive comprehensive brain injury rehabilitation program | RCT | 23 | PAT + intensive standard care. 10 sessions of 15-20 minutes treatment, over two weeks.  goggles fitted with 20-diopter prism lenses that shift the visual field to the ipsilesional side of space for 11.4 degrees of visual angle.  Participants completed 60 visuomotor movements while the first part of arm movements was blocked from view. Stimuli were pseudo-randomly presented either at body midline or in left or right space (32.1 cm to the side of body midline). The task was to mark the centre of a line or cross out a circle | Sham visual adaptation training + intensive standard care.  10 sessions of 15-20 minutes treatment, over two weeks.  Same training process as intervention group but wearing flat neutral goggles | Both groups improved from baseline to follow-up to the similar extent, and there was no specific effect of PAT.  While SN symptoms reduced in both groups, we found no difference between the two groups in the degree of improvement. In addition, the average spatial neglect recovery rates were 39.1% and 28.6% in the PAT and Sham groups, respectively, but this discrepancy did not reach statistical significance |
| Choi^149^ | 2022 | Korea | Investigate the effects of a complex rehabilitative  programme that integrates PAT and neck vibration for unilateral neglect in patients of chronic stroke | RCT | 36 | Group a; 30 min of conventional occupational therapy + 20 min of neck vibration and PAT on the affected neck extensor. 5 times per week for 50 min/day, for a total of 20 times  during a 4-week rehabilitation period.  Group b; 30 min of conventional occupational therapy + 20 min of neck vibration on the affected neck extensor. 5 times per week for 50 min/day, for a total of 20 times  during a 4-week rehabilitation period | The conventional occupational therapy consisted of joint exercises, task-oriented training, and ADL training. 30 min of conventional occupational therapy + 20 min of prism adaptation. 5 times per week for 50 min/day, for a total of 20 times  during a 4-week rehabilitation period | All three groups exhibited a reduction in unilateral neglect and an improvement in activities of daily living after the intervention (p<0.05). Notably, Group A (PAT + neck vibration) exhibited a significantly greater level of reduction in unilateral neglect than the other groups (p<0.05); however, the improvement in ADL did not significantly vary across the three groups (p>0.05).  On the intergroup comparison, Group A exhibited a trend of a higher increase in MBI, although this was not significant (p<0.05). For the scores of Albert’s test and the Catherine Bergego Scale, significant intergroup differences were found (p<0.01 and p<0.05, respectively); there were non-significant differences in the comparison between groups B and C and there were significant differences with Group A |
| da Silva^150^ | 2022 | Brazil | Examine the effects of physical therapy after anodal tDCS and cathodal  tDCS to improve visuospatial and functional impairments  in individuals with USN after stroke | RCT | 51 | 2 times per week for 7.5 weeks. A constant current of 1mA was delivered for real stimulation for 20 minutes. This was followed by 1 hour of physical therapy.  For cathodal tDCS, the cathode was placed over P3, and the anode was placed over the right supraorbital area. A constant current of 1mA was delivered for real stimulation for 20 minutes. This was followed by 1 hr of physical therapy | 2 times per week for 7.5 weeks. For the sham condition, the stimulator was turned on, and the current intensity was gradually increased for 30 seconds and tapered off over 30 seconds. This was followed by 1hr of physical therapy | There was no significant difference between groups on the Catherine Bergego Scale (F2,46 = 0.28, p=0.756), the modified Rankin Scale (F2,46 = 0.12, p=0.663), the Barthel Index (F2,46 = 0.20, p=0.617) or the FIM (F2,46 = 0.14, p = 0.866). A significant change was seen for the Behaviour Inattention test from baseline to post-intervention. |
| Fong^151^ | 2022 | China | Investigate the effects of MT, with reference to using a  glass wall or a covered mirror, on the reduction of spatial neglect for patients with stroke | RCT | 21 | The patients in the MT group watched the movements of the non-affected arm in the mirror and actively tried to imitate them with the affected limb, synchronizing it with the reflection. Patients performed bimanual upper limb exercises (5 table-top tasks) with graded levels of difficulty. Four sessions per week for three weeks, each lasting for 30 minutes | In the sham 1 group, the patient followed the same protocol as in the MT group but with a direct view of the affected arm through a transparent glass wall.  In the sham 2 group, the mirror was covered by a cloth and the patient was instructed to move both arms while looking at a cross mark on the covered mirror, while imagining the analogous movements of the affected arm | In regard to the Catherine Bergego Scale, the mixed-effects model demonstrated significant time effects in all three groups (all p<0.05). However, no significant group-by-time interaction effect was noted.  The results showed that there was no significant advantage for MT than sham 1; however, MT was more beneficial than sham 2, as shown by the line crossing (p=0.022). Improvement in discriminating the left-gap figures on the left and right side of the page in the Gap Detection Test was greater in MT than using the covered mirror (p=0.013; p=0.010), showing a slight advantage of MT in alleviating allocentric symptoms |
| Gillen^162^ | 2022 | USA | Examine the feasibility and  efficacy of PAT in improving symptoms of spatial neglect and whether this increases the likelihood of meaningful functional recovery | Case control cohort | 74;  37 intervention  37 control | PAT sessions lasted around 30min during which patients with left-sided neglect on 20 dioptres deviating their visual field to the right while aiming their finger at a series of visual targets.  Patients were administered PAT as a function of treatment team preference | Standard care; customary strategies for neglect treatment (e.g., visual scanning training, limb activation) | PAT;  Intervention; Catherine Bergego Scale improvement median 4 (IQR:0–6).  - Mild Neglect 0 (−1 to 2)  - Moderate Neglect 4 (1 to 6)  - Severe Neglect10 (4 to 14).  FIM improvement median 22 (IQR 12–31)  - Mild Neglect20 (10–32)  - Moderate Neglect 21 (13–28)  - Severe Neglect 25 (13–32)  Controls;  Catherine Bergego Scale improvement median 2 (IQR 0–9)  - Mild Neglect: 1 (0 to 6)  - Moderate Neglect: 8 (0 to 10)  - Severe Neglect: 2 (0–13)  FIM improvement median 17 (IQR 10-28)  - Mild Neglect: 24 (13 to 35)  - Moderate Neglect: 24 (15 to 33)  - Severe Neglect: 10 (7–17) |
| Scheffels^152^ | 2022 | Germany | Investigate whether age, aetiology, severity of motor impairments, and visual field deficits affect the efficacy of PAT | RCT | 23 | Therapy sessions lasting 30-40 minutes were offered for 15 days, with one session per day and with the weekends off. In PAT-intermittent, goggles were put on two (first recruitment series) or three times (second series). Pointing movements and time with goggles on were kept constant across the two protocols. Goggles always shifted the visual field 10 degrees to the right | Therapy sessions lasting 30-40 minutes were offered for 15 days, with one session per day and with the weekends off. In PAT-continuous, wedge-shaped prism goggles were worn continuously during the entire session | They significantly improved on all outcome measures, Apples Cancelation Test (p<0.001), text reading [p=0.002], Line Bisection Test [p=0.010], Clock Drawing Test (p=0.050), spontaneous body orientation [p<0.001], cued body orientation [p=0.010], ERBI [p<0.001], and FIM [p<0.001] |
| Sim^153^ | 2022 | Korea | Compare the effectiveness of bimanual MT and unimanual MT, the two protocols of mirror therapy, for the reduction of the symptoms of unilateral neglect in stroke patients | RCT | 30 | Bimanual MT: Five 30-minute sessions of MT per week for four weeks | Unimanual MT (with non-paretic upper limb). Five 30-minute sessions of MT per week for four weeks | The results of star cancellation test, line bisection test, picture scanning test and Korean - Catherine Bergego Scale showed significant decreases in unilateral neglect in both groups (p<0.05). Korean MBI improved significantly in both groups (p<0.05). There were significant differences between the two groups in the unilateral neglect tests (p<0.05), but no significant difference in ADL evaluation (p>0.05) |
| Umeonwuka^154^ | 2022 | South Africa | Investigated predictors of USN recovery after prism therapy at the sub-acute phase of recovery. | RCT | 74 | PAT + standard care.  12 training sessions (one 30-minute session a day), every weekday over a period of 16 days.  The patients were instructed to reach and return their hands as quickly as possible without being corrected for pointing errors. Then, wearing 20 dioptre prism glasses that shifted their visual field 11.4° to the right, the task was repeated 90 times. After pointing at targets, the prism glass was taken off and they pointed 60 more times | Sham prism adaptation + standard care.  12 training sessions (one 30-minute session a day), every weekday over a period of 16 days. | For the Catherine Bergego Scale, univariate analysis showed only treatment allocation had a significant relationship with the Catherine Bergego Scale being lower by an average of 5.65 in the intervention group compared to the score of participants in the control arm. They estimated that MMSE influences recovery post-stroke with Catherine Bergego Scale as an outcome variable.  Intervention participants had improved total scores of the BIT-C at post-treatment compared to pre-treatment. Stroke survivors in the treatment group had 18.62 times more odds of recovery than patients in the control group.  Controls had improved total scores of the BIT-C at post-treatment compared to pre-treatment. |
| Vilimovsky ^163^ | 2022 | Czech Republic | Determine whether the integration of  PAT into a high-intensity rehabilitation program predicted reduced adverse effects of spatial neglect to  the extent that patients who presented with spatial neglect at the time admission were able to achieve  a similar level of motor outcomes and functional recovery as patients without spatial neglect | Cohort | 342  71 with neglect  271 without neglect | In addition to intensive standard care, the spatial neglect+ group received PAT. Each PAT session lasted 15–20 min and required the patient to perform 60 arm-reaching movements while wearing 20-diopter prism lenses that shifted the visual field to the ipsilesional side for 11.4 degrees of the visual angle | Stroke survivors without neglect; standard care | Although 71 patients (85%) received PAT, the presence of spatial neglect at baseline, regardless of PAT completion, was associated with lower functional independence, higher risk of falls, and a lower functional level of the affected upper limb both at admission and at discharge. The severity of spatial neglect was associated with inferior rehabilitation outcomes. Nonetheless, patients with spatial neglect who received PAT had similar rehabilitation gains compared to patients without spatial neglect. Thus, the present study suggests that integrating PAT in an intensive rehabilitation program will result in improved responses to regular therapies in patients with spatial neglect. |
| Bode^155^ | 2023 | Germany | Evaluate the effects of combined OKS and READ on the remission of hemispatial neglect following stroke | RCT  Cross  Over | 20 | Active intervention (I) was a combined treatment consisting of 15 daily 45-minute sessions of OKS (stimuli moving from the ipsi- to the contralesional side to elicit pursuit eye movements to the contralesional field) and READ (reading therapy assisted by an instructor providing endogenous and exogenous cues) | Neuropsychological treatment provided by the same therapist not targeting visuospatial attention | Overall performance in the neglect test battery improved slightly more after OKS READ than after CONTROL (d=6%; p=0.002). The remission of neglect-related functional disability did not differ between treatments (d=-2; p=0.291). Ipsilesional fixation bias during free viewing was the only secondary outcome that was improved by OKS READ as compared to CONTROL (d= -2.8°; p=0.005) |
| Longley^156^ | 2023 | UK | Investigate feasibility and acceptability of PAT for people with inattention  , early after stroke, during usual care. | RCT | 39 | For participants in the intervention arm, prism adaptation  training was offered once a day at the start of routine occupational therapy sessions, for up to three  weeks, five days a week. The training lasted no more  than 5 min plus set up time. Participants wore 25 dioptre  (12.5°) wedge prism glasses adjusted for left or right sided inattention as appropriate | Standard care; The control group received standard occupational therapy (with no PAT),  using the same type of patient-facing activities as the intervention group. | Prism adaptation training was generally well recorded and delivered as intended: it took a median of 4.7 min (IQR 4.1-5.0) and participants made approximately 71.4 (SD 20.0) pointing movements per session. Participants received a median of eight sessions (IQR: 5-12). Of 322 prism adaptation training sessions offered, only 11 (3%) were declined.  Attrition was low with three-week outcomes collected from 47 (89%) and 12-week outcomes from 39 (74%) patient participants. Research support staff completed 23 (49%) of the three-week outcomes; and 11/39 (28%) of the 12-week assessments.  There was no signal that patients allocated to intervention did better than controls |

ADL (activities of daily living); BIT (Behavioral Inattention Test); BIT-C (Behavioral Inattention Test-conventional section); CMT (congruent movement training); cTBS (continuous theta bust stimulation); ERBI (early rehabilitation Barthel Index); FAM (Functional Assessment Measure) FES (functional electrical stimulation); FIM (Functional Independence Measure); FMA-UE (Fugl Meyer Assessment – Upper Extremity); HEP (hemifield eye patching); KF-NAP (Kessler Foundation Neglect Assessment Process); MAC (mobility assessment course); MBI (modified Barthel Index); MMSE (Mini Mental State Examination); MT (mirror therapy); MVPT (Motor-free Visual Perception Test); MVPT-V (Motor-free Visual Perception Test – Vertical); OKS (optokinetic stimulation); PAT (prism adaptation treatment); RAT (Robot-assisted Arm Therapy); RCT (randomised controlled trial); READ (cueing-assisted reading therapy); rTMS (repetitive Transcranial Magnetic Stimulation); SINGER (Scores of Independence Index for Neurological and Geriatric Rehabilitation); tDCS (transcranial Direct Current Stimulation); USN (unilateral spatial neglect); VR (virtual reality); VSS (Visuospatial Scale); VST (visual scanning therapy); WHODAS (World Health Organisation Disability Assessment Scale).

**Supplementary table 17: PICO 13: For adults with other visual perceptual disorders due to stroke, does compensatory, substitute or restitutive intervention, compared to no intervention, improve activities and quality of daily life?** **Intervention results.**

| **Author** | **Date** | **Country** | **Aim** | **Design** | **Sample** | **Intervention** | **Control** | **Results** |
| --- | --- | --- | --- | --- | --- | --- | --- | --- |
| Park^165^ | 2015 | Korea | Investigate the effects of a Korean computer-based cognitive rehabilitation program on the cognitive function and visual perception ability of patients with acute stroke | RCT | 30:  15 intervention  15 sham | Computer-based cognitive rehabilitation program with the CoTras program.  20 sessions (5 days a week for 4 weeks) of computer-based cognitive rehabilitation program.  CoTras consists of a diverse training program including visual perception, attention, memory, orientation, and others (categorization, sequencing) | Conventional cognitive rehabilitation.  30 min sessions (5 days a week for 4 weeks).  pencil and paper with emphasis on visual perception ability | After treatment, the Lowenstein Occupational Therapy Cognitive Assessment and MVPT scores, measuring the cognitive function of both groups significantly increased (p<0.05), and there was a statistically significant difference between both groups at the end of treatment (p<0.05) |
| Kim^126^ | 2016 | Korea | Examine the effects of tDCS on visual perception and performance of activities of daily living in patients with stroke | RCT | 30:  15 intervention  15 sham | Five times per week, 30 minutes each, for six weeks.  Traditional occupational therapy and tDCS were applied. In order to stimulate the occipital lobe, the anode was attached to C3 (central 3) and the cathode was fixed to the required stimulation area by winding a strap around the supraorbital area. A current of 1 mA was applied for 20 minutes | Five times per week, 30 minutes each, for six weeks.  Traditional occupational therapy and sham tDCS were applied.  Electrodes were applied to the same location for the same length of time as above, but the current was discontinued after 30 seconds | Comparison of the MVPT results before and after the intervention revealed a statistically significant difference (p<0.05). Results of the MVPT showed that the experimental group demonstrated a significant improvement of approximately six points. The control group also significantly improved in visual perception after the intervention, increasing from 21.0 ± 3.9 points to 23.9 ± 3.8 points. The total FIM score showed that the experimental group increased by 13 points after the intervention. The FIM score of the control group increased by three points. Both increases were statistically significant (p<0.05) |
| Kim^166^ | 2017 | Korea | Investigate the effects of rTMS on visual perception, depression, and ADLs in stroke patients | RCT | 44:  22 intervention  22 sham | All subjects underwent treatment for 20 minutes, 5 times per week, for 4 weeks.  Low frequency rTMS stimulus was targeted at P3, over the left healthy side using a 1-Hz stimulus at 90% threshold 4 times for 5 minutes at a time, separated by 1-minute intervals. High frequency rTMS was applied at P4, over the right affected side, using a 5-Hz stimulus at 90% threshold, 20 times for 5 seconds at a time, separated by 55-second intervals.  Conventional rehabilitation therapy was performed after the procedure | All subjects underwent treatment for 20 minutes, 5 times per week, for 4 weeks.  Mock stimulus used the same protocol as low-frequency rTMS, except that the coil was not placed against the skull, and the stimulus was applied in the vertical direction.  Conventional rehabilitation therapy was performed after the procedure. | rTMS had a significant effect on depression, visual perception, and ADLs in the experimental group, and there were also significant differences between the effects in week 1 and week 4, and between the effects in week 4 and week 8 (p<0.05). In the control group, there was no difference between the effects in week 1 and week 4. There was a significant difference in depression and visual perception in week 8 compared to Week 1, but there was no significant difference in ADLs (p<0.05) |
| Choi^167^ | 2022 | Korea | Investigate the effects of task-oriented training on the improvement of upper-limb functions, visual perception, and ADL performance in acute stroke patients by designing the training based on the types of tasks that anyone needs to perform in daily life | RCT | 24:  12 intervention  12 standard care  Completed:  10 intervention  10 standard care | 30 minutes a day, five times a week, for 6 weeks.  Task-oriented training using tasks performed in daily life, those with a difficulty level between 0.2 and 0.7 that could be performed by any patients in the hospital and at home were selected from among the standardized tasks listed in the Assessment of Motor and Process Skills. Patients performed each item for 5 minutes, with a 1 minute preparation time between tasks | 30 minutes a day, five times a week, for 6 weeks.  Therapeutic tools frequently used in occupational therapy were selected according to the definition of table-top activities. The task difficulty was based on patient performance level | MVPT-V no significant interaction was found between the group and time (p=0.141), and no statistical group difference was found either (p=0.735). Both groups showed significant improvement in the MVPT-V score after the intervention (27.8 ± 2.0 to 32.7 ± 2.5, p<0.001 and 28.3 ± 1.3 to 31.7 ± 1.9, p<0.001, respectively).  Korean-MBI. a significant group-by-time interaction effect was found in the mean MBI score (p=0.013). The main effect of the group was not significant (p=0.657), and no significant differences between the groups were found before or after the intervention (p=0.615 and 0.150, respectively). The MBI score in the task-oriented training group significantly improved, from 55.9 ± 14.4 to 80.9 ± 12.3 (p<0.001). Likewise, the score in the control group significantly increased, from 59.2 ± 14.5 to 72.5 ± 12.6 (p<0.001) |

ADL (activities of daily living); FIM (functional independence measure); MBI (modified Barthel Index; MVPT (Motor-free Visual Perception Test); MVPT-V (Motor-free Visual Perception Test – Vertical version); rTMS (repetitive Transcranial Magnetic Stimulation); tDCS (transcranial Direct Current Stimulation.
